# The nonexistence of a paddlewheel effect in superionic conductors

**DOI:** 10.1073/pnas.2316493121

**Published:** 2024-04-24

**Authors:** KyuJung Jun, Byungju Lee, Ronald L. Kam, Gerbrand Ceder

**Affiliations:** ^a^Department of Materials Science and Engineering, University of California, Berkeley, CA 94720; ^b^Materials Sciences Division, Lawrence Berkeley National Laboratory, Berkeley, CA 94720; ^c^Computational Science Research Center, Korea Institute of Science and Technology, Seoul 02792, Republic of Korea

**Keywords:** superionic conductors, diffusion, ab initio molecular dynamics, correlated motion, solid electrolyte

## Abstract

Since the 1980s, the paddlewheel effect has been suggested as a mechanism to boost lithium-ion diffusion in inorganic materials via the rotation of rotor-like anion groups. However, it remains unclear whether the paddlewheel effect, defined as large-angle anion group rotations assisting Li hopping, indeed exists; furthermore, the physical mechanism by which the anion-group dynamics affect lithium-ion diffusion has not yet been established. In this work, we differentiate various types of rotational motions of anion groups and develop quaternion-based algorithms to detect, quantify, and relate them to lithium-ion motion in ab initio molecular dynamics simulations. Our analysis demonstrates that, in fact, the paddlewheel effect, where an anion group makes a large angle rotation to assist a lithium-ion hop, does not exist and thus is not responsible for the fast lithium-ion diffusion in superionic conductors, as historically claimed. Instead, we find that materials with topologically isolated anion groups can enhance lithium-ion diffusivity via a more classic nondynamic soft-cradle mechanism, where the anion groups tilt to provide optimal coordination to a lithium ion throughout the hopping process to lower the migration barrier. This anion-group disorder is static in nature, rather than dynamic and can explain most of the experimental observations. Our work substantiates the nonexistence of the long-debated paddlewheel effect and clarifies any correlation that may exist between anion-group rotations and fast ionic diffusion in inorganic materials.

## Significance

Inorganic materials that allow fast lithium-ion diffusion play a critical role as solid electrolytes in all-solid-state batteries. For decades, the paddlewheel effect, in which lithium-ion diffusion is accelerated by the rotational motion of anion groups within the crystal structure, has been suggested. However, there has been a lack of understanding of the types of rotational motions that exist in these materials and the existence of any coupling between the rotational motion of anion groups and lithium hops. Here, we find that the paddlewheel effect, following its literal definition, does not exist. Rather, we prove that anion groups can better tilt their orientations in response to migrating lithium to improve ionic conductivities, which we call the soft-cradle effect.

Superionic conductivity is a crucial aspect of solid electrolytes which are increasingly seen as a promising alternative to conventional liquid electrolytes due to their potential for enabling batteries with high energy density, long cycle life, and improved safety (1). Significant progress has been made in creating novel solid-state conductors by understanding their diverse diffusion mechanisms that allow fast ion transport comparable to that in a liquid medium (2–11).

One aspect that has been historically emphasized is the role of the rotational motion of anion groups in boosting ion transport, also known as the “paddlewheel effect.” Since the 1980s, there has been intense debate on the existence of the paddlewheel effect, and whether it is a valid mechanism to improve ionic conductivity (9, 12–20). This mechanism was first proposed to explain the high lithium ionic conductivity in the high-temperature (HT) Li_2_SO_4_ phase and was subsequently suggested to occur in many other systems (21–25). However, the physical definition of a paddlewheel effect, or how it specifically assists Li motion has not been clearly established. Some researchers refer to the paddlewheel effect in a broad sense as the rotational motion of anion groups accelerating/aiding the motion of lithium ions. Others specifically refer to it in a more literal sense, where anion groups boost lithium-ion diffusion by rotating like a paddlewheel or a revolving door to deliver momentum to lithium ions (14). The type of rotational motion responsible for such an effect and the physical mechanism by which it may improve the ionic conductivity have been left ambiguous, which has led to imprecise and inconsistent usage of the term and limits our ability to quantitatively account for it in the design of novel conductors (26).

As more diverse classes of materials are investigated for superionic conductivity, the paddlewheel effect has been proposed to rationalize fast lithium- or sodium-ion diffusion in various ionic conductors such as amorphous (22) and crystalline (25) lithium thiophosphates (Li_3_PS_4_), Na_3_PS_4_ (27), Na_11_Sn_2_PS_12_ (24), lithium borohydrates, and closo-borates (28), antiperovskites (29). Experimental methods such as the maximum entropy method (24, 25) analysis on neutron diffraction data, quasi-elastic neutron scattering (QENS) (30–34) and computational methods such as the angular correlation function or Helmholtz free energy maps (24, 25, 35) have been used to argue for the existence of the paddlewheel effect by confirming some kind of anion-group rotational motion or rotational disorder. However, a fundamental drawback of such analyses is that they only offer time- or space-averaged positional information for lithium ions and anion groups. As a result, these analyses fail to capture any spatial and temporal correlation that may exist between lithium-ion hop events and anion-group rotation events, which is essential to understand the mechanistic relationship between them.

In this work, we use computational methods to analyze various materials’ classes where the existence of paddlewheel effect has been suggested. Event-detection algorithms are used to identify anion-group rotational events as well as lithium-ion translational events from large-scale ab initio molecular dynamics trajectories. Throughout this work, we use the paddlewheel effect in a literal sense, referring to lithium-ion hops that are assisted by an adjacent anion-group simultaneously making a large angle rotation. We identify three types of rotational motion of anion groups in inorganic crystals and reveal how they are related to lithium-ion diffusion. Based on such data, we conclude that fast Li-ion conduction is not assisted by large-angle rotations of anion group polyhedron, as implied by the paddlewheel. Rather, we find that small, static reorientations of anion groups around Li soften the energy landscape and can enhance Li migration. Such local relaxation around mobile ions is not unusual and is found in many fast ion conductors whether they contain anion groups or not.

## 1. Results

### 1.1. Quaternion-Based Detection of Rotational Events.

We collect large-scale atomistic trajectories by performing ab initio molecular dynamics (AIMD) simulations spanning tens of nanoseconds (simulation details in *SI Appendix*, *Note S1* and Table S1 and Fig. S1) for several materials claimed to exhibit the paddlewheel effect: crystalline (α, β, γ) Li_3_PS_4_, amorphous Li_3_PS_4_ of density 1.8 and 2.0 g/cm^3^, and the HT-Li_2_SO_4_ phase (20, 22, 25). From these trajectories, we track each anion-group (e.g., PS_4_, SO_4_) to detect the occurrence of rotational events, specifically tracking their angle, geometric location, and time. Similarly, for each lithium ion, we detect the occurrence of each translational hopping event, its hopping distance, and the location and time of the event. This detailed temporal and spatial information on Li hopping and anion-group rotation will enable us to clearly establish whether they occur in coordination or not.

The rotation of an anion group was tracked by using quaternion coordinates which are well suited to study rotational trajectories. Quaternions (36, 37) are a combination of a scalar and a vector and are expressed as a+bi+cj+dk, where *a*, *b*, *c*, and *d* are real numbers and i, j, and k are unit vectors in orthogonal axes. Quaternions provide a compact, smooth, and numerically stable representation to capture three-dimensional rotational operations [or SO(3) group] on any arbitrary axis, as compared to Euler angles or a rotation matrix (38).

**Fig. 1.**
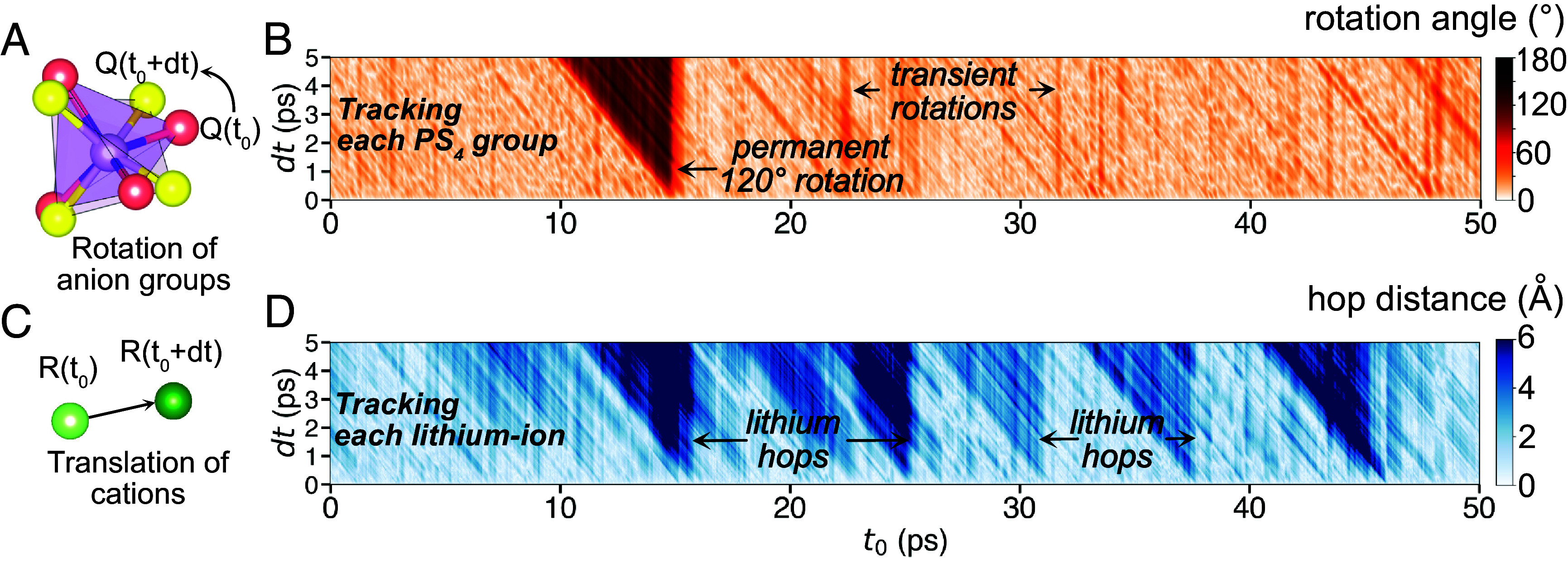
Rotational and translational event-detection algorithm. (*A*) Quaternion representation of the orientation of anion groups. (*B*) Rotation diagram representing the rotational motion of a single PS_4_ tetrahedron in β-Li_3_PS_4_ at 900 K. The color intensity gives the minimum angular distance between the quaternion of a PS_4_ group at t_0_ and t_0_ + dt. (*C*) Illustration of tracking of the translational motion of cations. (*D*) Diagram representing the translational motion of a single lithium ion in β-Li_3_PS_4_ at 900 K. Q and R in (*A*) and (*C*) represent the quaternion of an anion-group and the positional vector of a lithium-ion, respectively.

The Fig. 1*B* shows what we refer to as a rotation diagram for a single PS_4_ group. The diagram relates the rotational state of the anion group at time t_0_ to its state at t_0_ + dt. The color intensity indicates the amount of angular rotation that would be needed to bring the anion group back from its state at t_0_ + dt (y-axis) to its orientation at t_0_. Several noticeable patterns appear in this diagram. The sawtooth-shaped large-angle region emanating from (t_0_, dt = 0) indicates a permanent large-angle rotation occurring at t_0_ because for an extended period of time the rotational state at t_0_ is different from that at t_0_ + dt. For example, in Fig. 1*B*, a rare PS_4_ rotation of 118.1° is detected in the β-Li_3_PS_4_ trajectory at 900 K, specifically at t_0_ = 14 ps. In contrast, sawtooth-shaped stripes of large angles, such as t_0_ = 22 ps in Fig. 1*B* shows a rotational change which is only present for a very specific dt, indicating a transient large angle rotation which immediately returns back to its original orientation. Finally, the nonzero background signals in the rotation diagram (bright region in Fig. 1*B*) correspond to the librational motion of a PS_4_ group (rapid and small-angle vibrational events).

We track the translational motion of lithium ions in a similar fashion. A translation diagram (Fig. 1*D*) shows the shortest distance to bring back a lithium ion from its position at t_0_ + dt to t_0._ Large sawtooth-like signals in the translation diagram correspond to lithium-ion hopping events, whereas the background signals capture the vibrational motion of lithium ions. Our method of analyzing rotational or translational displacement within a time frame dt throughout the entire trajectory allows the detection of each individual event and its characteristics without spatial and temporal averaging. Exemplary rotation and translation diagrams for each model system at various temperatures are shown in *SI Appendix*, Figs. S3–S12, with each diagram representing a 100-ps segment of a much longer simulation.

### 1.2. Frequency of Hops and Rotation of Various Angles.

**Fig. 2.**
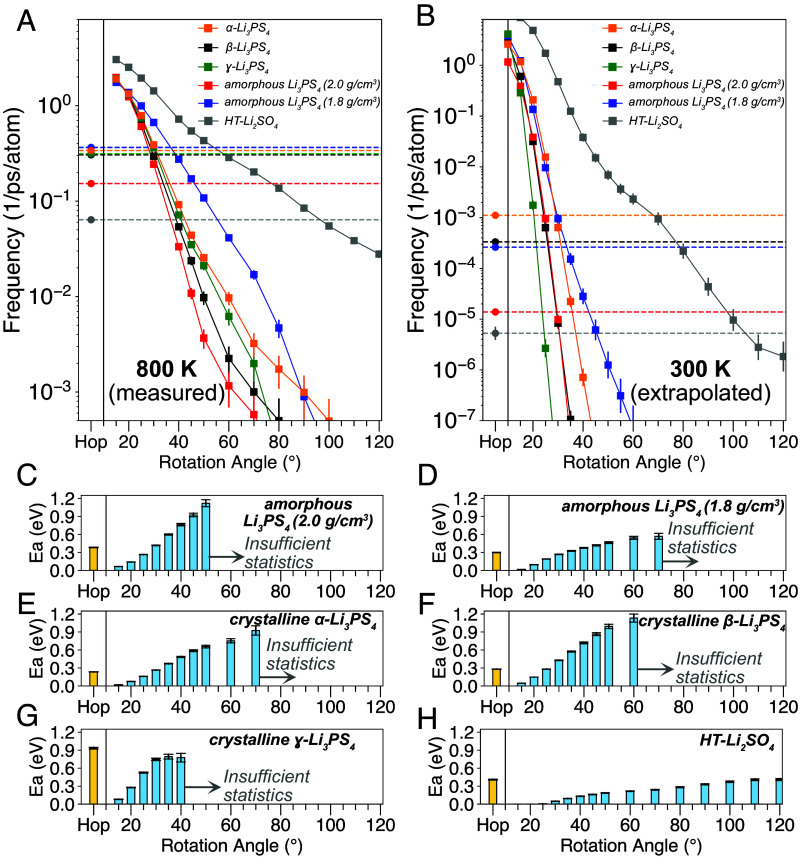
Anion-group rotation and lithium-ion hop frequency of superionic conductors. Frequencies of hop and rotation events of various angle cutoffs in AIMD simulations at 800 K (*A*) and extrapolated to 300 K (*B*). Square symbols and solid lines are rotation events, and the horizontal dashed lines represent the rate of Li hopping. Activation energies of hop events (orange) and rotation events (blue) of various angle cutoffs for (*C*) amorphous Li_3_PS_4_ of density 2.0 g/cm^3^, (*D*) amorphous Li_3_PS_4_ of density 1.8 g/cm^3^, (*E*) crystalline α-Li_3_PS_4_, (*F*) crystalline β-Li_3_PS_4_, (*G*) crystalline γ-Li_3_PS_4_, and (*H*) HT-Li_2_SO_4_ phase. The activation energies of large rotation angle cutoffs are not plotted if there were insufficient number of rotational events to compute the activation energy. The hop frequencies and rotation frequencies are normalized by the number of lithium-ion and anion groups, respectively.

Using our event-detection algorithms, we detect lithium-ion hop events as well as anion-group rotation events of various cutoff angles and collect their event frequencies as a function of temperature for each system. Fig. 2*A* shows the frequency at which rotations of a given angle cutoff occur as well as the frequency of Li-ion hops (dashed horizontal line) for a set of conductors at 800 K. A similar analysis was performed at other temperatures with the data shown in *SI Appendix*, Fig. S2 and *Note S1*. Based on the rotation frequencies as a function of temperature, we can extract an activation energy for rotational events of a given angle cutoff. The results of this analysis are shown in Fig. 2 *C*–*H*. Except for HT-Li_2_SO_4_, the activation energy for large angle rotations approaches 1 eV in all conductors. Using the activation energies, we extrapolate all event frequencies to 300 K as shown in Fig. 2*B*. Several important findings emerge from this data.

We find that for the crystalline and amorphous Li_3_PS_4_ systems, any rotation events greater than 60° are extremely rare even at high temperature (800 K in Fig. 2*A*, other temperatures in *SI Appendix*, Fig. S2). For all of the fast conductors, lithium-hop events occur orders of magnitude more frequently than rotation events greater than 60° at 800 K. Because the activation energies of hops for fast conductors (Fig. 2 *C*–*F*) are significantly lower than those of rotation events greater than 45°, the discrepancy between the frequency of lithium-hop events and rotational events of the anion groups only becomes larger when extrapolated to 300 K (Fig. 2*B*). For a paddlewheel mechanism to make a significant contribution to Li hopping, a similar event frequency and activation energy between rotational motion and translational motion would be required. Hence, our data disproves the existence of the paddlewheel effect and any beneficial effect of large-scale anion-group rotation on Li migration. Large rotations of anion groups simply do not occur frequently enough to make contribution to Li hopping. Smaller rotation events of angle cutoff of 25° to 35° in the fast-conducting phases (Fig. 2 *C*–*F*) have similar activation energy and event frequency as the hop events. This finding raises the possibility that lithium-ion diffusion may instead be related to small-angle rotational motion of this range, which is investigated in the following section.

An important observation from Fig. 2 is that although β- and α-Li_3_PS_4_ are known to allow much faster lithium-ion diffusion than the poorly conducting γ-Li_3_PS_4_ (25, 39), all three polymorphs have high activation energies for large-angle rotations (>50°). This result is consistent with our conclusion that the large-angle rotational motions are not relevant for the lithium-ion mobility in the Li_3_PS_4_ polymorphs. The slightly lower activation energy of rotational motion for α-Li_3_PS_4_ may originate from its lower density (1.80 g/cm^3^) compared to that of β- and γ-Li_3_PS_4_ (1.83 and 1.91 g/cm^3^, respectively). Similarly, when comparing amorphous Li_3_PS_4_ of 1.8 g and 2.0 g/cm^3^, we find that lower density results in a slightly reduced activation energy for large-angle rotation events, but this value is still much higher than the barrier for Li-hops.

### 1.3. Understanding the Rotation-Energy Barrier of Anion Groups.

**Fig. 3.**
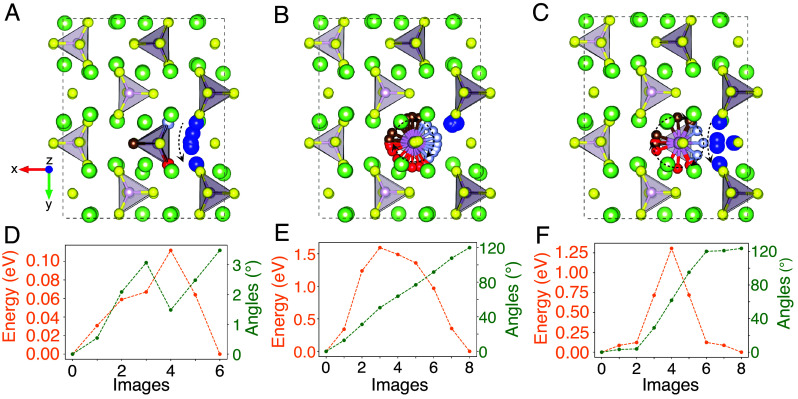
Minimum energy path of anion-group-assisted lithium-ion hops. (*A*) Lithium-ion hop from 8d site to a vacant 8d site in β-Li_3_PS_4_. (*B*) A PS_4_ tetrahedron making a threefold symmetry rotation. (*C*) Lithium-ion hop with a coherent PS_4_ threefold symmetry rotation. The yellow, green, and purple circles represent sulfur, lithium, and phosphorus atoms, respectively. The blue circle represents the lithium ion in the 8d site that is migrating. The red, brown, and light-blue circles and bonds represent the coordinating S^2−^ of the PS_4_ that is rotating. (*D*–*F*) Energy barrier (orange) and angle of PS_4_ from the initial orientation (green) for the hop only (*A*), rotation only (*B*), and hop with coherent rotation (*C*).

**Fig. 4.**
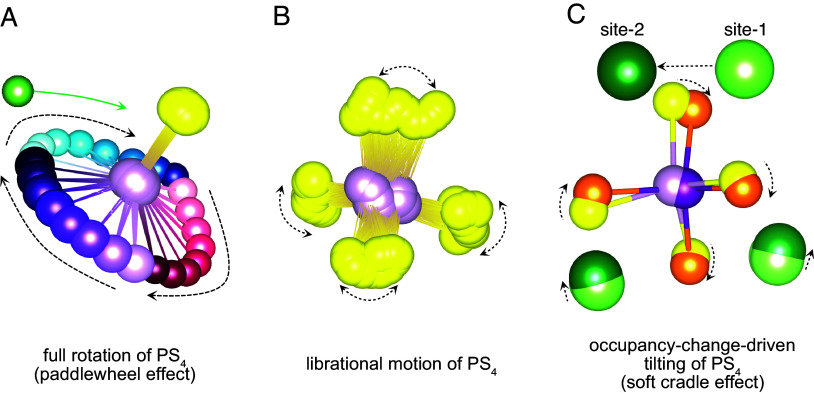
Types of rotational motion of an anion-group. (*A*) Threefold rotation of a PS_4_ unit. The red, blue, and purple atoms represent one of the S^2−^ anion vertices of the PS_4_ unit, and the gradation (direction becoming darker) indicates the rotational trajectory. The yellow sulfur atoms serve as the axis of the threefold rotation of the PS_4_ unit. The green atom represents a lithium-ion that can hop in coherence with the rotating PS_4_ unit. (*B*) Libration of a PS_4_ unit nearby its lowest-energy angular configuration. (*C*) Tilting of PS_4_ groups from the change of nearby lithium occupancies.

To further investigate whether an anion-group rotation occurring in coherence with a lithium-ion hop can reduce the migration barrier of a lithium ion, we compute the energy barrier of three specific events using the nudged elastic band (NEB) method: 1) Li hop only, 2) PS_4_ group rotation only, 3) Li hop with a coherent PS_4_ group rotation. In Fig. 3, we plot the energy along the path for these events in orange and track in green the quaternion distance (°) of the PS_4_ group from the initial orientation. We note that these calculations do not restrict any atomic motion but only differ in the initial and final configuration. In Fig. 3 *B*, *C*, *E*, and *F*, a PS_4_ tetrahedron rotates by 120° around the C_3_ symmetry axis, resembling a paddlewheel, so that the positions of the initial and final structures are permutations of S^2−^ positions near a specific P (Fig. 4*A*). We refer to coherent rotation as the lithium ion undergoing a hop accompanied by its nearest-neighboring PS_4_ group making a rotation in the same direction as to maintain the most optimal bonding environment for the migrating Li ion. These calculations are performed for various paths in both stoichiometric β-Li_3_PS_4_ (*SI Appendix*, Figs. S13 and S14) and β-Li_3_PS_4_ with a lithium-ion vacancy (Fig. 3 and *SI Appendix*, Figs. S15 and S16).

The energy barrier of a lithium-ion hop (*Methods*) without a C_3_ PS_4_ rotation (Fig. 3*A*) is low (0.112 eV) in β-Li_3_PS_4_, consistent with its high ionic conductivity. In contrast, we find that it is extremely challenging for a 120° rotation of the PS_4_ group to occur since we find that E_a_ > 1 eV for all systems (Fig. 3*B* and *SI Appendix*, Figs. S13–S16). This finding is consistent with the event-detection results from AIMD simulations on β-Li_3_PS_4_, where C_3_ rotations are indeed observed (Fig. 1*B*) but occur too infrequently to obtain an activation energy (Fig. 2*F*). The result in Fig. 3*F* shows that even if a lithium ion hops coherently with a PS_4_ rotation around its C_3_ axis, the barrier is much higher than the activation energy of lithium ion diffusion previously reported for β-Li_3_PS_4_ (39, 40) (Fig. 3*C*). The activation energy of the combined rotation and hop (1.308 eV) is lower than the summation of the activation energy of the individual hop (0.112 eV) and rotation (1.594 eV) because of the higher degree of freedom in tracing the minimum energy path of the rotation and hop. Nevertheless, these results show that anion group rotations and the paddlewheel effect are not necessary to create the low energy barrier that activates fast lithium-ion diffusion in β-Li_3_PS_4_. Even without any significant anion group rotation (Fig. 3*A*), Li already moves with low activation energy, consistent with our observation in the AIMD (*SI Appendix*, Table S1 and Fig. S1). We add that while PS_4_ groups may rotate with respect to axes other than those shown in the NEB results of Fig. 3 and *SI Appendix*, Table S2 and Figs. S13–S16, we believe that the event detection results from AIMD (Fig. 2) are exhaustive and serve as direct evidence that the paddlewheel effect in a literal definition does not exist.

### 1.4. Soft-Cradle Effect of Isolated Anion Groups.

To further elucidate any contribution of rotational motion on lithium-ion diffusion, we distinguish three types of rotational motion of anion groups. The first type of rotational motion of anion groups is the rare large-angle rotation, often associated with the paddlewheel effect (Fig. 4*A*). These events have high activation energies, typically from 0.6 eV (HT-Li_2_SO_4_ phase) to 1.5 eV (Li_3_PS_4_ phases). These events are thermally activated and appear as rare large-angle signals in rotation diagrams such as Fig. 1*B*. Our analysis demonstrates that lithium-ion diffusion is not correlated with such rare large-angle rotations as they occur too infrequently and actually do not lower the Li migration barrier in any significant way.

The second type is the librational motion of anion groups (Fig. 4*B*). These are vibration-like back-and-forth oscillations with an amplitude typically within 20° to 30°. These exist for any material, regardless of whether it is a superionic conductor, and they appear as the small-angle background value in the rotation diagram in Fig. 1*B*. The amplitude of librational motion is strongly dependent on the type of anion-group and system and increases slightly with temperature as observed in Fig. 2 and *SI Appendix*, Fig. S2.

The third type of rotational motion is occupancy-change-driven tilting of PS_4_ groups (Fig. 4*C*) which reflects the fact that the PS_4_ orientation can be modified by the nearby presence of Li. This can couple Li site disorder to rotational disorder of the anion groups. As a result, whenever a lithium ion jumps near an anion-group, the orientation of a neighboring anion group will change. Hence, the rotational state of the anion group is “static” until the Li occupancy changes. This occupancy-change-driven tilting of anion groups, which we call the soft-cradle effect, can benefit lithium-ion diffusion because the free tilting of the anion-group allows it to maintain optimal bonding with the migrating lithium ion, potentially lowering the energy of the transition states in the lithium-ion hopping process.

In materials with three-dimensionally connected covalent frameworks, tilting of anion groups with lithium-occupancy change is typically restricted due to geometric constraints imposed by the rigid 3D framework of covalent bonds, resulting in a negligible tilting amplitude. For the soft-cradle effect to be pronounced, the flexibility of the bond between the anion groups is key. In every material for which the paddlewheel effect has been claimed such as amorphous and crystalline Li_3_PS_4_ and HT-Li_2_SO_4_, the anion groups are isolated with only long-range van der Waals interactions connecting them. This is what allows the soft-cradle effect to be prominent in such systems and couples Li-site disorder to anion-group rotational disorder, which is often confused with dynamical disorder (i.e., rotational dynamics of the anion group). Other fast-ion conductors such as Li_10_GeP_2_S_12_ and the argyrodites (41) (Li_6_PS_5_Cl) all share the structural commonality of isolated tetrahedral polyhedra with the absence of covalent bonds between them. Fang and Jena (9) identified a similar mechanism in argyrodite compounds with light monoanion groups (BH_4_^−^, SH^−^) substitutions. They found that responsive dynamics of the anion group relaxation accommodates Li displacement and always lowers the energy barrier of lithium-ion. This idea is conceptually aligned with the proposed soft-cradle effect.

**Fig. 5.**
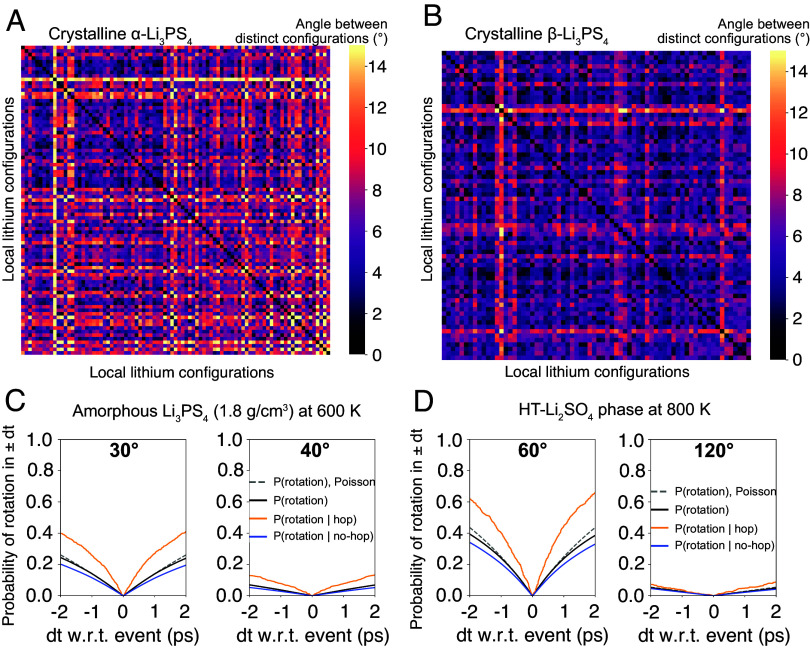
Evidence of soft-cradle effect in crystalline and amorphous Li_3_PS_4_ and HT-Li_2_SO_4_. For crystalline α-Li_3_PS_4_ (*A*) and crystalline β-Li_3_PS_4_ (*B*), each point on the x and y axis represents a distinct local lithium configuration. The matrix element connecting them indicates the angle (quaternion distance) (°) between the PS_4_ orientations of these distinct local lithium configurations. For amorphous Li_3_PS_4_ of density 1.8 g/cm^3^ (*C*) and the HT-Li_2_SO_4_ phase (*D*), the probability of rotation of PS_4_ or SO_4_ groups with various conditions is plotted. The orange lines represent the conditional probability of a rotation event of cutoff angle Θ given that a lithium hop occurs at dt = 0. The blue lines represent the conditional probability of a rotation event of cutoff angle Θ given that no lithium hop occurs within ±2 ps range of dt = 0. The orange and blue conditional probabilities track only the rotation event of the nearest-neighboring anion group to the lithium ions of interest. The black lines represent the probability of a rotation event of cutoff angle Θ regardless of lithium hops. The dashed gray lines indicate the probability of a single anion-group rotation event of a cutoff angle Θ occurring within ± dt assuming that rotation events follow a Poisson process.

To clearly demonstrate the existence of lithium occupancy-change-driven polyhedral tilting, we inspected the PS_4_ orientations in more than 100 unique Li-vacancy configurations of the disordered α- and β-Li_3_PS_4_. For all the possible lithium sites within 4.4 Å of a specific P site, we represent whether each lithium site is occupied or not as an array of Booleans (namely a local occupancy vector). For all P sites of the relaxed enumerated structures that can be mapped to the P(1) site in the primitive cell (provided in *SI Appendix*, *Notes S2 and S3*), we relate the orientation of its PS_4_ tetrahedron to its local occupancy vector, and remove any redundant local occupancy vectors. We compute the quaternion distance (°) between the PS_4_ orientations for all pairs of distinct local-occupancy vectors, presented as a diagonal matrix in Fig. 5 *A* and *B* (details in *Methods*, *SI Appendix*, Figs. S18 and S19 for P(2), P(3), P(4) site in the primitive cell). In α- and β-Li_3_PS_4_, the orientation of the PS_4_ group on average tilts 7.3° and 5.3° as a result of a change in the local-occupancy vector, or simply put, lithium hops. This result clearly demonstrates the prominence of occupancy-change-driven tilting of anion groups in a static manner without the convolution with librational motion. This lithium occupancy-change-driven tilting is also captured in NEB calculations, as shown in Fig. 3*A* and *SI Appendix*, Figs. S13–S16, where the orientation of PS_4_ groups changes up to 10° as a lithium ion migrates.

In systems where anion-group orientations and lithium sites cannot easily be defined such as HT-Li_2_SO_4_ and amorphous Li_3_PS_4_ systems, local occupancy vectors cannot be constructed. Instead, we demonstrate that the soft-cradle effect can be captured from the dynamic trajectory using probability analysis, as presented in Fig. 5 *C* and *D* (see *Methods* for details). We compute the conditional probability that a rotation event of specific angle or larger occurs in spatial and temporal adjacency to a nearby Li-ion hop, denoted as P(rot | hop) (orange line in Fig. 5*C*). We compare this value to the conditional probability that an anion group changes its orientation when no hop occurs in spatial or temporal proximity to the rotation event, denoted as P(rot | no-hop) (blue line in Fig. 5*C*). For amorphous Li_3_PS_4_ of 1.8 g/cm^3^, P(rot | hop) is only significantly higher than P(rot | no-hop) for small-angle rotations between 20° and 40° at 600 K. This result means that rotations in this angle range are more likely to occur when accompanied by a change in occupancy of the nearest-neighboring lithium-ion site, demonstrating the existence of the soft-cradle effect in these materials. This trend is consistently observed for all other amorphous and crystalline Li_3_PS_4_ phases in other temperatures (*SI Appendix*, Figs. S20–S41).

We also compute the probability that an anion group displays a rotation regardless of whether a nearby Li ion makes a hop, P(rot) (black lines in Fig. 5 *C* and *D* and *SI Appendix*, Figs. S20–S41). For all LPS systems where the paddlewheel effect has been claimed, P(rot | hop), P(rot | no-hop), and P(rot) of angle cutoff 40° or larger all converge to 0 as the cutoff angle is increased. This means that large-angle rotational motions are not a necessary condition for lithium hops, contrary to what the paddlewheel effect suggests, as the majority of lithium hops are not spatially or temporally correlated to large-angle rotation motions. Therefore, in amorphous and crystalline Li_3_PS_4_ systems, we can conclusively argue that the paddlewheel effect does not exist and thus is not responsible for their high ionic conductivities. We note that as the rotation events in the dynamic trajectory are inherently a convolution of librational motion and the soft-cradle effect, the range of angles at which P(rot | hop) > P(rot | no-hop) increases with temperature due to the librational amplitude increasing with temperature.

The difference between P(rot | hop) and P(rot | no-hop) (or the soft-cradle effect) is larger in amorphous Li_3_PS_4_ of 2.0 g/cm^3^ (*SI Appendix*, Fig. S33) than 1.8 g/cm^3^ (Fig. 5*C* and *SI Appendix*, Fig. S38) at 600 K. This is because in higher-density amorphous models, lithium ions and PS_4_ groups are interlocked more tightly. As a result, for a lithium ion in higher-density models to hop, it must be accompanied by more pronounced orientational tilting of the nearest PS_4_ group (i.e., the soft-cradle effect). In contrast, as rotations in general are more challenging in higher-density models, as seen in Fig. 2 *C* and *D*, the probability of rotation events of larger angles such as 30° and 40° is significantly lower than that in the lower-density model (black lines in Fig. 5*C* and *SI Appendix*, Figs. S33–S41).

Last, we visit the controversial HT-Li_2_SO_4_ phase where the debate over the existence of the paddlewheel effect began. Contrary to Li_3_PS_4_ systems, we detect significantly more prevalent large-angle rotation events spanning up to 120° in the HT-Li_2_SO_4_ phase (Fig. 2*A*) at 800 K. In addition, the activation energy and event frequency for lithium hops are similar to those of rotation events of cutoff angle 120° (Fig. 2*H* and *SI Appendix*, Fig. S2*F*) throughout all simulated temperature range, which justifies why the paddlewheel mechanism has been historically claimed on this material. However, our conditional-probability analysis reveals that P(rot | hop) for cutoff angle 120° rotations is smaller than 0.1 within a time window of ±2 ps, which means that more than 90% of lithium hops in HT-Li_2_SO_4_ occur without any rotation events larger than 120° in temporal and spatial proximity (Fig. 5*D* and *SI Appendix*, Figs. S42–S45). Instead, we observe that P(rot | hop) is higher than P(rot | no-hop) up to angles as large as 120°, indicating that a strong soft-cradle effect exists in this system. Therefore, we conclude that the paddlewheel effect does not exist in HT-Li_2_SO_4_ and instead the soft-cradle effect is responsible for the high ionic conductivity of the HT-Li_2_SO_4_ phase.

## 2. Discussion

Because of the challenge in directly visualizing atomic motion in materials, evidence for specific correlation between Li motion and dynamic structural features tends to be indirect. Ab initio simulations offer a direct mechanistic insight into the correlated nature of dynamical events, in particular when those events occur on the fast time scale which molecular dynamics can sample. We find that in various materials for which the paddlewheel effect has been claimed, the energy barrier associated with large angle rotations is much larger than that for Li motion and inconsistent with the activation energy experimentally measured for Li diffusion. We furthermore find that the frequency at which large-angle rotations occur is orders of magnitude below that of Li hops at room temperature (Fig. 2*B*) providing conclusive evidence that fast Li-ion conduction is not related to anion group rotational dynamics. Rather, we find a static correlation between Li occupancy of sites and small tilts of anion-group, thereby correlating the Li site disorder which is often present in good Li-ion conductors (5, 42) with anion group rotational disorder. This anion-group tilting is possible because of the lack of covalent connectivity between the anion groups in many of the materials investigated.

The rotational motion of anion groups and the translational motion of lithium ions both have various time scales contributing to them. Lithium ions vibrate at their local energy minima and stochastically overcome energy barriers to hop to other local energy minima. The time scale between these large and small displacements is usually well separated. Even for a barrier of 200 meV, a hop only occurs every 2,300 attempts (or vibrations) at 300 K. Similarly, anion groups librate in the rotational coordinates around their local energy minima, and stochastically overcome energy barriers to make a permanent large-angle rotation to another local energy minima orientation. The rare stochastic coordinate changes (Li hops, large-angle rotations) can be thought of moving the system from one energy well in phase space to another. Dynamic disorder can be defined here as the vibrational or librational range of motion with respect to a given energy basin, whereas static disorder corresponds to an ensemble of multiple energy basin sites (43–45). As pointed out by Zhang and Nazar (23), the static disorder should be visible in a maximum entropy method from neutron diffraction at very low temperature where the vibrations and librations become negligible. Our work demonstrates that the dynamic disorder of anion group orientations is not directly correlated to lithium-ion diffusion. This can be seen from the comparable frequency and amplitude of librational motion (10 to 30°) between the fast and slow Li-conducting polymorphs of Li_3_PS_4_. Even for the HT-Li_2_SO_4_ phase with significant dynamic disorder seen from the large-amplitude librational motion, more than 90% of lithium-ion hops do not occur together with such dynamic motion.

The soft cradle effect, which modifies the anion group orientation depending on the position of nearby Li, suggests that in fast Li-ion conductors with weak binding between isolated anion groups (which is the case for previously paddlewheel-claimed materials), there will be correlations between the static disorder of lithium-ion site occupancy and the static disorder of anion group orientations. This concept suggests that the existence of Li-vacancy disorder can be a cause of the static disorder of anion group orientations. Such an effect is especially noticeable when the interlocking between anion groups is soft.

We emphasize the importance of distinguishing dynamic disorder from the static disorder in the orientation of anion groups caused by the local disorder of lithium. Based on our analysis of the crystalline and amorphous Li_3_PS_4_ systems, the frequency of large-angle anion group dynamics is orders of magnitude lower than that of the Li motion. The only prevalent dynamic disorder is the librational motion of 20 to 40°. Instead, we find that in the fast thiophosphate conductors, a soft-cradle effect occurs, whereby the lithium-ion achieves optimal anion coordination, resulting in a static disorder in the orientation of the anion groups. This is the key origin of the diffuse sulfur ligand nucleus distribution observed in β-Li_3_PS_4_ (25).

The framework in which we understand the effect of anion-group rotational motion on lithium-ion diffusion can provide an overarching interpretation of the experimental and computational evidence that has previously been taken to support the paddlewheel effect. In amorphous Li_3_PS_4_, Smith and Siegel (22) identified several examples from MD simulations where lithium-ion migration occurs together with large, quasi-permanent reorientations of PS_4_^3−^ anions up to 50°. They referred to this as paddlewheel effect by using the term in a broad sense (i.e. existence of any correlation between anion group rotation and lithium-ion hops). In fact, conceptually their observation is similar to the soft-cradle effect. While we do observe such events [nonzero probability of P(rot | hop)], our work clarifies that the majority of Li-ion hops in amorphous Li_3_PS_4_ occur without any 30 to 50° rotations in temporal or spatial proximity, even at elevated temperature (600 K, Fig. 5*C* and *SI Appendix*, Figs. S33 and S38). Our complete statistical analysis reveals that while large-angle rotations do occur at high temperature (>800 K), they are rare, with the divergence between the frequency of large-angle rotations and lithium-ion hops becoming larger at 300 K due to the high activation energy of large-angle rotations. In the 20 to 40° range, libration events occur with high frequency at all temperatures (Fig. 2 *A* and *B* and *SI Appendix*, Fig. S2). Therefore, as different types of rotational events occur with very different characteristic frequencies and activation energies, we emphasize the advantage of detecting each type of anion-group reorientations events and computing their corresponding activation energies. As previous work (22) did not differentiate various types of anion-group reorientational motions, we expect that the PS_4_ reorientation barrier measured (0.27 eV) originated mostly from librational motion, not a literal paddlewheel-like motion. We add that the very low amorphous model density (1.56 g/cm^3^) in simulations by Smith (22) et al. may have led to unphysically large-amplitude librational motion as compared to our amorphous models with densities of 1.8 and 2.0 g/cm^3^, which serves as a lower and upper bound of the amorphous samples with minimized porosity (46) (1.88 g/cm^3^).

Our analysis rationalizes the polymorph dependence of lithium ionic conductivity among the Li_3_PS_4_ phases. It has previously been claimed that the β-phase is a faster conductor than the γ-phase because of its paddlewheel effect (25). In support of the argument, the maximum entropy method on neutron diffraction data was shown to have a more diffuse PS_4_ nucleus distribution in the β-phase. Because the nucleus probability distributions obtained from neutron diffraction experiments are averaged over the scattering trajectories through the bulk sample as well as the beam exposure time, they provide only time- and space-averaged data and cannot distinguish static disorder of anion-group orientations from dynamical disorder. Our analysis shows that all the polymorphs have comparable rotational frequencies as well as activation energies (Fig. 2 *E*–*G* and *SI Appendix*, Fig. S2 *C–E*), indicating that rotational motion is not the governing factor of ionic conductivity among these polymorphs. Rather, the unique PS_4_ packing in the crystal structure of β and α-Li_3_PS_4_ inherently provides low-energy lithium-migration pathways, allowing them to be faster lithium-ion conductors compared to the γ-phase. Indeed, it has been previously shown that the anion sublattices of β and α-Li_3_PS_4_ closely resemble a body-centered cubic (BCC) framework, which should enable more facile lithium-diffusion pathways as compared to the hexagonal close-packed (HCP) anion framework of the γ-phase (2, 47). As lithium-ion hops are more frequent, PS_4_ groups accordingly tilt more frequently via the soft-cradle effect, which results in a more diffuse nucleus distribution in MEM experiments than for the γ-phase. Therefore, we argue that rotational motions are not responsible for faster diffusion in α- and β-Li_3_PS_4_ but rather occur as a result of their flat Li-energy landscape in the form of occupancy-change-driven tilting of PS_4_ groups.

A common technique to argue for anion-group-assisted fast Li-ion diffusion is to compare the Li migration energy with and without freezing the anion group degrees of freedom (22, 29, 34, 48). We note first of all that, by definition, the freezing of any degree of freedom in a simulation will raise the migration energy for Li hopping as that frozen degree of freedom is not available to lower the energy along the migration trajectory. But more importantly, if the paddlewheel effect were the major underlying mechanism for lithium-ion diffusion in such systems, freezing anion groups would turn these systems into terrible conductors in a dramatic fashion. However, the freezing of such librational and translational motion of anion groups in simulations tends to only slightly raise the migration barriers (22, 29, 34, 48). The lower degree of freedom of ionic motions in constrained simulations results in lithium ions following slightly higher-energy pathways to make the same hop because the anion groups cannot tilt to make way for lithium ions. Therefore, the slightly lower conductivity in constrained MD simulations is evidence of the nonexistence of the paddlewheel effect and the existence of the soft-cradle effect.

As the term paddlewheel effect implies a unidirectional momentum transfer from an anion-group to a lithium ion, it is crucial to understand whether rotation events precede or follow the hop of an adjacent lithium ion. In Fig. 5 *C* and *D* and *SI Appendix*, Figs. S20–S45, P(rot | hop) is temporally symmetric with respect to dt = 0 (the moment of a hop event), indicating that rotation events neither follow nor precede a hop. As such, rotation events in lithium superionic conductors do not involve a unidirectional momentum transfer process resembling a literal paddlewheel. Instead, when understanding the soft-cradle effect, the lithium-ion hop and the tilting of anion groups occur simultaneously, associated within soft anharmonic phonon modes, as suggested in previous work (34, 49).

Various terms have been used to describe the relationship between anion-group rotation and lithium-ion diffusion, such as the paddlewheel effect (23), cogwheel effect (13, 14), and revolving door mechanism (12). The commonality is that these terms 1) imply that the rotational motion relevant in faster lithium-ion diffusion is the rare large-angle rotations and 2) that the lithium-ion migration is driven by a unidirectional momentum transfer from nearby anion groups, both of which we prove to be untrue. Regardless of whether these terms are used in a literal sense or as an umbrella term, we believe that these terminologies imply a physically imprecise picture for the relationship between rotational motion and lithium-ion diffusion in the paddlewheel-effect-claimed materials.

We emphasize that when analyzing the correlation between lithium-ion hops and anion-group rotation, it is crucial to employ individual event-detection algorithms and apply rigorous statistical methods. Previous computational work used time- and space-averaged information such as rotational diffusivity values (rad^2^/s) from the Green–Kubo formula (22), angular correlation functions (24, 25), and a Helmholtz free energy map (24, 25, 35). Similarly, experimental methods such as a MEM analysis on neutron diffraction data used to demonstrate the reorientational motions from the diffuse nuclear probability density of anion groups (24, 25), QENS (32, 35, 50) that were used to identify, or spin lattice relaxation NMR experiments (41) to measure rotational jump rates, only provide spatially and temporally averaged information. Therefore, all of those techniques confound distinct types of rotational motions existing in these materials and cannot identify a precise mechanistic picture. For example, the diffuse ligand nucleus density in MEM maps can result from both dynamic rotational disorder of anion groups as well as static disorder of distinct anion group orientations. Our quaternion-based algorithm to track individual PS_4_ orientation throughout the entire trajectory allows us to accurately detect each rotational event and understand whether each type of rotational motion is spatially and temporally related to lithium-ion hops, allowing us to reveal that no paddlewheel effect for enhancing Li diffusion exists.

Our quaternion method of detecting rotational motion of anion groups is more rigorous than the previously used scheme of tracking the angle between an anion-group bond (e.g., P–S) and a fixed axis. This is because a projected rotation method fails to detect rotations with respect to the fixed axis. In addition, when detecting a rotation event, the aggregate angle that the entire anion group rotated is not the same as the change in the projected angle of each ligand, and rather it must be calculated backward from the four projected angles of the ligands. In contrast, by treating anion groups as rigid bodies and using quaternion expressions, any rotational motion regardless of the rotation axis can be detected using a single physical quantity. *SI Appendix*, Fig. S46 illustrates the small degree of distortion of anion groups even at high temperature, indicating that such a rigid-body assumption of anion groups holds true in our simulations.

## 3. Conclusion

We developed a quaternion-based rotational event-detection algorithm and applied rigorous statistical methods to investigate possible correlation between lithium-ion hops and anion-group rotations. Contrary to what the paddlewheel effect suggests, large-angle rotations occur orders of magnitude less frequently than lithium-ion hops and have much higher activation energies, suggesting that the paddlewheel effect in a literal sense does not exist and plays no role in accelerating the lithium-ion diffusion of these materials. Instead, we identify that lithium-site occupancy-change-driven tilting of anion groups occurs in temporal and spatial correlation with lithium-ion hops. We argue that this type of rotational motion benefits lithium-ion diffusion by providing optimal cradle-like bonding throughout the transition states, namely, the soft-cradle effect. We hope that this work can finally end the debate on the identity and existence of the paddlewheel effect in fast lithium-ion conductors. Our analysis may serve as a guideline for the theoretical understanding of the spatial and temporal correlation between lithium-ion hops and anion-group rotations as well as laying an important foundation for understanding superionic conductivity in technologically important materials.

## 4. Methods

### 4.1. Quaternion Algorithms for Representation of Rotations.

We implemented quaternion-based rotation-detection algorithms in this work. This analysis is based on Euler’s rotation theorem, which states that any motion of a rigid body without translational motion is equivalent to a single rotation with respect to some axis. The quaternion computations were performed using the PyQuaternion (51) package. For each anion group, we recorded the rotation matrix required to reach the orientation at snapshot t from the first snapshot of the simulation using singular value decomposition. This step effectively removes the vibrational distortions away from a perfect polyhedron and extracts only the rotational motion of the anion group as a rigid body, which we show in *SI Appendix*, Fig. S46 to be a valid assumption. This rotation matrix was converted to a unit quaternion and was saved throughout the entire trajectory. We then computed the quaternion distance between Q(t_0_) and Q(t_0_ + dt) for t_0_ values spanning the entire simulation and dt value up to 5 ps to generate the rotation diagram. The quaternion distance between q_1_ and q_2_ corresponds to the magnitude of the rotation angle to align q_1_ to q_2_ and can be computed as the geodesic connecting them on the 4-dimensional unit hypersphere (52). It is computed by the equation: $d\left(q_{1},\;q_{2}\right)=\text{min}\left(2\text{log}\left(q_{1}q_{2}^{T}\right),\;2\log(-q_{1}q_{2}^{T})\right)$. The minimum operation is used because q and −q encode the same rotation. This algorithm is based on the fact that the rotation operation to align the orientation of two anion groups facing different directions (q_1_ and q_2_) can be expressed as a single rotation angle with respect to some axis. All of the analysis tools developed in this work, including rotational event detection algorithms using quaternions, hop detection algorithms, conditional probability analysis are publicly provided in CorrelationAnalyzer python package (*SI Appendix*, *Note S4*).

### 4.2. Event-Detection Algorithms.

After constructing rotation and translation diagrams, events were detected by drawing a horizontal line at dt = 1 ps. This value of dt was selected as it is in the range of a typical phonon frequency (42, 53). Selecting too small of a value of dt results in an underestimation of the “angle” or “distance” of rotation or hop events, respectively, whereas too large of a value of dt results in a lower precision of event detection as multiple events can be convoluted within a large dt value. Subsequently, we detected peaks with peak heights larger than the angle or distance cutoff values and peak prominence larger than 50% of the angle or distance cutoff values. The actual time t_0_ of the event, height of the peak (angle or distance), and the atom index of the event were recorded. These analyses were performed for a variety of angle or distance cutoff values. Hop distance cutoff value of 3.0 Å was used for all analysis shown in the main text. The error bars of the event frequencies of rotations and hops were computed assuming a Poisson distribution (see gray lines in Fig. 5 *C* and *D*).

### 4.3. Ab Initio Molecular Dynamics Simulations.

AIMD simulations were conducted using the Vienna ab initio simulation package (54) (VASP) based on non-spin-polarized density functional theory (DFT) calculations with the projector augmented wave method (55). The generalized gradient approximation (56) was chosen as the exchange-correlation functional. We set the kinetic-energy cutoff of the plane-wave basis to 300 eV and 520 eV for Li_3_PS_4_ and Li_2_SO_4_ phases, respectively. Г-only k-points were used for the AIMD simulations. The NVT ensemble was simulated with a Nosé–Hoover thermostat and 2-fs time step. The amorphous Li_3_PS_4_ structure was generated by first selecting the lowest electrostatic energy configuration among 100 structures with randomly placed 16 P^5+^ atoms, then generating PS_4_^3−^ anions based on the positions of P^5+^ ions and placing 48 Li^+^ cations in the void space, and finally relaxing the structure using a 20-ps AIMD at 1,000 K. The volume of the simulation cell was fixed in a cubic lattice to reproduce the experimentally reported densities, 1.8 and 2.0 g/cm^3^ (46, 57, 58). For α-, β-, and γ-Li_3_PS_4_, supercell sizes of (2 × 2 × 2), (1 × 2 × 2), and (2 × 2 × 2) with respective to their primitive cells were used respectively, resulting in 16 P atoms per supercell. The lattice parameters of the crystalline supercells were fully relaxed. For the HT-Li_2_SO_4_ phase, the SO_4_ sublattice reported by Forland and Krogh-Moe (59) was used and lithium ions were inserted at the positions refined by Nilsson et al. (60) The position of atoms in the structure were randomly perturbed by 0.1 Å while fixing the experimentally known lattice parameters. A 2 × 2 × 2 supercell of this structure was used for the AIMD simulations.

### 4.4. Minimum Energy Pathways of Rotations and Hops.

NEB calculations were performed in a (1 × 2 × 2) and (2 × 2 × 2) supercell of β-Li_3_PS_4_ and HT-Li_2_SO_4_, respectively in the same structures used to initialize the AIMD simulations. For β-Li_3_PS_4_, the lithium positions were ordered to full 8d and 4b occupancy following the work by Lepley et al. (61) based on the X-ray diffraction refinement by Kanno et al. (62). NEB calculations in the stoichiometric β-Li_3_PS_4_ were computed for the migration pathway of an 8d lithium in the ground-state structure to an adjacent 4c site (formation of an 8d-vacancy and 4c interstitial pair, named path-S1, *SI Appendix*, Fig. S13) and the migration pathway of an 8d lithium to a vacant 8d site while maintaining the 4c interstitial (path-S2, *SI Appendix*, Fig. S14). NEB calculations of β-Li_3_PS_4_ with a single vacancy were computed for a vacancy migration of 8d sites while maintaining a common edge with an adjacent PS_4_ group (path-V1, *SI Appendix*, Fig. S15), vacancy migration between 4b sites while maintaining a common edge with an adjacent PS_4_ group (path-V2, *SI Appendix*, Fig. S16), and vacancy migration between 8d sites while maintaining a common vertex with an adjacent PS_4_ group (path-V3, Fig. 3). For each distinct lithium-migration pathway, we computed the 120° (C_3_) rotation barrier of the common nearest-neighboring PS_4_ group of the migrating lithium, as well as the lithium-migration barrier accompanied by a coherent C_3_ rotation of the nearest-neighboring PS_4_ group. A uniform background charge was applied to retain the oxidation states of remaining ions when performing vacancy NEB calculations, following a previous systematic investigation on the usage of background charge (63). The supercell with a vacancy was fixed to the lattice parameter of the stoichiometric supercell. The force between images was converged to 0.01 eV/Å.

### 4.5. Generation of Local Occupancy Vectors.

We enumerated Li/vacancy orderings in the disordered α and β-Li_3_PS_4_ structures based on previous neutron diffraction refinements (47). Enumerations were performed using Pymatgen (64) on diagonal supercells up to size 4 (1 × 1 × 1, 2 × 2 × 1, 2 × 1 × 2, 1 × 2 × 1, 2 × 1 × 1, 1 × 1 × 2, 1 × 2 × 2), and the structures with the lowest Ewald electrostatic energy were selected. The low-energy structures were also enumerated from Monte Carlo simulations based on cluster expansions on each phase using SMOL (65); more details of the cluster expansion are provided in other work (66). Ionic relaxations were performed on each structure while fixing the supercell lattice parameters, using the PBE functional (56) and PAW potentials (67), as implemented in VASP. The structures were converged to 10^−5^ eV in energy and 0.01 eV/Å in the forces. When generating the local occupancy vectors, only the data from the symmetrically distinct structures were extracted and shown. High energy structures with energy higher than 1.4 eV/primitive cell referenced to the lowest energy enumerated structure were excluded in this analysis. Lithium local occupancy vectors were built by mapping to the Li sites identified by the neutron diffraction refinement (47), as listed in *SI Appendix*, *Notes S2 and S3*.

### 4.6. Probability Analysis of Events.

Probability analysis was performed after detecting every rotation event with various angle thresholds and hop events of a distance threshold (3.0 Å). To compute P(rot | hop), for each lithium in the structure, we detected all of the hop events that occurred in the trajectory and identified all of the rotation events of the migrating lithium’s first-neighboring PS_4_ group within ±2 ps of the moment of the hop. Referring to the moment of the lithium hop as dt = 0, we identified the first rotation following the hop (dt = t_right_ > 0) and the last rotation prior *to* the hop (dt = t_left_ < 0). For each lithium-hop event, a summed Heaviside step function H(dt − t_right_) + (1 − H(dt − t_left_)) was constructed to record the time it took for a rotation to follow or precede a hopping event. Finally, for all the lithium hops in the given trajectory, these summed Heaviside step functions were averaged to result in the conditional probability of rotation assuming a hop event occurred at dt = 0 [i.e., P(rot | hop)].

To compute P(rot | no-hop) for a given rotation angle θ, we considered each lithium ion in the simulation cell and selected snapshots where that Li ion did not make a hop for at least ±2 ps. We then iterated through these no-hop snapshots and identified all of the rotation events of its first-neighboring anion groups that were within 2 ps of each snapshot. Referring to the moment of each no-hop snapshot as dt = 0, we identified the first rotation following the hop (dt = t_right_ > 0) and the last rotation prior to the hop (dt = t_left_ < 0). For each no-hop snapshot, a summed Heaviside step function H(dt − t_right_) + (1 − H(dt − t_left_)) was constructed to record the time it took for a rotation to occur. These summed Heaviside step functions were averaged for all the no-hop snapshots of all lithium ions in the simulation cell to result in the conditional probability of rotation (θ) assuming that no hops occur in temporal and spatial proximity.

To compute P(rot) for a given rotation angle θ, we iterated through all snapshots and identified for each lithium ion in the cell all of the rotation events of its first-neighboring anion groups that were within 2 ps of each snapshot. Similar summed Heaviside step functions were created and were averaged for all the snapshots of all lithium ions throughout the entire trajectory to result in the probability of rotation (θ) with no hop-related conditions. When computing P(rot)_Poisson_, we assumed each rotation event as a Poisson process, which means that the occurrence of an event is independent of its previous event. The Poisson probability to observe x events within dt assuming an event frequency f is expressed as Poisson(x in dt; f). Using the event frequencies of each rotation angle measured from the AIMD trajectories, P(rot)_Poisson_ was computed as 1 − Poisson(x = 0 in dt; f_measured_) for dt values within ±2 ps.

## Supplementary Material

Appendix 01 (PDF)

Dataset S01 (XLSX)

Dataset S02 (XLSX)

## Data Availability

Codes used to perform all of the analysis are publicly available in a python package named CorrelationAnalyzer. All other data are included in the manuscript and/or supporting information.
