# Supplementary material for: The nonexistence of a paddlewheel effect in superionic conductors
Source: Proc Natl Acad Sci U S A. 2024 Apr 24;121(18):e2316493121. doi: 10.1073/pnas.2316493121 (PMC11067015; doi:10.1073/pnas.2316493121)
Supplement: Supplementary file 1 — Appendix 01 (PDF) [file pnas.2316493121.sapp.pdf]

Supplementary Information for

The nonexistence of a paddlewheel effect in superionic conductors

KyuJung Jun[1,2,†], Byungju Lee[2,3,†], Ronald Kam[1,2], Gerbrand Ceder[1,2,*]

1. Department of Materials Science and Engineering, University of California, Berkeley, Berkeley, CA 94720, USA
2. Materials Sciences Division, Lawrence Berkeley National Laboratory, Berkeley, CA 94720, USA
3. Computational Science Research Center, Korea Institute of Science and Technology (KIST), Seoul 02792, Republic of Korea

† These authors contributed equally.

* Correspondence: gceder@berkeley.edu

Note S1. Details on the hop and rotation event detections on the AIMD trajectories

Dataset S1.xlsx file provides the complete event detection results at all of the simulated temperatures of our model systems for various hop distance and rotation angle cutoffs. The standard deviations of the frequencies are computed by assuming Poisson statistics of each type of event.

Dataset S2.xlsx file provides the activation energy, error of activation energy, 300 K extrapolated event frequency as well as its upper and lower bound computed for all of our model systems for various hop distance and rotation angle cutoffs.

Table S1. Diffusion coefficients, relative error of diffusion coefficients, activation energies of diffusion, error of activation energies and 300 K-extrapolated ionic conductivity with lower and upper bounds.

	T (K)	D (cm^2/s)	$D_{relative\ error}$	Ea (eV)	Ea_{error} (eV)	$\sigma_{300\ K}$ (mS/cm)
α-Li_3PS_4	600	1.55E-05	0.138	0.220	0.016	**21.7** (11.0, 42.8)
	650	1.78E-05	0.135			
	700	2.27E-05	0.129			
	750	3.17E-05	0.101			
	800	4.27E-05	0.098			
	900	5.39E-05	0.090			
	1000	7.81E-05	0.083			
β-Li_3PS_4	500	1.07E-06	0.410	0.317	0.017	**1.66** (0.808, 3.41)
	600	6.11E-06	0.191			
	650	1.11E-05	0.168			
	700	2.02E-05	0.121			
	750	2.44E-05	0.119			
	800	3.10E-05	0.111			
	900	5.30E-05	0.096			
	1000	7.72E-05	0.085			
γ-Li_3PS_4	600	2.39E-07	0.811	0.998	0.084	**1.43E-07** (4.39E-09, 4.69E-06)
	650	7.12E-07	0.520			
	700	4.59E-06	0.216			
	750	1.76E-05	0.134			
	800	3.46E-05	0.103			
Amorphous Li_3PS_4 (2.0 g/cm^3)	600	3.52E-06	0.175	0.316	0.022	**1.02** (0.419, 2.49)
	700	1.02E-05	0.123			
	800	1.98E-05	0.104			
	900	2.41E-05	0.116			
	1000	4.83E-05	0.093			
Amorphous Li_3PS_4 (1.8 g/cm^3)	600	6.82E-06	0.171	0.347	0.024	**0.986** (0.365, 2.66)
	650	9.67E-06	0.156			
	700	1.86E-05	0.124			
	750	3.55E-05	0.104			
	800	4.22E-05	0.092			
	900	6.04E-05	0.088			
High-temperature Li_2SO_4	800	6.19E-06	0.220	0.457	0.043	**0.0235** (0.00405, 0.136)
	850	1.80E-05	0.155			
	900	2.33E-05	0.129			
	950	3.29E-05	0.116			
	1000	3.93E-05	0.099			
	1100	5.91E-05	0.098			

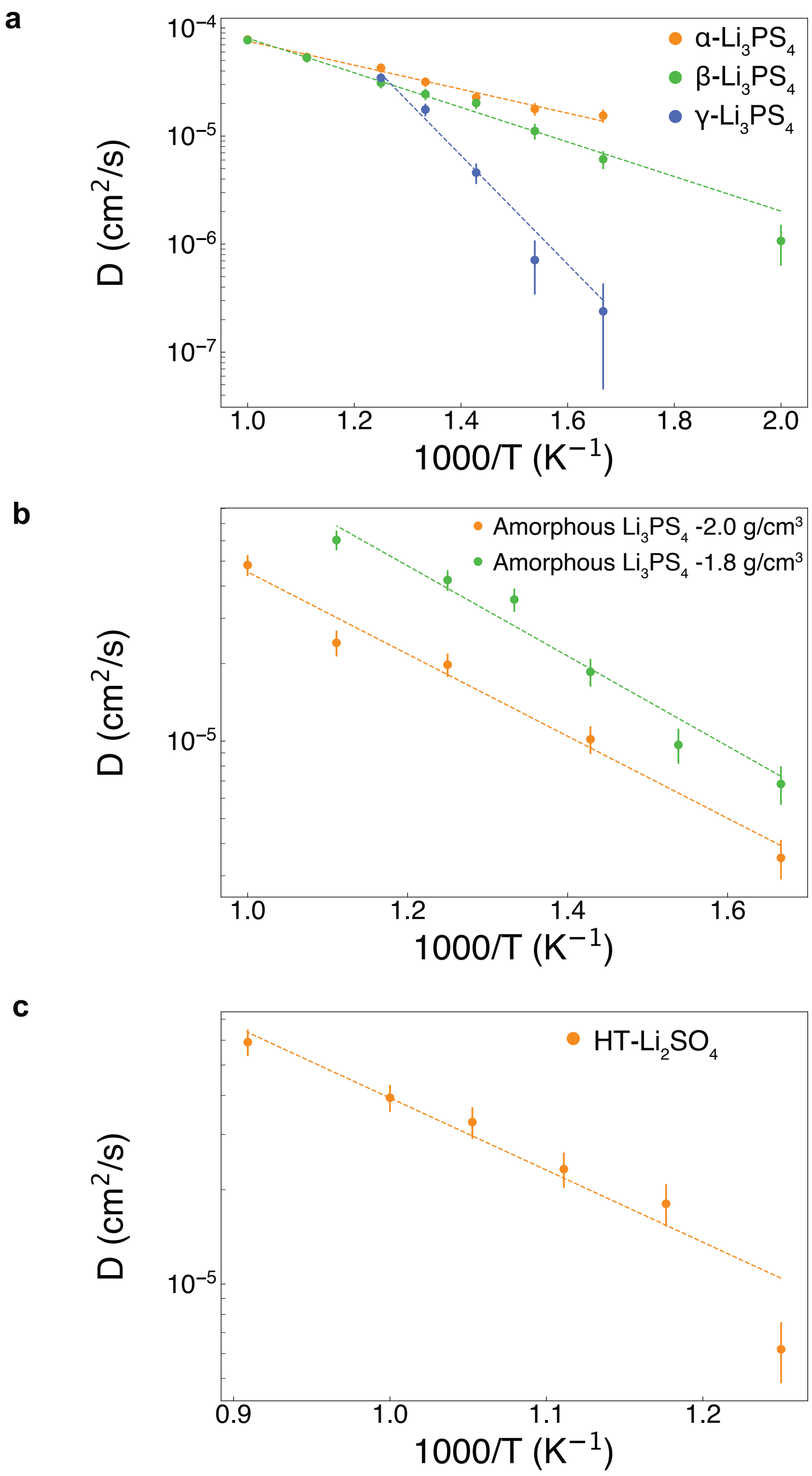

Figure S1. Arrhenius plots of diffusion coefficients for (a) crystalline Li_3PS_4 systems, (b) amorphous Li_3PS_4 systems and (c) high-temperature Li_2SO_4 phase.

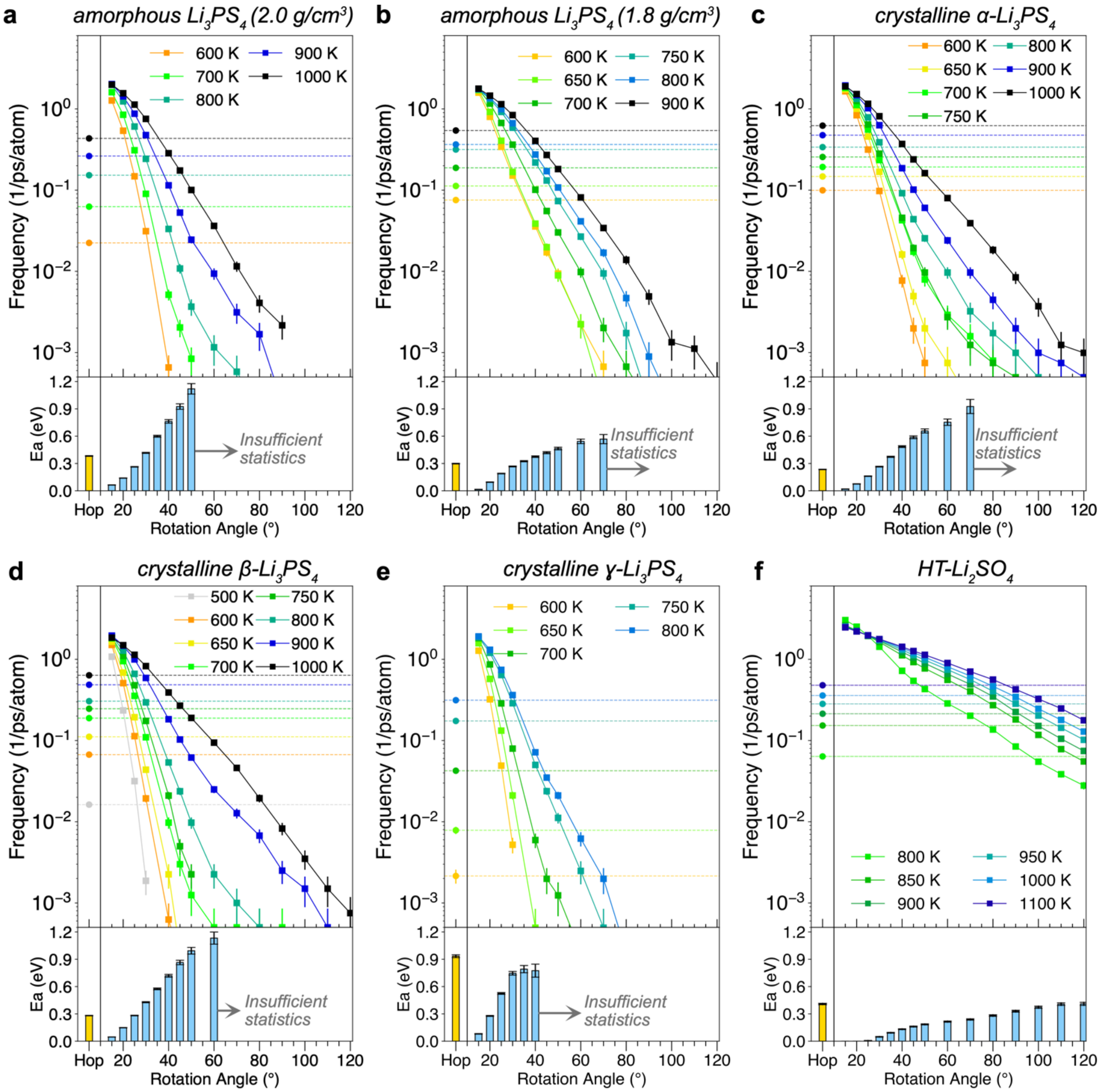

Figure S2. Anion-group rotation and lithium-ion hop frequency for each system. (a) amorphous Li_3PS_4 of density 2.0 g/cm^3, (b) amorphous Li_3PS_4 of density 1.8 g/cm^3, (c) crystalline α-Li_3PS_4, (d) crystalline β-Li_3PS_4, (e) crystalline Ɣ-Li_3PS_4, and (f) HT-Li_2SO_4 phase. The squares on the upper part of each subfigure indicate the frequency of the rotation event of each angle at various temperatures, and the horizontal dashed lines depict that of Li hopping events. The circles represent the hop event with a cutoff distance of 3 Å at various temperatures. The lower subfigure shows the activation energy of hop events (orange) as well as of rotation events of each angle. The activation energies of large rotation angles are not plotted if there were insufficient number of rotational events to compute the activation energy. The hop frequencies and rotation frequencies are normalized by the number of lithium-ion and anion-groups, respectively.

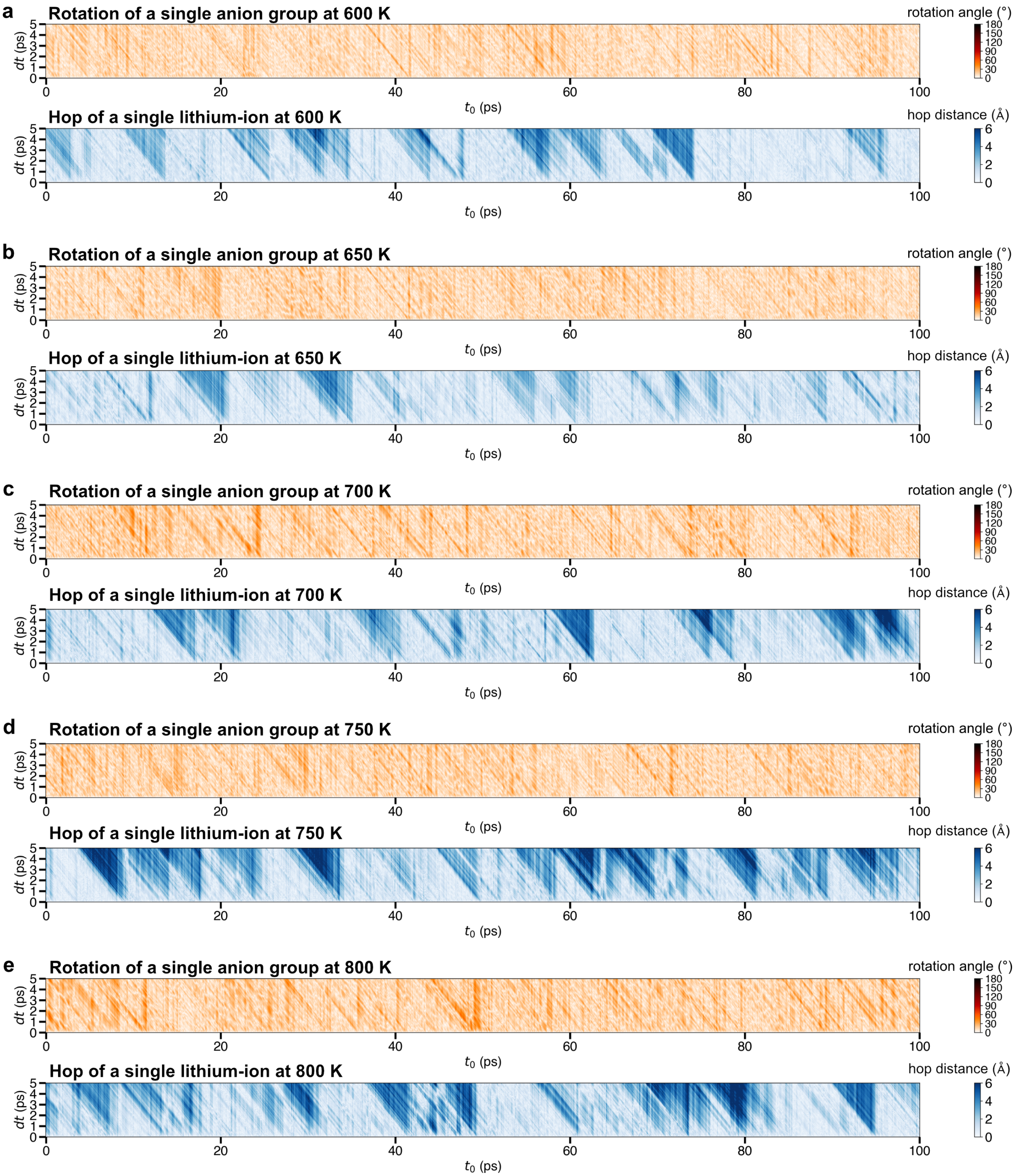

Figure S3. Exemplary 100-ps-long rotation and translation diagram that tracks a single anion group and a single lithium-ion in α-Li_3PS_4 computed at 600 K (a), 650 K (b), 700 K (c), 750 K (d), 800 K (e). Note that the specified anion group and lithium-ion are not necessarily spatially close to each other.

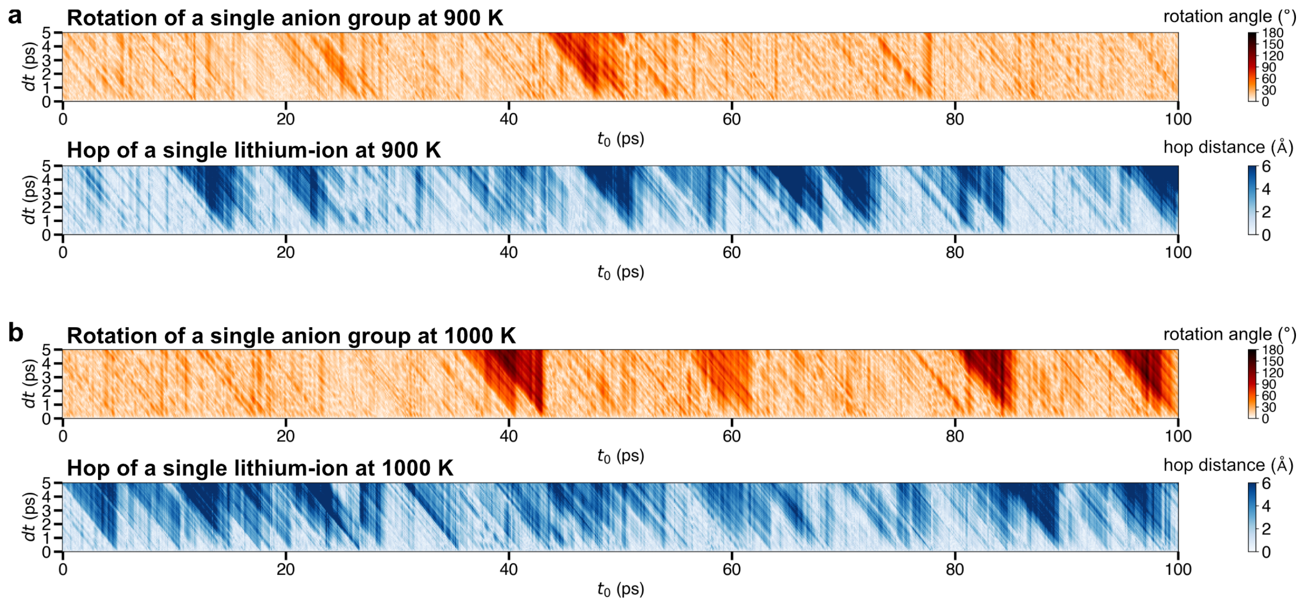

Figure S4. Exemplary 100-ps-long rotation and translation diagram that tracks a single anion group and a single lithium-ion in α-Li_3PS_4 computed at 900 K (a), 1000 K (b). Note that the specified anion group and lithium-ion are not necessarily spatially close to each other.

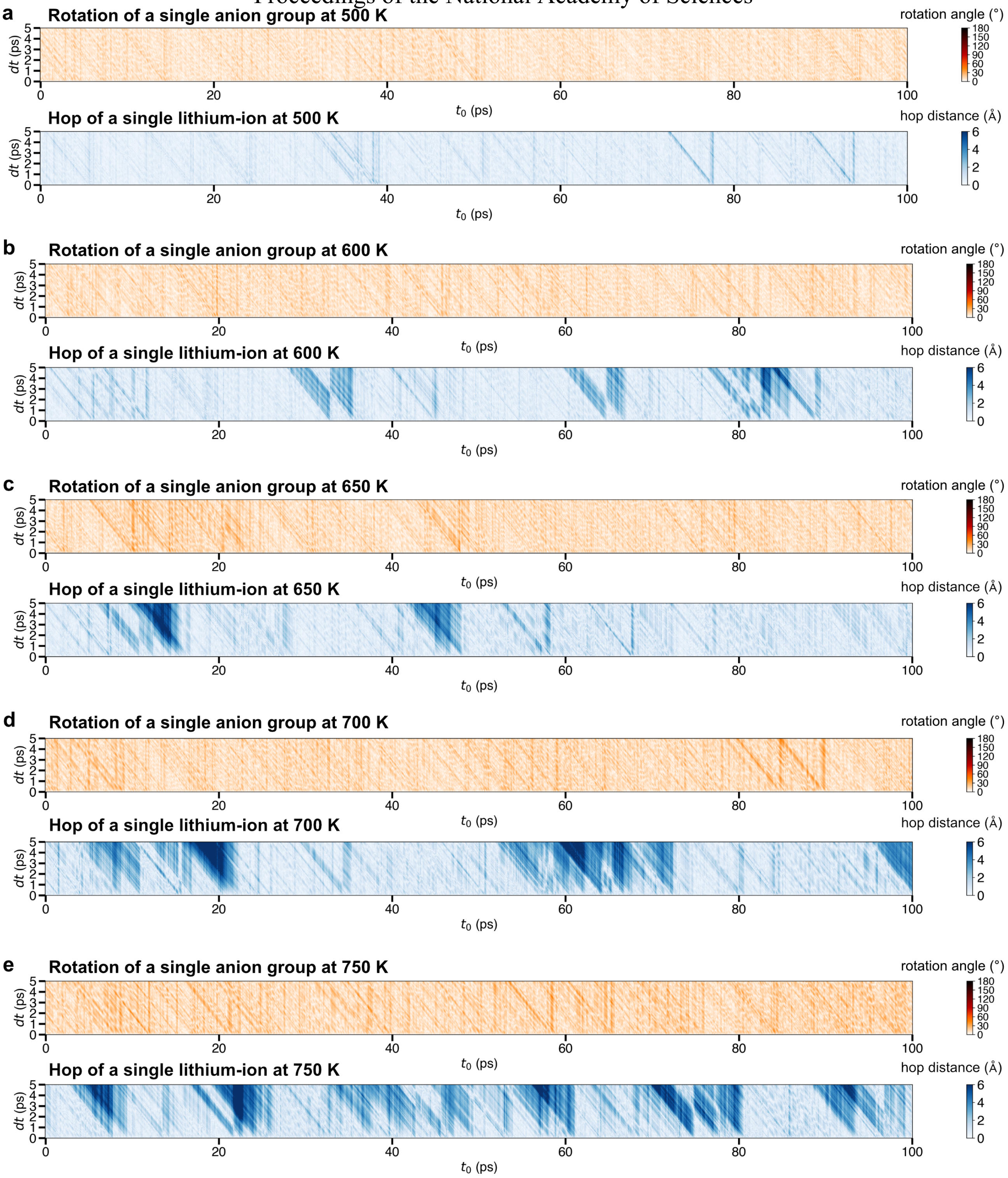

Figure S5. Exemplary 100-ps-long rotation and translation diagram that tracks a single anion group and a single lithium-ion in β-Li_3PS_4 computed at 500 K (a), 600 K (b), 650 K (c), 700 K (d), 750 K (e). Note that the specified anion group and lithium-ion are not necessarily spatially close to each other.

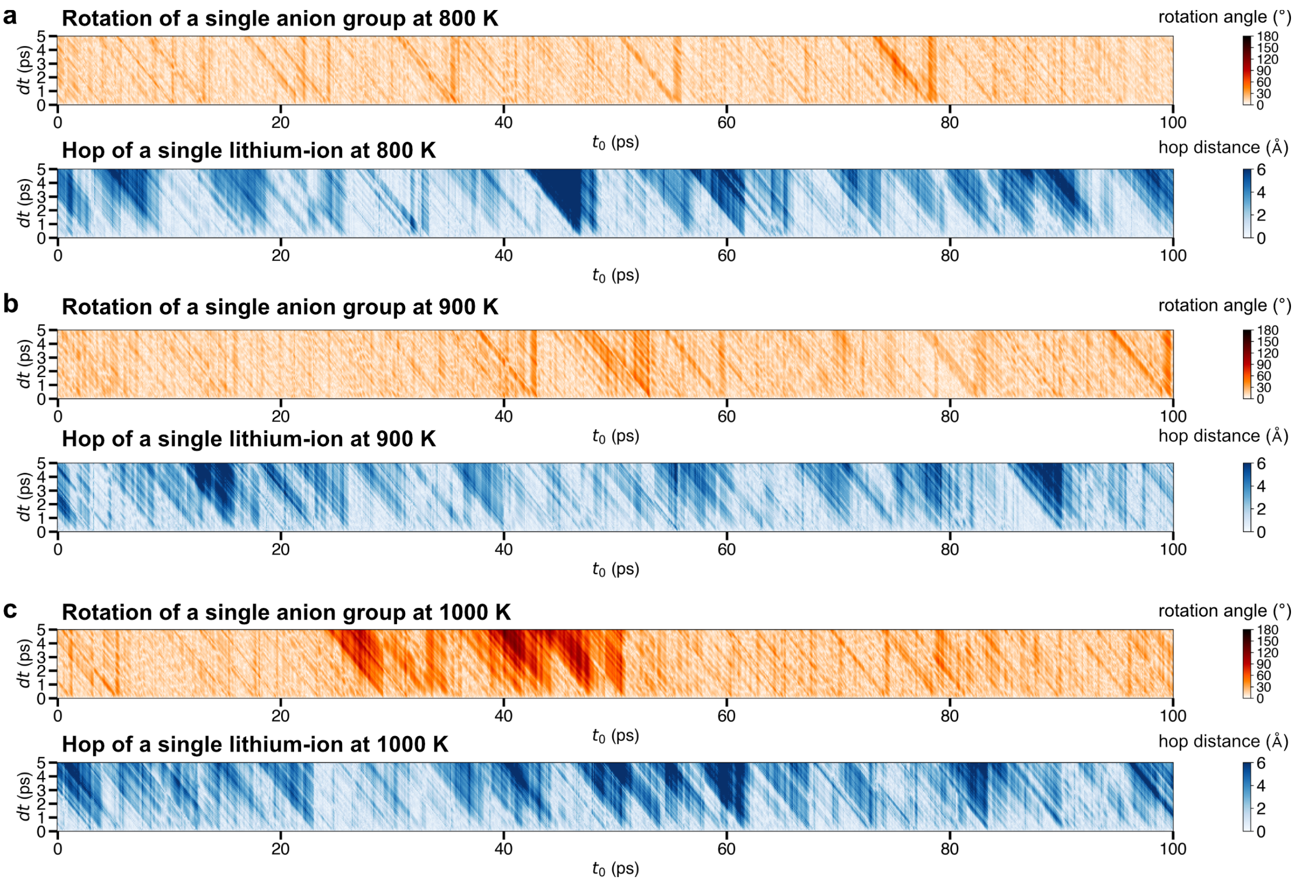

Figure S6. Exemplary 100-ps-long rotation and translation diagram that tracks a single anion group and a single lithium-ion in β-Li_3PS_4 computed at 800 K (a), 900 K (b), 1000 K (c). Note that the specified anion group and lithium-ion are not necessarily spatially close to each other.

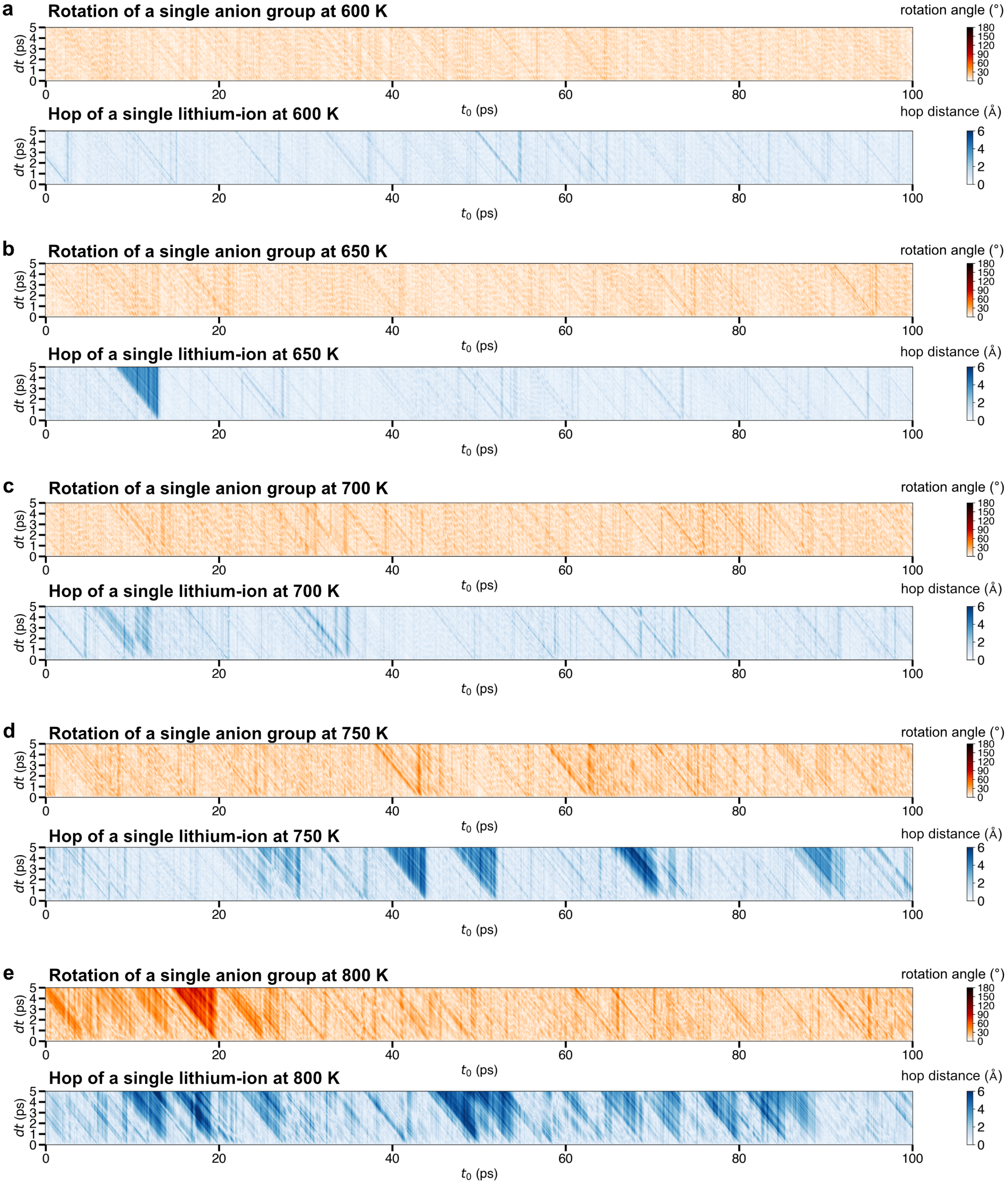

Figure S7. Exemplary 100-ps-long rotation and translation diagram that tracks a single anion group and a single lithium-ion in γ-Li_3PS_4 computed at 600 K (a), 650 K (b), 700 K (c), 750 K (d), 800 K (e). Note that the specified anion group and lithium-ion are not necessarily spatially close to each other.

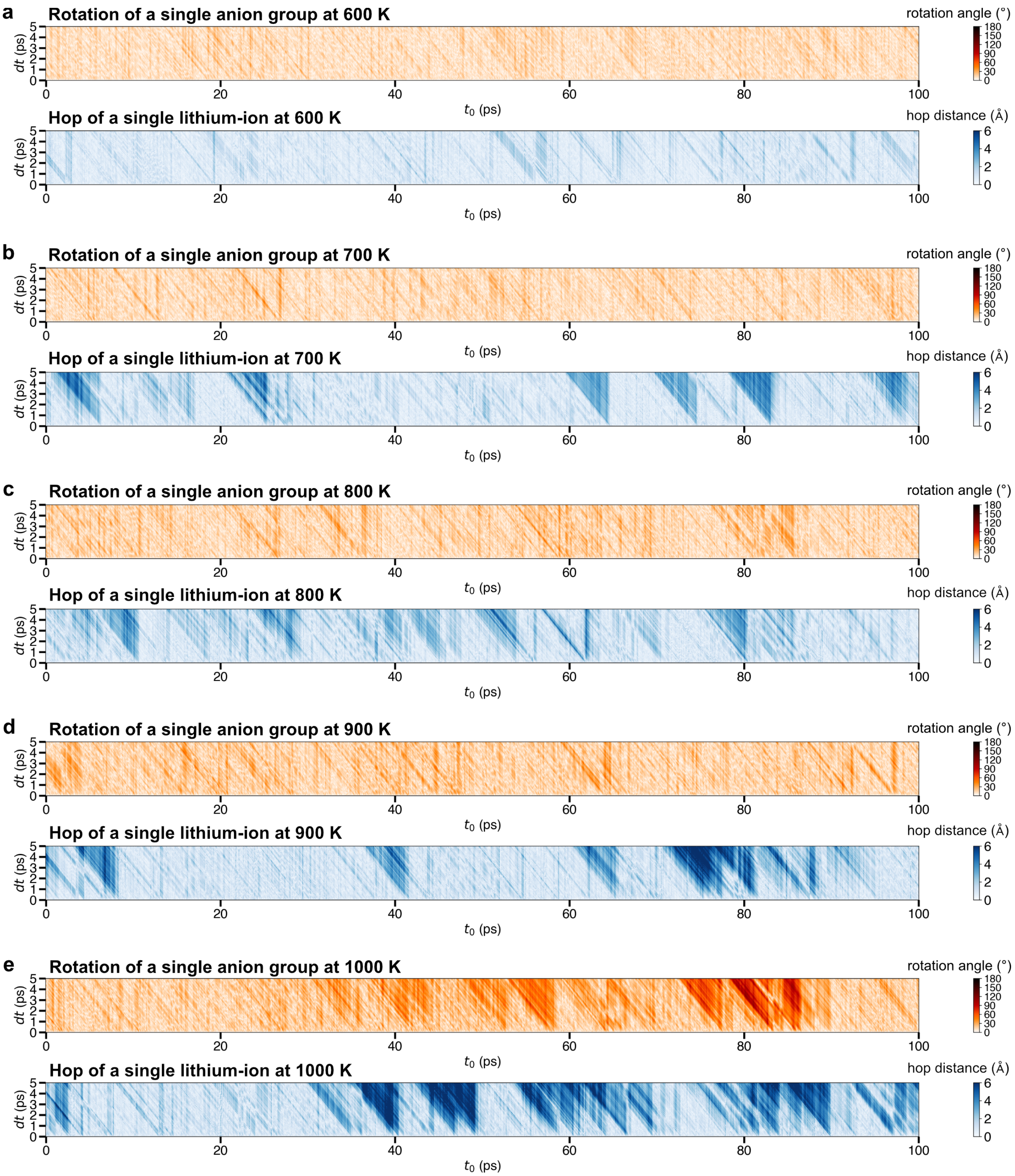

Figure S8. Exemplary 100-ps-long rotation and translation diagram that tracks a single anion group and a single lithium-ion in amorphous Li_3PS_4 (2.0 g/cm^3) computed at 600 K (a), 700 K (b), 800 K (c), 900 K (d), 1000 K (e). Note that the specified anion group and lithium-ion are not necessarily spatially close to each other.

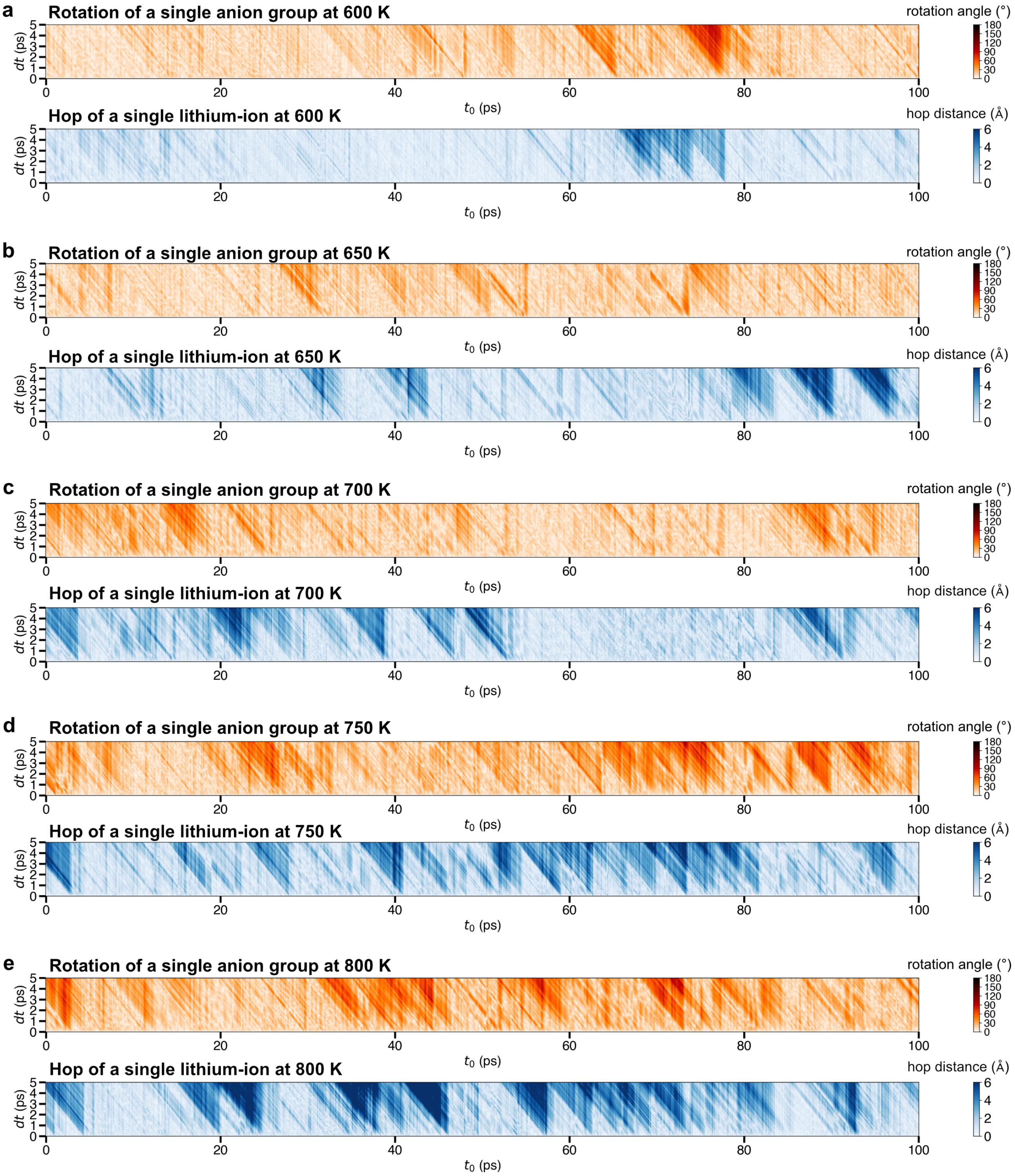

Figure S9. Exemplary 100-ps-long rotation and translation diagram that tracks a single anion group and a single lithium-ion in amorphous Li_3PS_4 (1.8 g/cm^3) computed at 600 K (a), 650 K (b), 700 K (c), 750 K (d), 800 K (e). Note that the specified anion group and lithium-ion are not necessarily spatially close to each other.

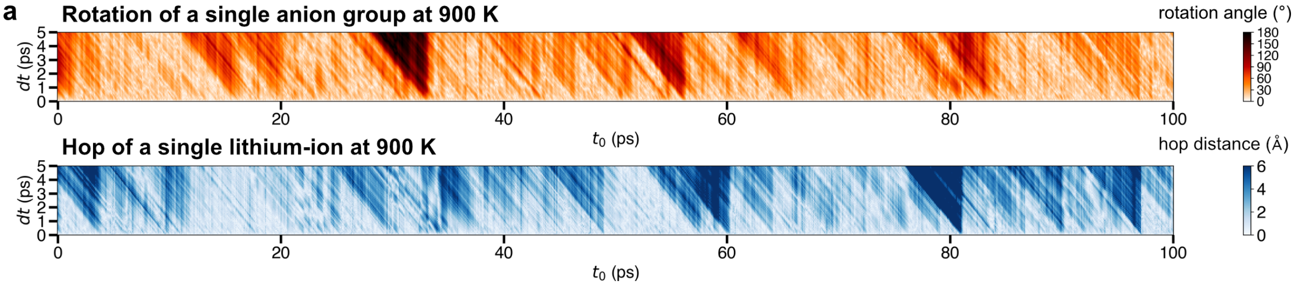

Figure S10. Exemplary 100-ps-long rotation and translation diagram that tracks a single anion group and a single lithium-ion in amorphous Li_3PS_4 (1.8 g/cm^3) computed at 900 K (a). Note that the specified anion group and lithium-ion are not necessarily spatially close to each other.

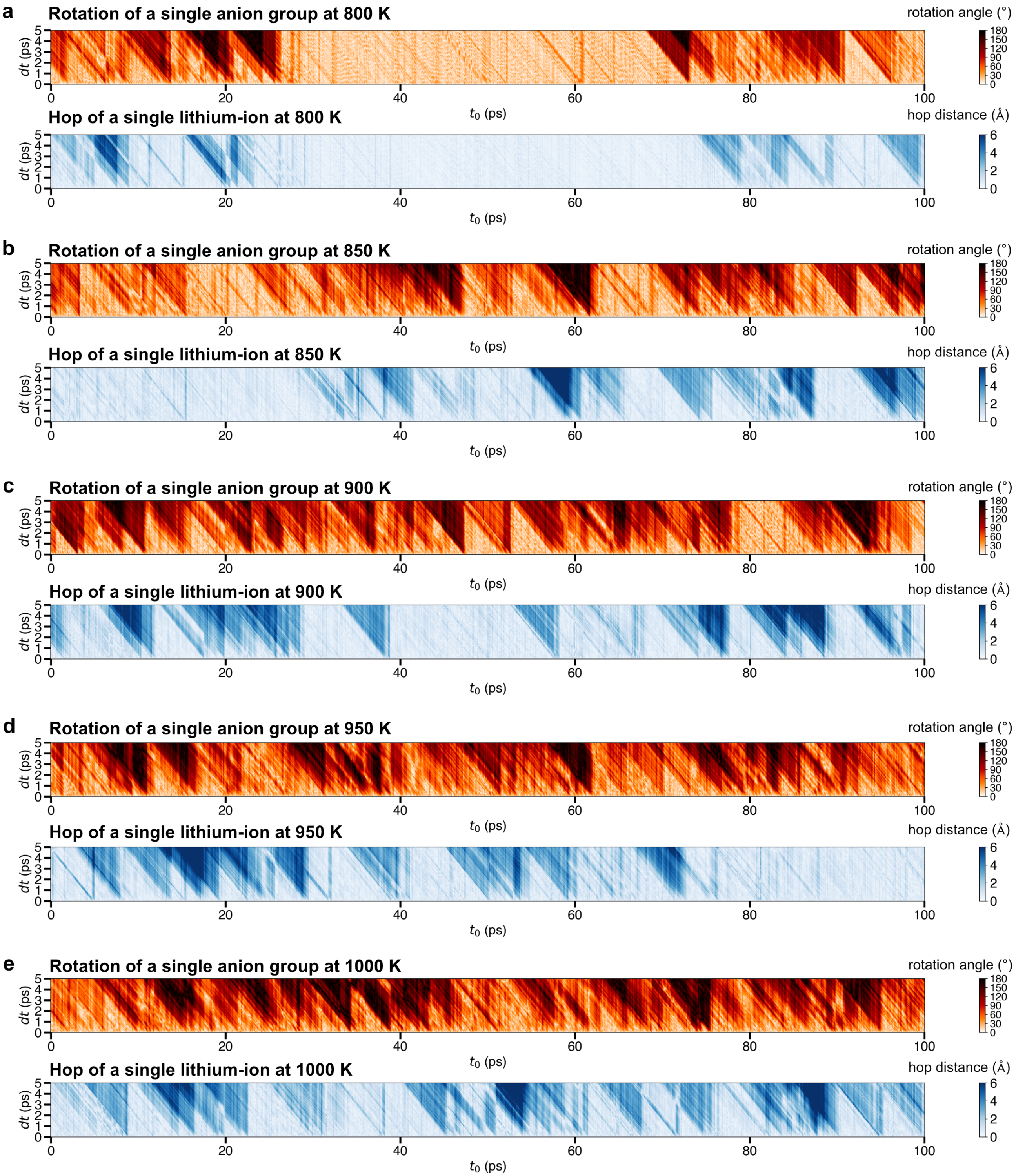

Figure S11. Exemplary 100-ps-long rotation and translation diagram that tracks a single anion group and a single lithium-ion in HT-Li_2SO_4 phase computed at 800 K (a), 850 K (b), 900 K (c), 950 K (d), 1000 K (e). Note that the specified anion group and lithium-ion are not necessarily spatially close to each other.

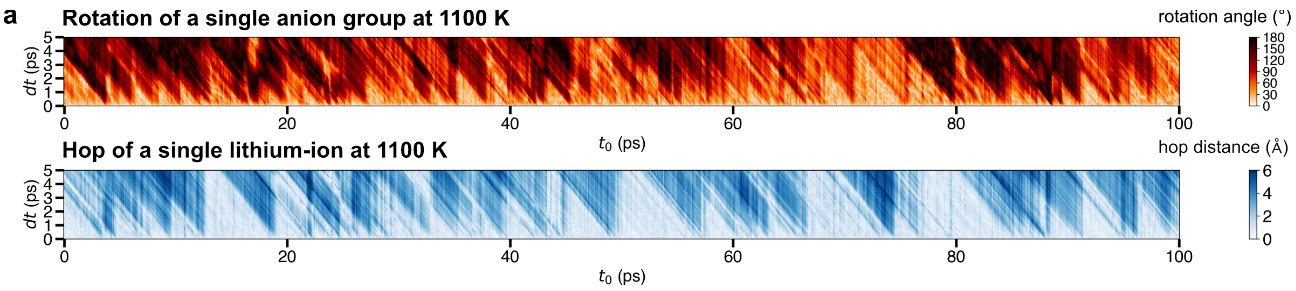

Figure S12. Exemplary 100-ps-long rotation and translation diagram that tracks a single anion group and a single lithium-ion in HT-Li_2SO_4 phase computed at 1100 K (a). Note that the specified anion group and lithium-ion are not necessarily spatially close to each other.

Table S2. *Activation energies of C3 rotation only, hop only and hop with a coherent C3 rotation computed for* β-Li_3PS_4 and HT-Li_2SO_4.

System	Type	Label	Figure	Rotation only (eV)	Rotation + hop (eV)	Hop only (eV)
β-Li_3PS_4	Stoichiometric	Path-S1	Figure S13	1.406 (before hop)	1.411	0.821
			Figure S13	1.395 (after hop)		
		Path-S2	Figure S14	1.565	1.883	0.916
	Vacancy	Path-V1	Figure S15	1.468	1.601	0.892
		Path-V2	Figure S16	1.635	1.462	0.123
		Path-V3	Figure 3	1.594	1.308	0.112
HT-Li_2SO_4	Stoichiometric	N/A	Figure S17	0.506	N/A	N/A

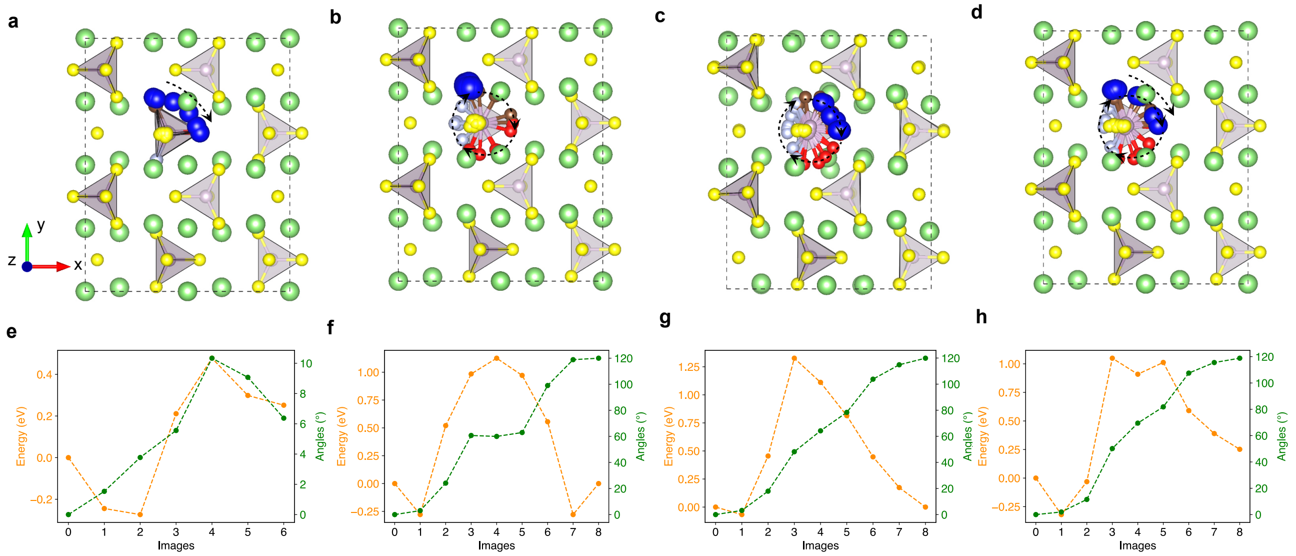

Figure S13. Minimum energy path of anion-group-assisted lithium-ion hops of path-S1 in stoichiometric β-Li_3PS_4. (a) Minimum energy pathway of a lithium-ion hop from 8d site to a vacant 4c site in β-Li_3PS_4. (b) Minimum energy pathway of a PS_4 tetrahedron making a 3-fold symmetry rotation. (c) Minimum energy pathway of a lithium-ion hop with a coherent PS_4 3-fold symmetry rotation. The yellow, green, and purple circles represent sulfur, lithium, and phosphorus atoms, respectively. The blue circle represents the lithium ion that is migrating. The red, brown, and light-blue circles and bonds represent the coordinating S^{2-} of the PS_4 that is rotating. (d–f) Energy barrier (orange) and angle of PS_4 from the initial orientation (green) for the hop only (d), rotation only (e), and hop with coherent rotation (f).

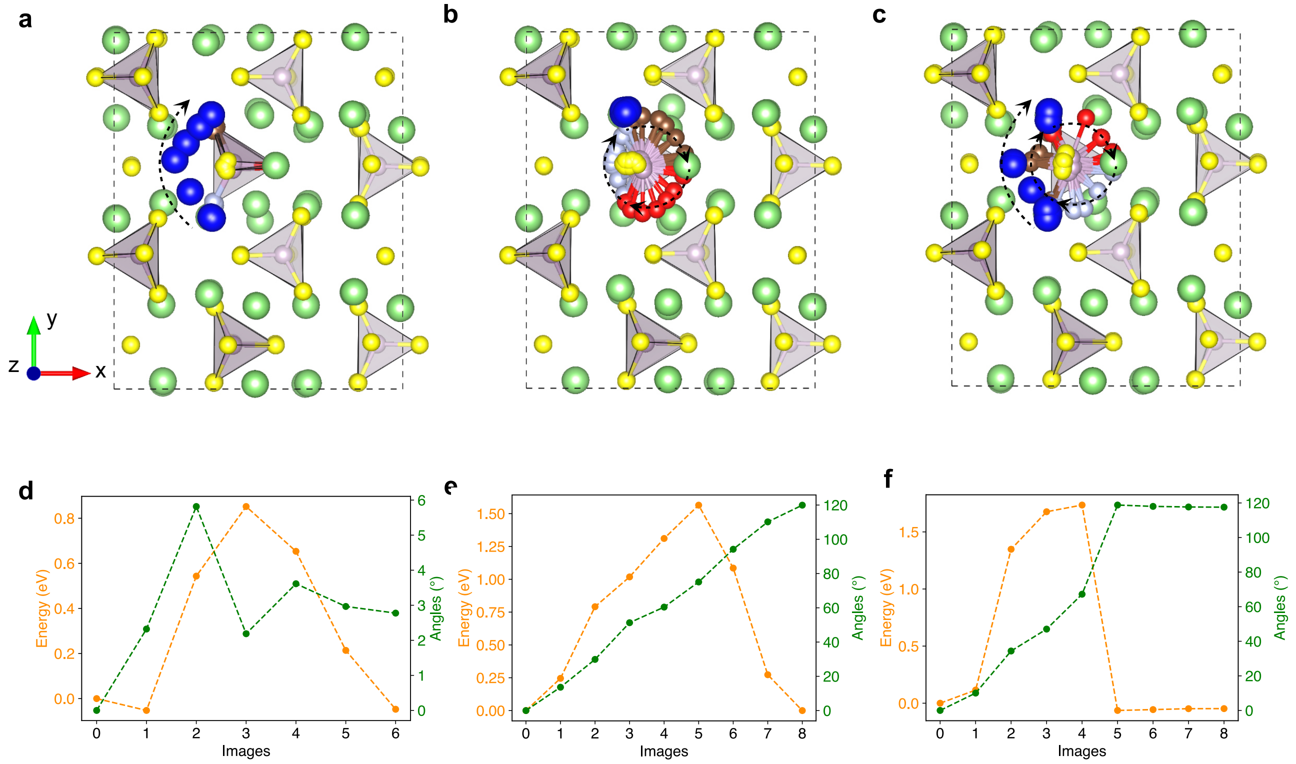

Figure S14. Minimum energy path of anion-group-assisted lithium-ion hops of path-S2 in stoichiometric β-Li_3PS_4. (a) Minimum energy pathway of a lithium-ion hop from 8d site to a vacant 8d site while maintaining the 4c interstitial in β-Li_3PS_4. (b) Minimum energy pathway of a PS_4 tetrahedron making a 3-fold symmetry rotation in the lithium position prior to the hop. (c) Minimum energy pathway of a PS_4 tetrahedron making a 3-fold symmetry rotation in the lithium position after to the hop. (d) Minimum energy pathway of a lithium-ion hop with a coherent PS_4 3-fold symmetry rotation. The yellow, green, and purple circles represent sulfur, lithium, and phosphorus atoms, respectively. The blue circle represents the lithium ion that is migrating. The red, brown, and light-blue circles and bonds represent the coordinating S^{2-} of the PS_4 that is rotating. (e–h) Energy barrier (orange) and angle of PS_4 from the initial orientation (green) for the hop only (e), rotation only (before hop) (f), rotation only (after hop) (g), and hop with coherent rotation (h).

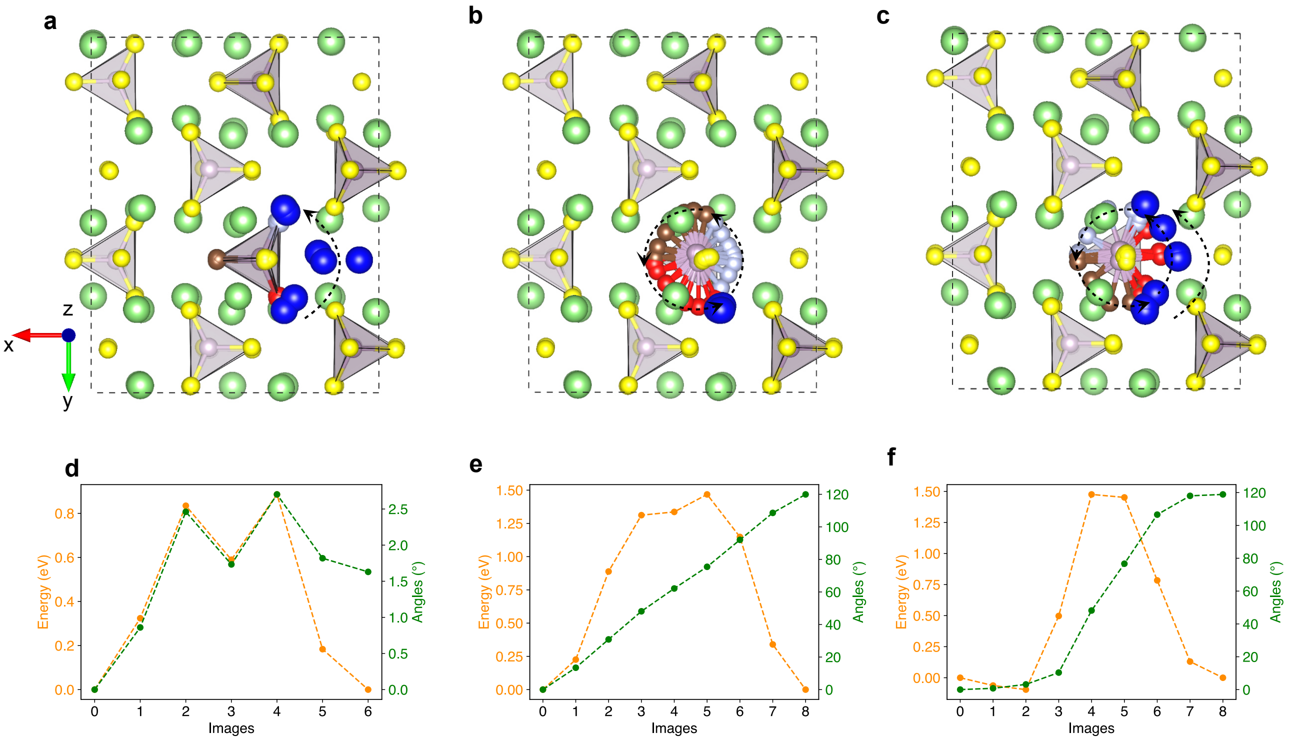

Figure S15. Minimum energy path of anion-group-assisted lithium-ion hops of path-V1 in β-Li_3PS_4 with a lithium-ion vacancy. (a) Minimum energy pathway of a lithium-ion hop from 8d site to a vacant 8d site while maintaining a common edge with an adjacent PS_4 group in β-Li_3PS_4. (b) Minimum energy pathway of a PS_4 tetrahedron making a 3-fold symmetry rotation. (c) Minimum energy pathway of a lithium-ion hop with a coherent PS_4 3-fold symmetry rotation. The yellow, green, and purple circles represent sulfur, lithium, and phosphorus atoms, respectively. The blue circle represents the lithium ion that is migrating. The red, brown, and light-blue circles and bonds represent the coordinating S^{2-} of the PS_4 that is rotating. (d–f) Energy barrier (orange) and angle of PS_4 from the initial orientation (green) for the hop only (d), rotation only (e), and hop with coherent rotation (f).

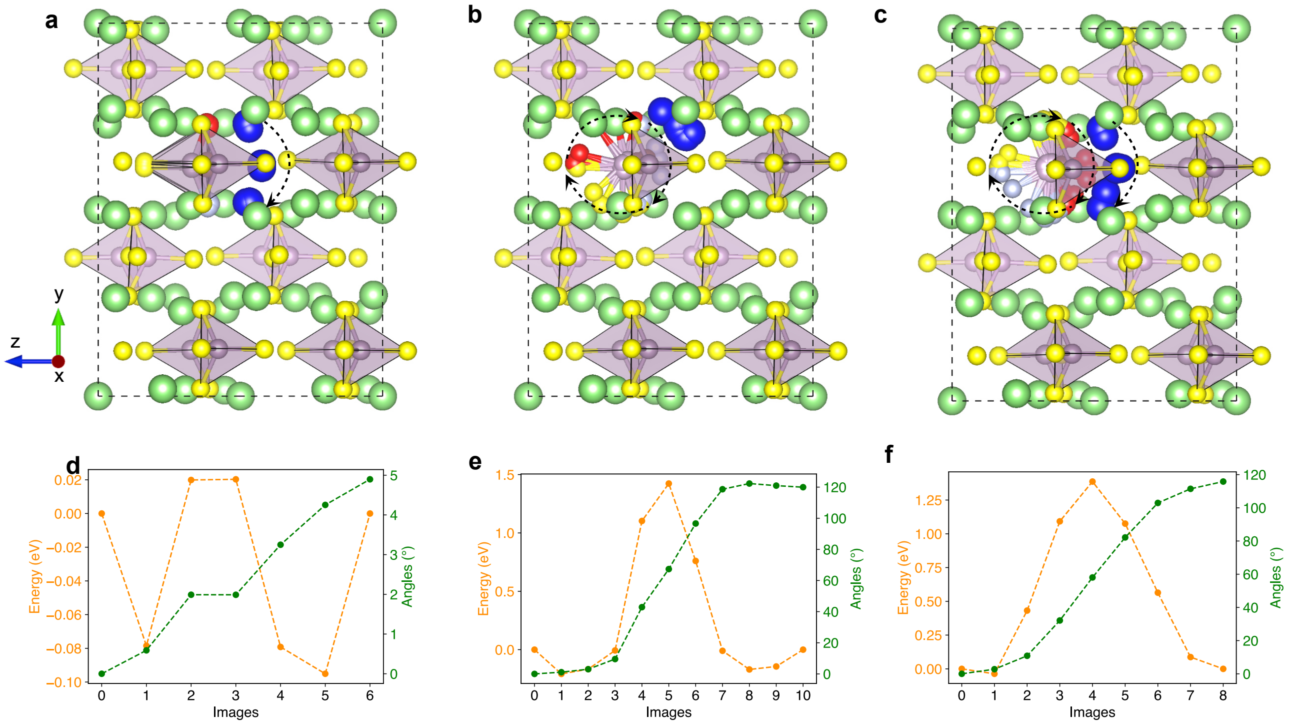

Figure S16. Minimum energy path of anion-group-assisted lithium-ion hops of path-V2 in β-Li_3PS_4 with a lithium-ion vacancy. (a) Minimum energy pathway of a lithium-ion hop from 4b site to a vacant 4b site while maintaining a common edge with an adjacent PS_4 group in β-Li_3PS_4. (b) Minimum energy pathway of a PS_4 tetrahedron making a 3-fold symmetry rotation. (c) Minimum energy pathway of a lithium-ion hop with a coherent PS_4 3-fold symmetry rotation. The yellow, green, and purple circles represent sulfur, lithium, and phosphorus atoms, respectively. The blue circle represents the lithium ion that is migrating. The red, brown, and light-blue circles and bonds represent the coordinating S^{2-} of the PS_4 that is rotating. (d–f) Energy barrier (orange) and angle of PS_4 from the initial orientation (green) for the hop only (d), rotation only (e), and hop with coherent rotation (f).

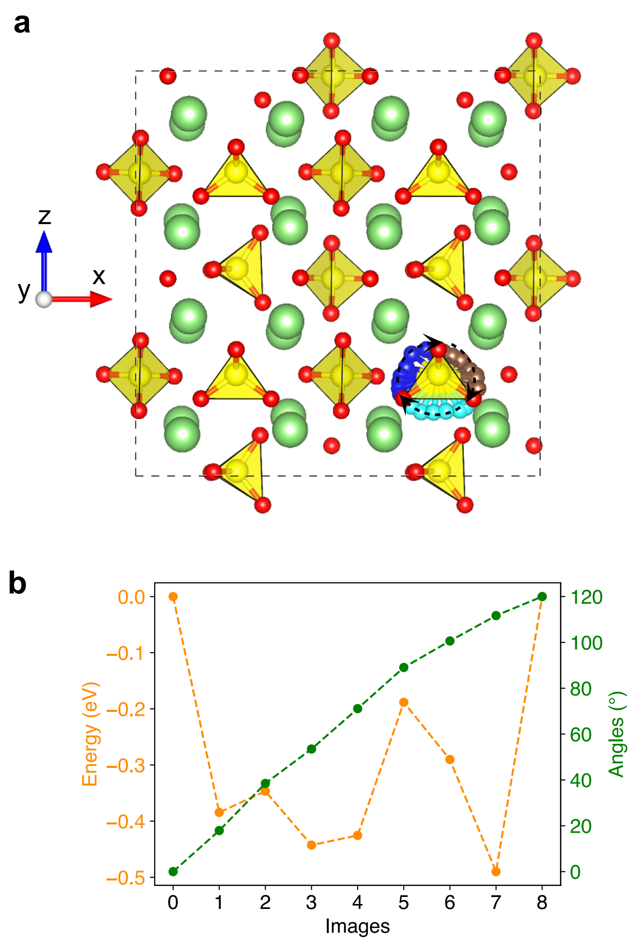

Figure S17. Minimum energy path of anion-group rotation in HT-Li_2SO_4 phase. (a) Minimum energy pathway of an SO_4 tetrahedron making a 3-fold symmetry rotation. The yellow, green, and red circles represent sulfur, lithium, and oxygen atoms, respectively. The red, brown, and light-blue circles and bonds represent the coordinating O^{2-} of the PO_4 that is rotating. (b) Energy barrier (orange) and angle of PO_4 from the initial orientation (green) for 120° rotation.

Note S2. Primitive cell of α-Li_3PS_4 used in analyzing the local occupancy vectors

```
# generated using pymatgen
data_Li3PS4
_symmetry_space_group_name_H-M   'P 1'
_cell_length_a   8.64350000
_cell_length_b   9.04620000
_cell_length_c   8.47790000
_cell_angle_alpha   90.00000000
_cell_angle_beta   90.00000000
_cell_angle_gamma   90.00000000
_symmetry_Int_Tables_number   1
_chemical_formula_structural   Li3PS4
_chemical_formula_sum   'Li12 P4 S16'
_cell_volume   662.89403511
_cell_formula_units_Z   4
loop_
 _symmetry_equiv_pos_site_id
 _symmetry_equiv_pos_as_xyz
  1  'x, y, z'
loop_
 _atom_type_symbol
 _atom_type_oxidation_number
  Li+  1.0
  P5+  5.0
  S2-  -2.0
loop_
 _atom_site_type_symbol
 _atom_site_label
 _atom_site_symmetry_multiplicity
 _atom_site_fract_x
 _atom_site_fract_y
 _atom_site_fract_z
 _atom_site_occupancy
  Li+  Li1  1  0.71400000  0.00000000  0.00000000 0.4152249134948097
  Li+  Li2  1  0.28600000  0.00000000  0.50000000 0.4152249134948097
  Li+  Li3  1  0.28600000  0.00000000  0.00000000 0.4152249134948097
  Li+  Li4  1  0.71400000  0.00000000  0.50000000 0.4152249134948097
  Li+  Li5  1  0.21400000  0.50000000  0.00000000 0.4152249134948097
  Li+  Li6  1  0.78600000  0.50000000  0.50000000 0.4152249134948097
  Li+  Li7  1  0.78600000  0.50000000  0.00000000 0.4152249134948097
  Li+  Li8  1  0.21400000  0.50000000  0.50000000 0.4152249134948097
  Li+  Li9  1  0.72400000  0.35300000  0.52800000 0.430795847750865
  Li+  Li10  1  0.27600000  0.64700000  0.02800000 0.430795847750865
  Li+  Li11  1  0.72400000  0.64700000  0.47200000 0.430795847750865
  Li+  Li12  1  0.27600000  0.35300000  0.97200000 0.430795847750865
  Li+  Li13  1  0.27600000  0.64700000  0.47200000 0.430795847750865
  Li+  Li14  1  0.72400000  0.35300000  0.97200000 0.430795847750865
  Li+  Li15  1  0.27600000  0.35300000  0.52800000 0.430795847750865
  Li+  Li16  1  0.72400000  0.64700000  0.02800000 0.430795847750865
  Li+  Li17  1  0.22400000  0.85300000  0.52800000 0.430795847750865
  Li+  Li18  1  0.77600000  0.14700000  0.02800000 0.430795847750865
  Li+  Li19  1  0.22400000  0.14700000  0.47200000 0.430795847750865
  Li+  Li20  1  0.77600000  0.85300000  0.97200000 0.430795847750865
  Li+  Li21  1  0.77600000  0.14700000  0.47200000 0.430795847750865
  Li+  Li22  1  0.22400000  0.85300000  0.97200000 0.430795847750865
  Li+  Li23  1  0.77600000  0.85300000  0.52800000 0.430795847750865
  Li+  Li24  1  0.22400000  0.14700000  0.02800000 0.430795847750865
  Li+  Li25  1  0.00000000  0.19600000  0.25000000 0.4463667820069204
  Li+  Li26  1  0.00000000  0.80400000  0.75000000 0.4463667820069204
  Li+  Li27  1  0.50000000  0.69600000  0.25000000 0.4463667820069204
  Li+  Li28  1  0.50000000  0.30400000  0.75000000 0.4463667820069204
  P5+  P1  1  0.00000000  0.83060000  0.25000000  1.0 #Main-Figure5a
  P5+  P2  1  0.00000000  0.16940000  0.75000000  1.0 #SI-FigureS18a
  P5+  P3  1  0.50000000  0.33060000  0.25000000  1.0 #SI-FigureS18b
  P5+  P4  1  0.50000000  0.66940000  0.75000000  1.0 #SI-FigureS18c
  S2-  S1  1  0.30420000  0.45600000  0.25000000  1.0
  S2-  S2  1  0.69580000  0.54400000  0.75000000  1.0
  S2-  S3  1  0.30420000  0.54400000  0.75000000  1.0
  S2-  S4  1  0.69580000  0.45600000  0.25000000  1.0
  S2-  S5  1  0.80420000  0.95600000  0.25000000  1.0
  S2-  S6  1  0.19580000  0.04400000  0.75000000  1.0
  S2-  S7  1  0.80420000  0.04400000  0.75000000  1.0
  S2-  S8  1  0.19580000  0.95600000  0.25000000  1.0
  S2-  S9  1  0.00000000  0.29490000  0.55370000  1.0
  S2-  S10  1  0.00000000  0.70510000  0.05370000  1.0
  S2-  S11  1  0.00000000  0.70510000  0.44630000  1.0
  S2-  S12  1  0.00000000  0.29490000  0.94630000  1.0
  S2-  S13  1  0.50000000  0.79490000  0.55370000  1.0
  S2-  S14  1  0.50000000  0.20510000  0.05370000  1.0
  S2-  S15  1  0.50000000  0.20510000  0.44630000  1.0
  S2-  S16  1  0.50000000  0.79490000  0.94630000  1.0
```

Note S3. Primitive cell of β-Li_3PS_4 used in analyzing the local occupancy vectors

```
# generated using pymatgen
data_Li3PS4
_symmetry_space_group_name_H-M   'P 1'
_cell_length_a   12.84830000
_cell_length_b   8.27720000
_cell_length_c   6.15120000
_cell_angle_alpha   90.00000000
_cell_angle_beta   90.00000000
_cell_angle_gamma   90.00000000
_symmetry_Int_Tables_number   1
_chemical_formula_structural   Li3PS4
_chemical_formula_sum   'Li12 P4 S16'
_cell_volume   654.16750241
_cell_formula_units_Z   4
loop_
 _symmetry_equiv_pos_site_id
 _symmetry_equiv_pos_as_xyz
  1  'x, y, z'
loop_
 _atom_type_symbol
 _atom_type_oxidation_number
  Li+  1.0
  P5+  5.0
  S2-  -2.0
loop_
 _atom_site_type_symbol
 _atom_site_label
 _atom_site_symmetry_multiplicity
 _atom_site_fract_x
 _atom_site_fract_y
 _atom_site_fract_z
 _atom_site_occupancy
  Li+  Li1  1  0.15900000  0.49600000  0.62900000  0.334
  Li+  Li2  1  0.15900000  0.00400000  0.62900000  0.334
  Li+  Li3  1  0.65900000  0.00400000  0.87100000  0.334
  Li+  Li4  1  0.34100000  0.50400000  0.12900000  0.334
  Li+  Li5  1  0.84100000  0.99600000  0.37100000  0.334
  Li+  Li6  1  0.34100000  0.99600000  0.12900000  0.334
  Li+  Li7  1  0.65900000  0.49600000  0.87100000  0.334
  Li+  Li8  1  0.84100000  0.50400000  0.37100000  0.334
  Li+  Li9  1  0.99100000  0.54500000  0.41800000  0.356
  Li+  Li10  1  0.99100000  0.95500000  0.41800000  0.356
  Li+  Li11  1  0.49100000  0.95500000  0.08200000  0.356
  Li+  Li12  1  0.50900000  0.45500000  0.91800000  0.356
  Li+  Li13  1  0.00900000  0.04500000  0.58200000  0.356
  Li+  Li14  1  0.50900000  0.04500000  0.91800000  0.356
  Li+  Li15  1  0.49100000  0.54500000  0.08200000  0.356
  Li+  Li16  1  0.00900000  0.45500000  0.58200000  0.356
  Li+  Li17  1  0.15100000  0.53200000  0.89600000  0.666
  Li+  Li18  1  0.15100000  0.96800000  0.89600000  0.666
  Li+  Li19  1  0.65100000  0.96800000  0.60400000  0.666
  Li+  Li20  1  0.34900000  0.46800000  0.39600000  0.666
  Li+  Li21  1  0.84900000  0.03200000  0.10400000  0.666
  Li+  Li22  1  0.34900000  0.03200000  0.39600000  0.666
  Li+  Li23  1  0.65100000  0.53200000  0.60400000  0.666
  Li+  Li24  1  0.84900000  0.46800000  0.10400000  0.666
  Li+  Li25  1  0.08400000  0.75000000  0.19600000  0.288
  Li+  Li26  1  0.58400000  0.75000000  0.30400000  0.288
  Li+  Li27  1  0.41600000  0.25000000  0.69600000  0.288
  Li+  Li28  1  0.91600000  0.25000000  0.80400000  0.288
  P5+  P1  1  0.91200000  0.75000000  0.82300000  1.0 # Main-Figure5b
  P5+  P2  1  0.41200000  0.75000000  0.67700000  1.0 # SI-FigureS19a
  P5+  P3  1  0.58800000  0.25000000  0.32300000  1.0 # SI-FigureS19b
  P5+  P4  1  0.08800000  0.25000000  0.17700000  1.0 # SI-FigureS19c
  S2-  S1  1  0.84500000  0.54800000  0.70200000  1.0
  S2-  S2  1  0.84500000  0.95200000  0.70200000  1.0
  S2-  S3  1  0.34500000  0.95200000  0.79800000  1.0
  S2-  S4  1  0.65500000  0.45200000  0.20200000  1.0
  S2-  S5  1  0.15500000  0.04800000  0.29800000  1.0
  S2-  S6  1  0.65500000  0.04800000  0.20200000  1.0
  S2-  S7  1  0.34500000  0.54800000  0.79800000  1.0
  S2-  S8  1  0.15500000  0.45200000  0.29800000  1.0
  S2-  S9  1  0.06400000  0.75000000  0.74600000  1.0
  S2-  S10  1  0.56400000  0.75000000  0.75400000  1.0
  S2-  S11  1  0.43600000  0.25000000  0.24600000  1.0
  S2-  S12  1  0.93600000  0.25000000  0.25400000  1.0
  S2-  S13  1  0.89500000  0.75000000  0.15400000  1.0
  S2-  S14  1  0.39500000  0.75000000  0.34600000  1.0
  S2-  S15  1  0.60500000  0.25000000  0.65400000  1.0
  S2-  S16  1  0.10500000  0.25000000  0.84600000  1.0
```

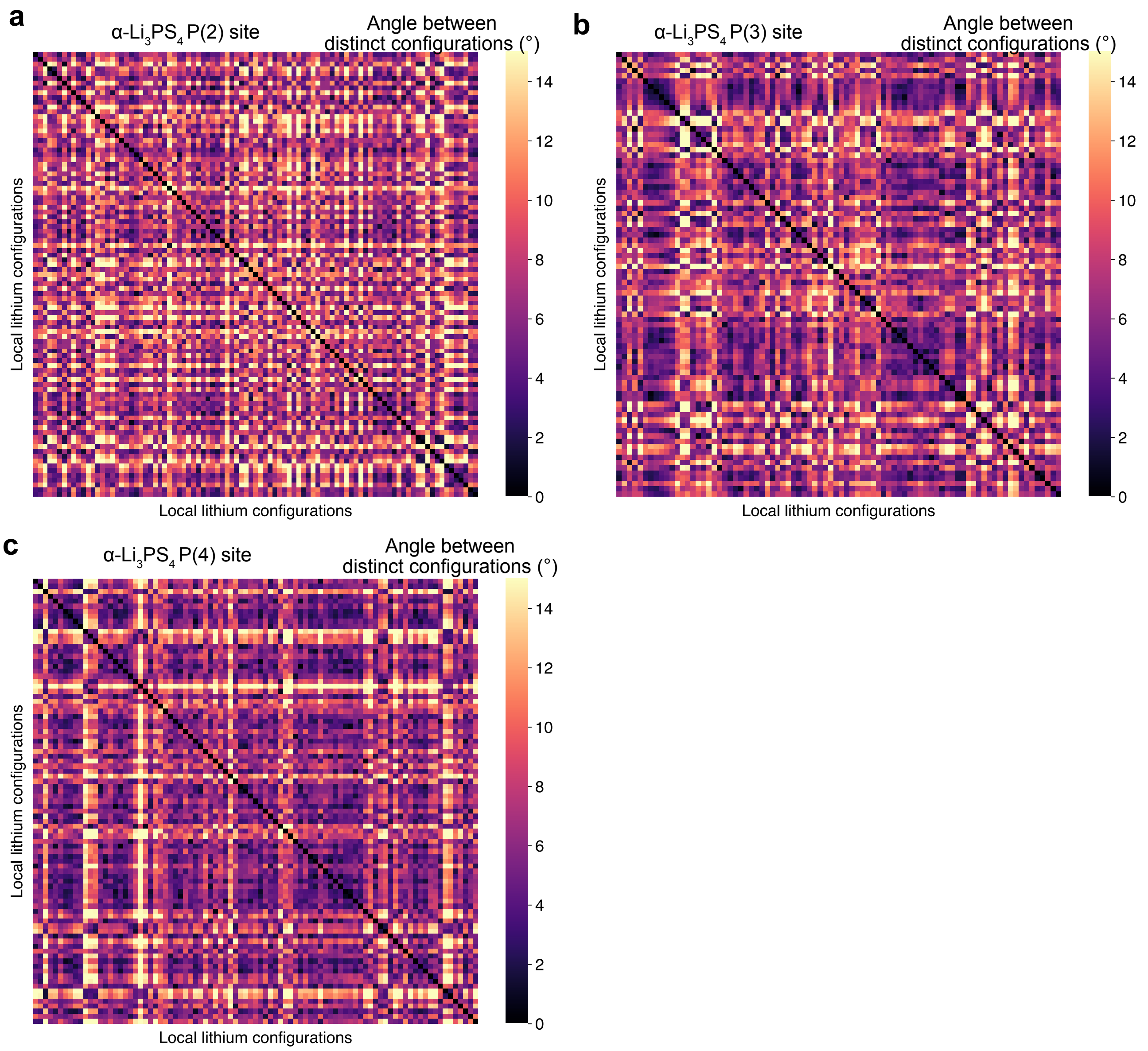

Figure S18. Angle (quaternion distance) between the PS_4 orientations for all pairs of distinct local occupancy vectors in α-Li_3PS_4. All P sites of the relaxed enumerated structures of various supercell sizes are mapped back to the P(1) site, P(2) site, P(3) site, and P(4) site of the primitive cell of α-Li_3PS_4. Results for P(2) site, P(3) site, and P(4) site are plotted in (a), (b), and (c) respectively.

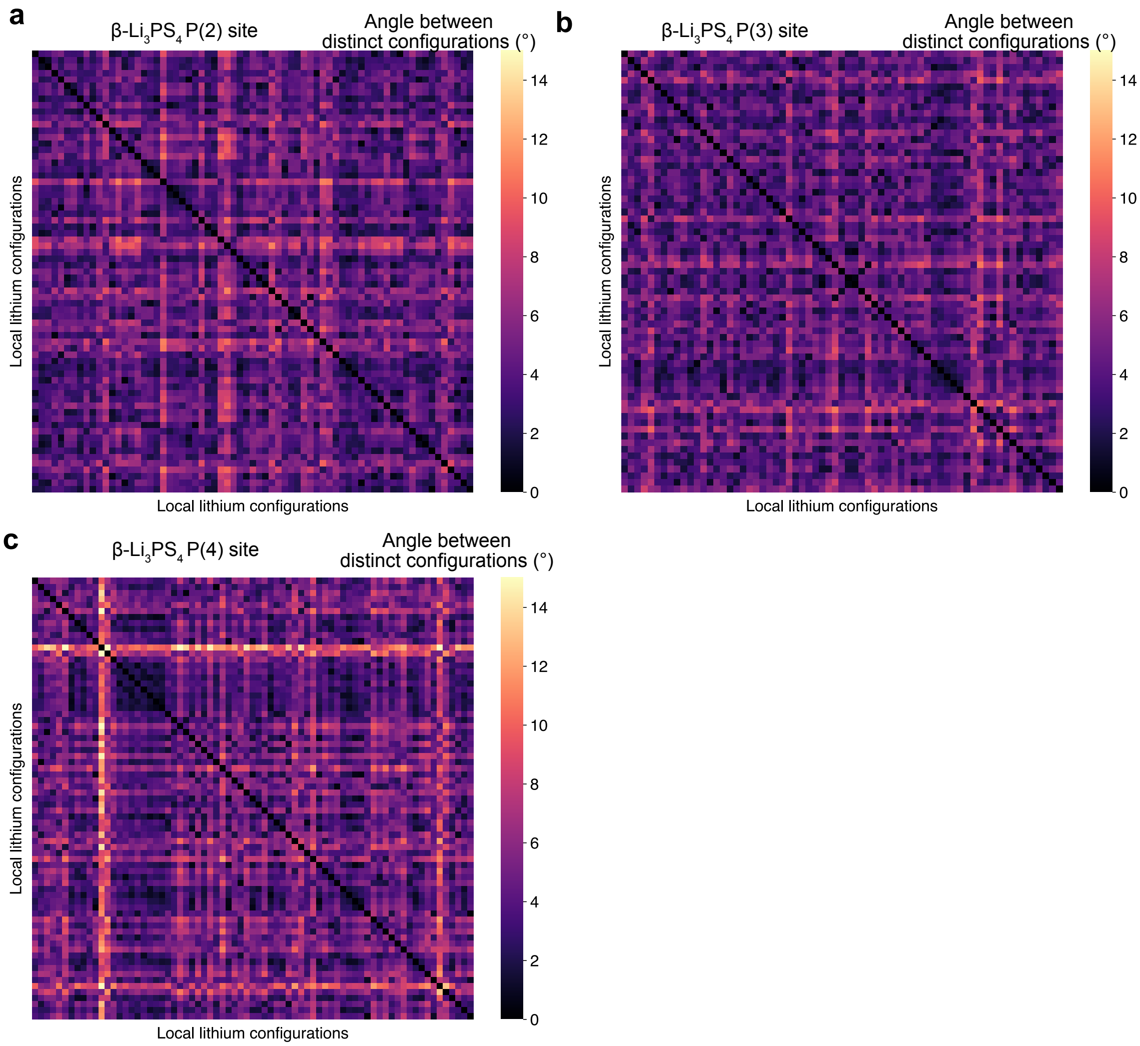

Figure S19. Angle (quaternion distance) between the PS_4 orientations for all pairs of distinct local occupancy vectors in β-Li_3PS_4. All P sites of the relaxed enumerated structures of various supercell sizes are mapped back to the P(1) site, P(2) site, P(3) site, and P(4) site of the primitive cell of β -Li_3PS_4. Results for P(2) site, P(3) site, and P(4) site are plotted in (a), (b), and (c) respectively.

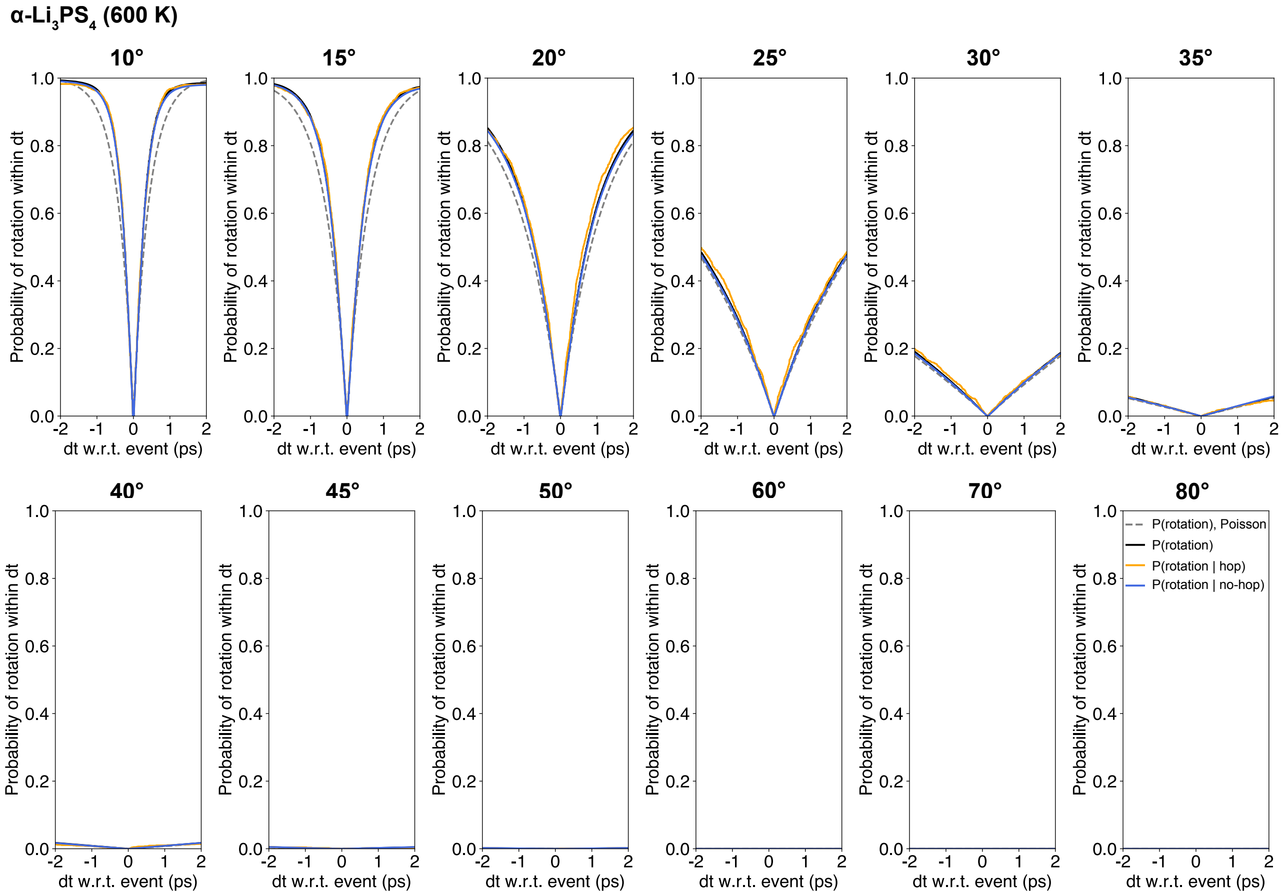

Figure S20. Full probability analysis of α-Li_3PS_4 at 600 K for rotational motions from 10° to 80°. Orange lines plot the conditional probability of a rotation event of cutoff angle θ given that a lithium-hop occurs at dt = 0. Blue lines plot the conditional probability of a rotation event of cutoff angle θ given that no lithium-hop occurs within ± 2 ps range of dt = 0. Black lines plot the probability of a rotation event of cutoff angle θ regardless of lithium-hops. Dashed grey lines show the probability of a single PS_4 rotation event of cutoff angle θ occurring within ± dt assuming that rotation events follow a Poisson process.

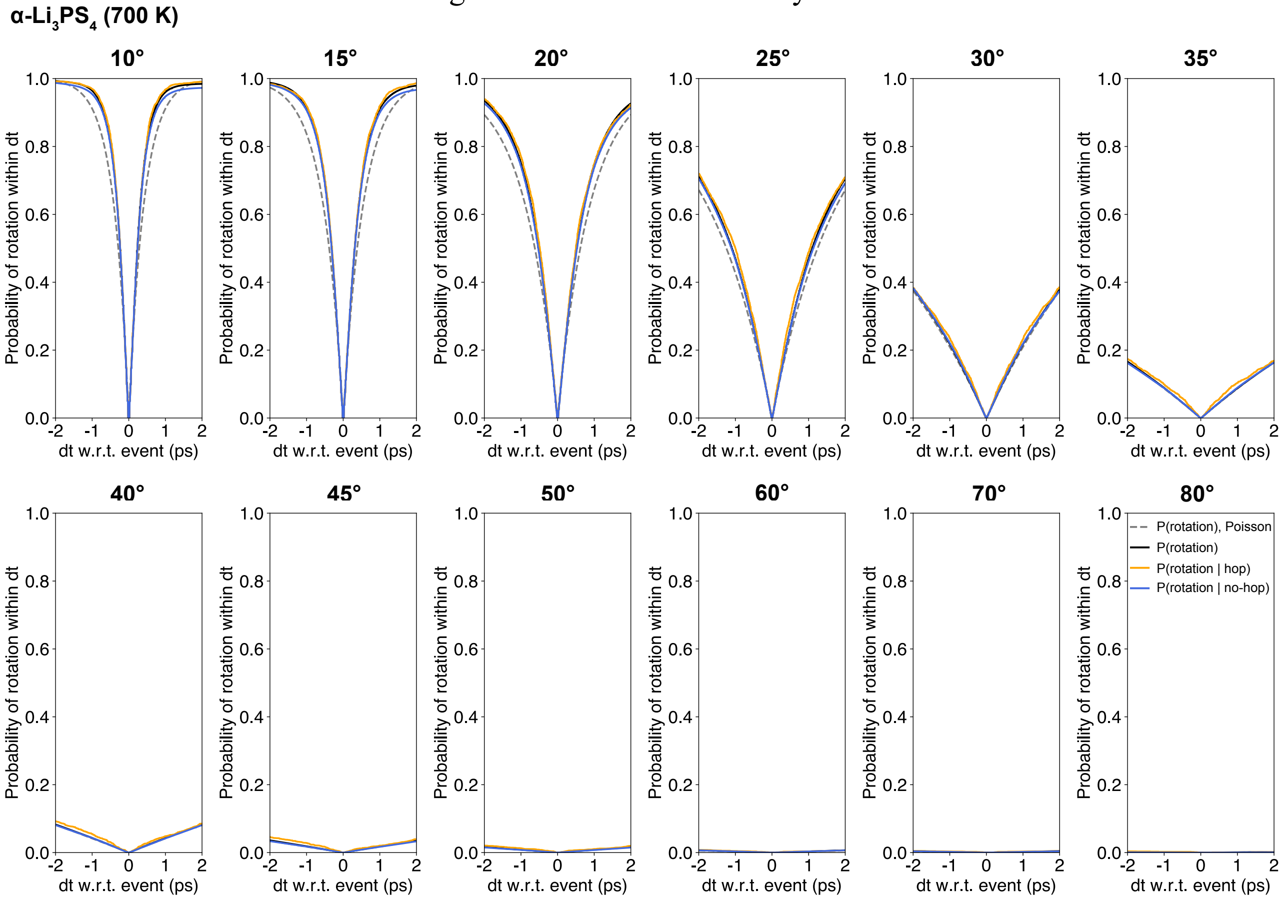

Figure S21. Full probability analysis of α-Li_3PS_4 at 700 K for rotational motions from 10° to 80°. Orange lines plot the conditional probability of a rotation event of cutoff angle θ given that a lithium-hop occurs at dt = 0. Blue lines plot the conditional probability of a rotation event of cutoff angle θ given that no lithium-hop occurs within ± 2 ps range of dt = 0. Black lines plot the probability of a rotation event of cutoff angle θ regardless of lithium-hops. Dashed grey lines show the probability of a single PS_4 rotation event of cutoff angle θ occurring within ± dt assuming that rotation events follow a Poisson process.

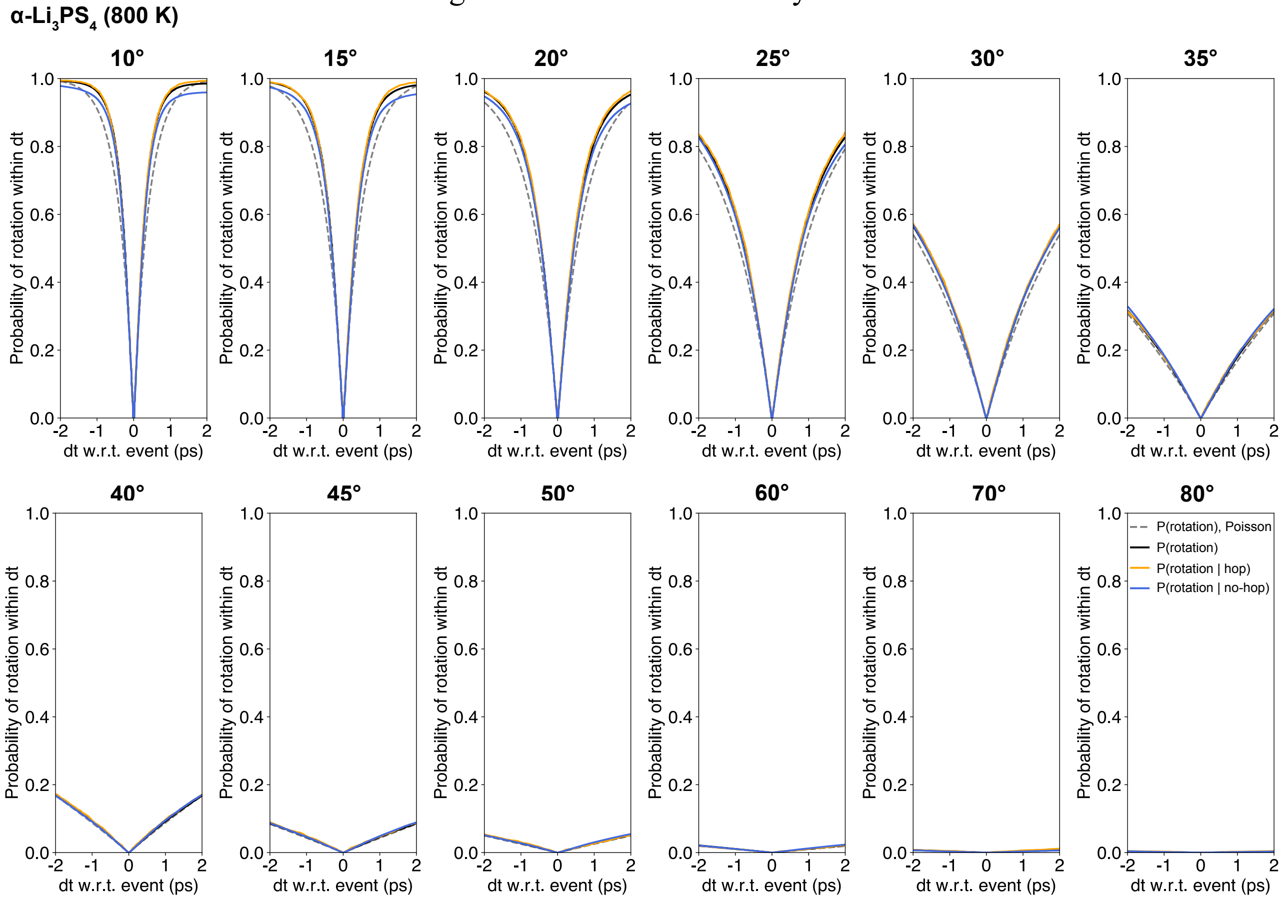

Figure S22. Full probability analysis of α-Li_3PS_4 at 800 K for rotational motions from 10° to 80°. Orange lines plot the conditional probability of a rotation event of cutoff angle θ given that a lithium-hop occurs at dt = 0. Blue lines plot the conditional probability of a rotation event of cutoff angle θ given that no lithium-hop occurs within ± 2 ps range of dt = 0. Black lines plot the probability of a rotation event of cutoff angle θ regardless of lithium-hops. Dashed grey lines show the probability of a single PS_4 rotation event of cutoff angle θ occurring within ± dt assuming that rotation events follow a Poisson process.

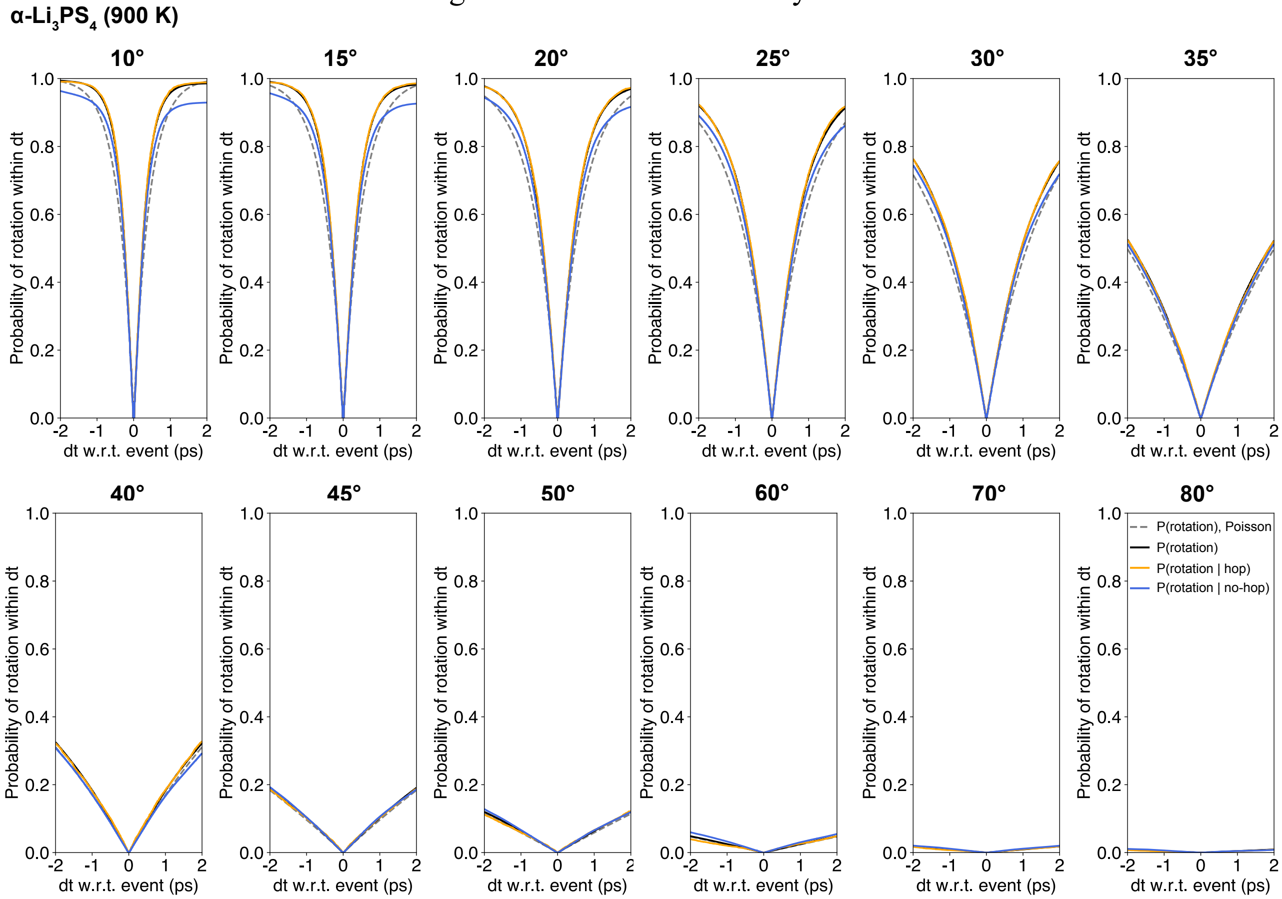

Figure S23. Full probability analysis of α-Li_3PS_4 at 900 K for rotational motions from 10° to 80°. Orange lines plot the conditional probability of a rotation event of cutoff angle θ given that a lithium-hop occurs at dt = 0. Blue lines plot the conditional probability of a rotation event of cutoff angle θ given that no lithium-hop occurs within ± 2 ps range of dt = 0. Black lines plot the probability of a rotation event of cutoff angle θ regardless of lithium-hops. Dashed grey lines show the probability of a single PS_4 rotation event of cutoff angle θ occurring within ± dt assuming that rotation events follow a Poisson process.

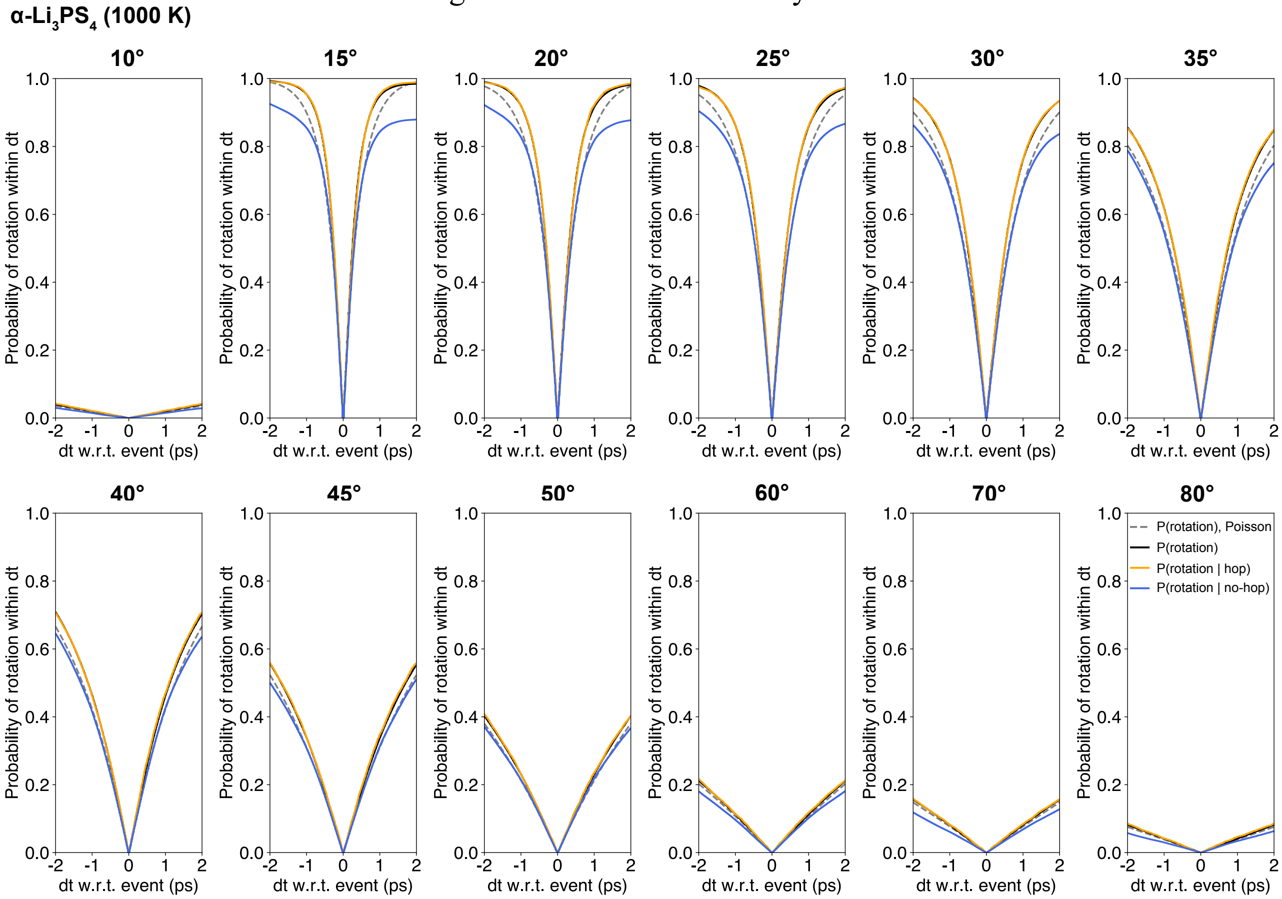

Figure S24. Full probability analysis of α-Li_3PS_4 at 1000 K for rotational motions from 10° to 80°. Orange lines plot the conditional probability of a rotation event of cutoff angle θ given that a lithium-hop occurs at dt = 0. Blue lines plot the conditional probability of a rotation event of cutoff angle θ given that no lithium-hop occurs within ± 2 ps range of dt = 0. Black lines plot the probability of a rotation event of cutoff angle θ regardless of lithium-hops. Dashed grey lines show the probability of a single PS_4 rotation event of cutoff angle θ occurring within ± dt assuming that rotation events follow a Poisson process.

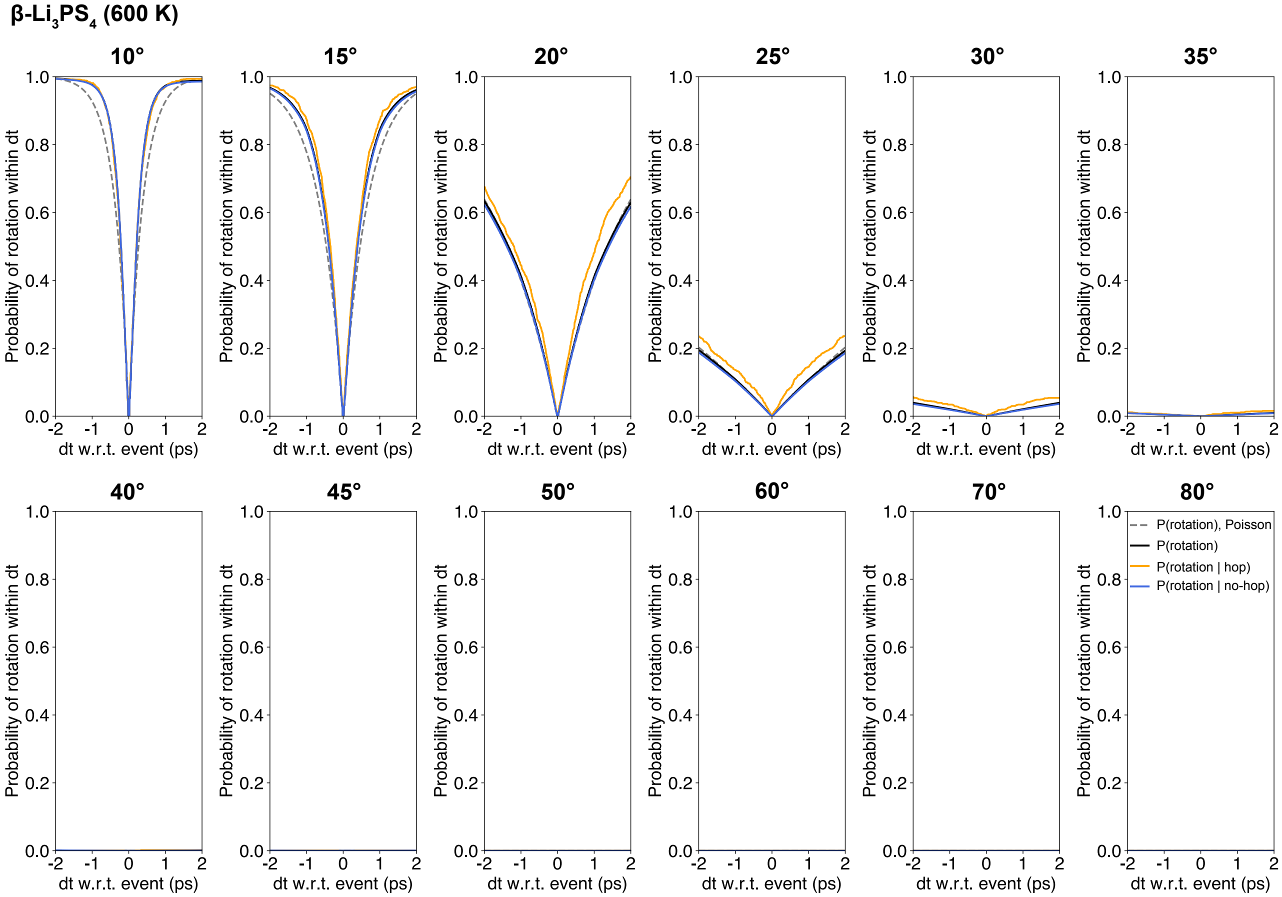

Figure S25. Full probability analysis of β-Li_3PS_4 at 600 K for rotational motions from 10° to 80°. Orange lines plot the conditional probability of a rotation event of cutoff angle θ given that a lithium-hop occurs at dt = 0. Blue lines plot the conditional probability of a rotation event of cutoff angle θ given that no lithium-hop occurs within ± 2 ps range of dt = 0. Black lines plot the probability of a rotation event of cutoff angle θ regardless of lithium-hops. Dashed grey lines show the probability of a single PS_4 rotation event of cutoff angle θ occurring within ± dt assuming that rotation events follow a Poisson process.

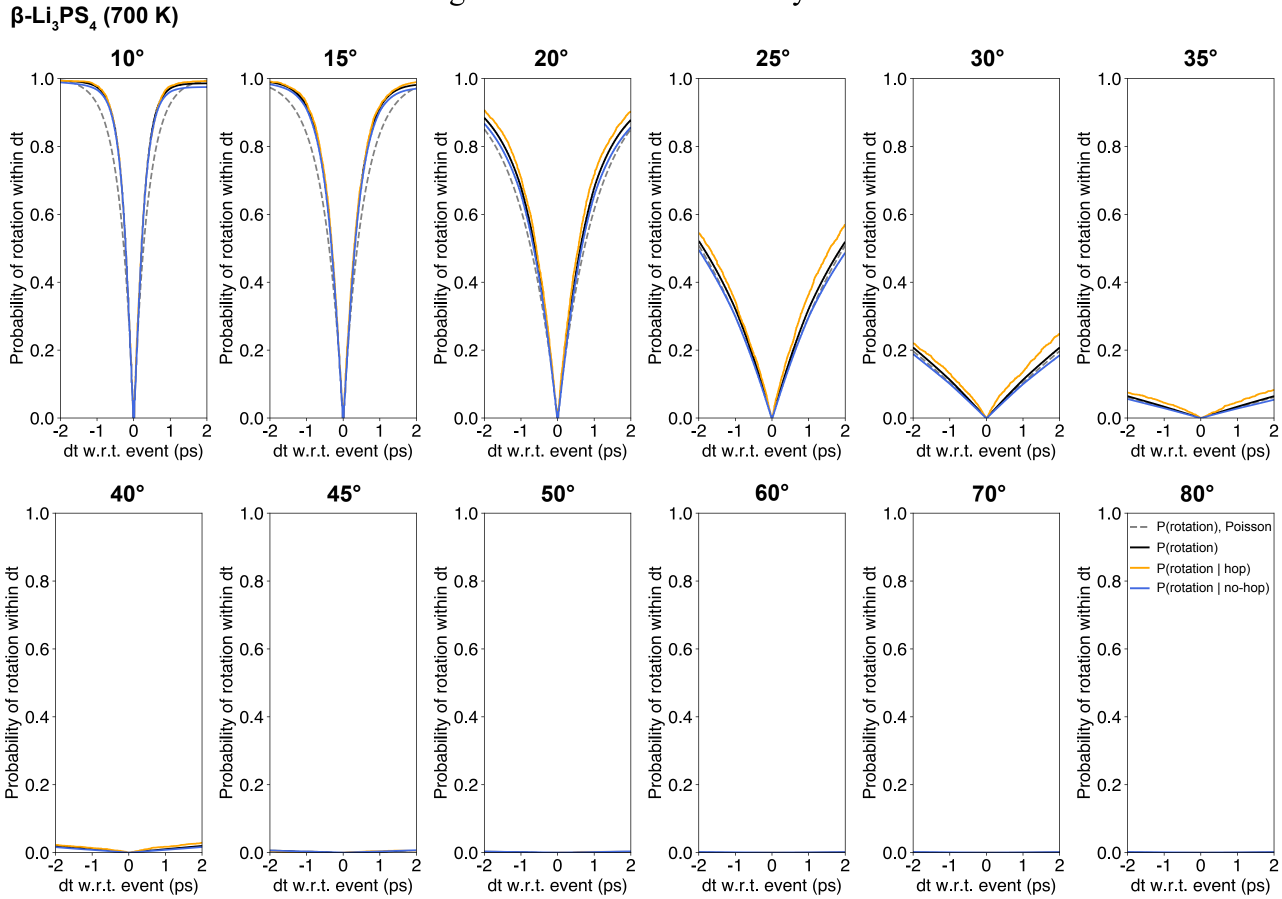

Figure S26. Full probability analysis of β-Li_3PS_4 at 700 K for rotational motions from 10° to 80°. Orange lines plot the conditional probability of a rotation event of cutoff angle θ given that a lithium-hop occurs at dt = 0. Blue lines plot the conditional probability of a rotation event of cutoff angle θ given that no lithium-hop occurs within ± 2 ps range of dt = 0. Black lines plot the probability of a rotation event of cutoff angle θ regardless of lithium-hops. Dashed grey lines show the probability of a single PS_4 rotation event of cutoff angle θ occurring within ± dt assuming that rotation events follow a Poisson process.

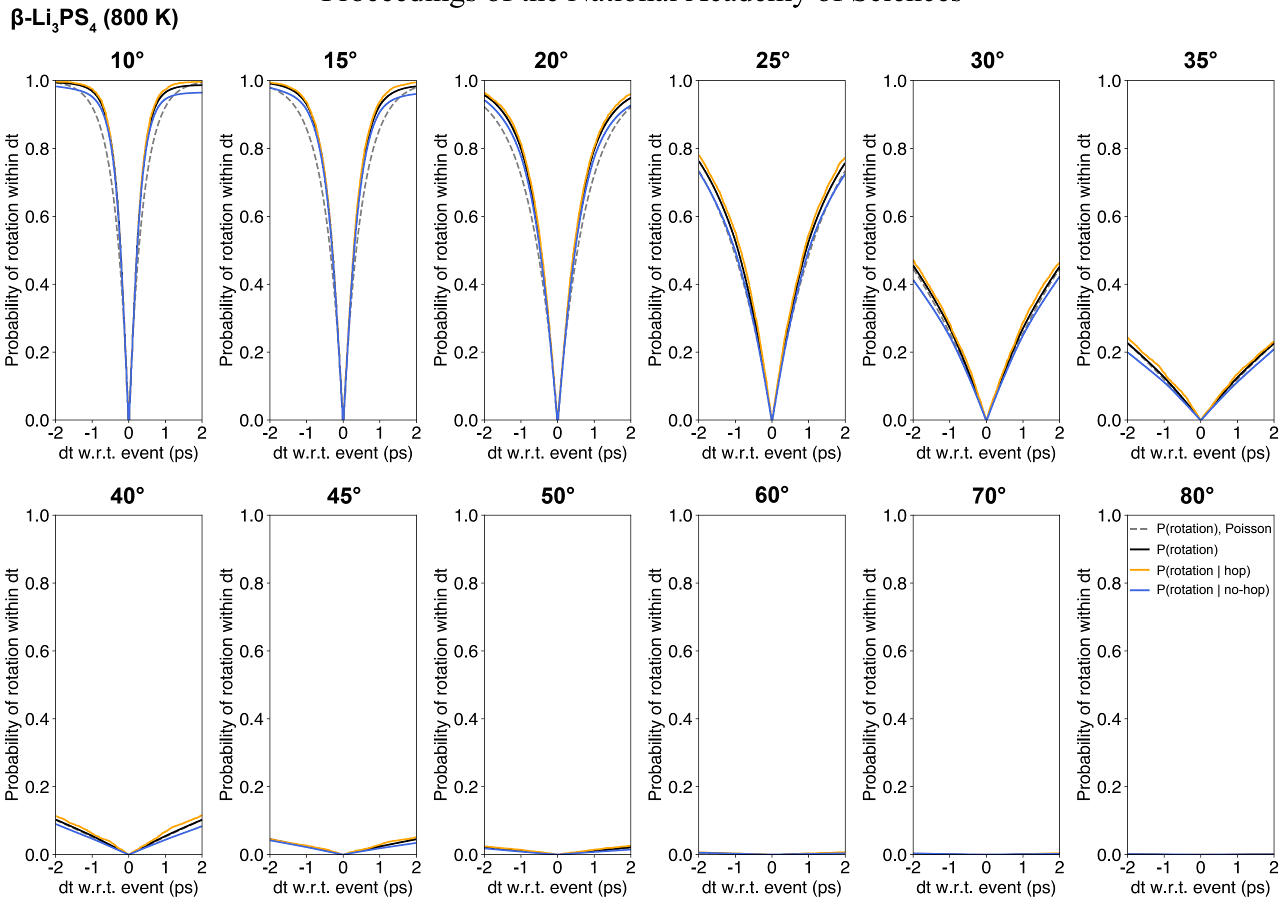

Figure S27. Full probability analysis of β-Li_3PS_4 at 800 K for rotational motions from 10° to 80°. Orange lines plot the conditional probability of a rotation event of cutoff angle θ given that a lithium-hop occurs at dt = 0. Blue lines plot the conditional probability of a rotation event of cutoff angle θ given that no lithium-hop occurs within ± 2 ps range of dt = 0. Black lines plot the probability of a rotation event of cutoff angle θ regardless of lithium-hops. Dashed grey lines show the probability of a single PS_4 rotation event of cutoff angle θ occurring within ± dt assuming that rotation events follow a Poisson process.

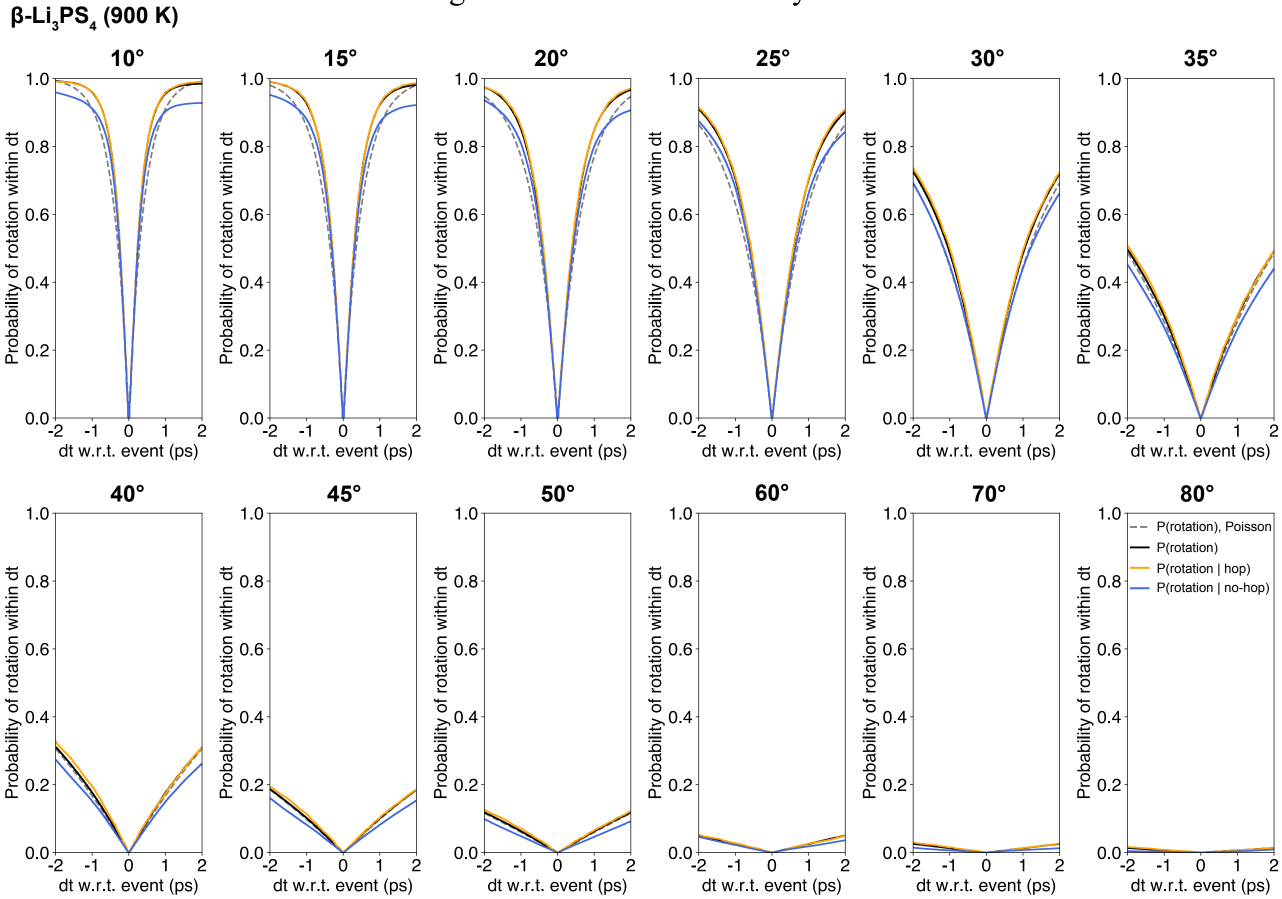

Figure S28. Full probability analysis of β-Li_3PS_4 at 900 K for rotational motions from 10° to 80°. Orange lines plot the conditional probability of a rotation event of cutoff angle θ given that a lithium-hop occurs at dt = 0. Blue lines plot the conditional probability of a rotation event of cutoff angle θ given that no lithium-hop occurs within ± 2 ps range of dt = 0. Black lines plot the probability of a rotation event of cutoff angle θ regardless of lithium-hops. Dashed grey lines show the probability of a single PS_4 rotation event of cutoff angle θ occurring within ± dt assuming that rotation events follow a Poisson process.

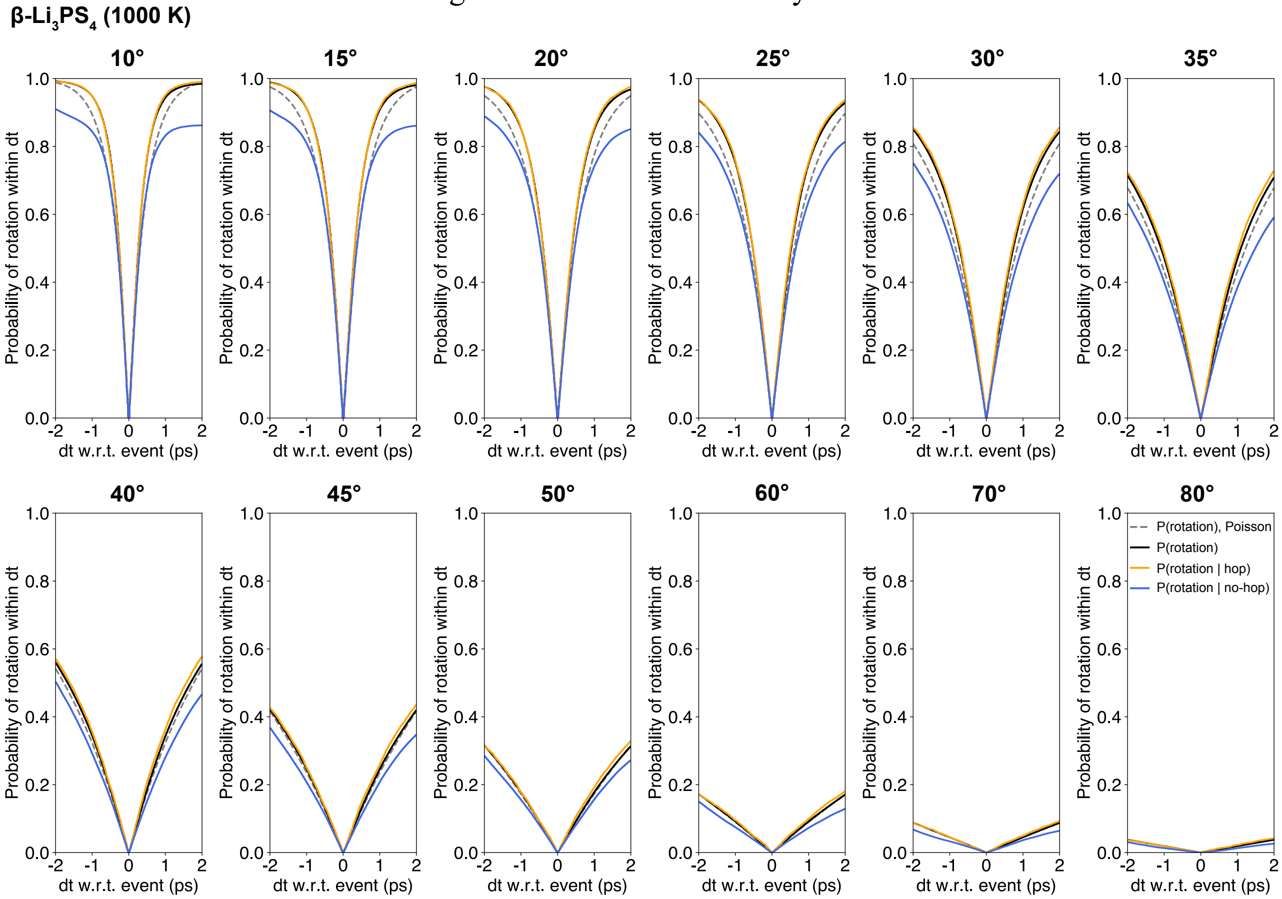

Figure S29. Full probability analysis of β-Li_3PS_4 at 1000 K for rotational motions from 10° to 80°. Orange lines plot the conditional probability of a rotation event of cutoff angle θ given that a lithium-hop occurs at dt = 0. Blue lines plot the conditional probability of a rotation event of cutoff angle θ given that no lithium-hop occurs within ± 2 ps range of dt = 0. Black lines plot the probability of a rotation event of cutoff angle θ regardless of lithium-hops. Dashed grey lines show the probability of a single PS_4 rotation event of cutoff angle θ occurring within ± dt assuming that rotation events follow a Poisson process.

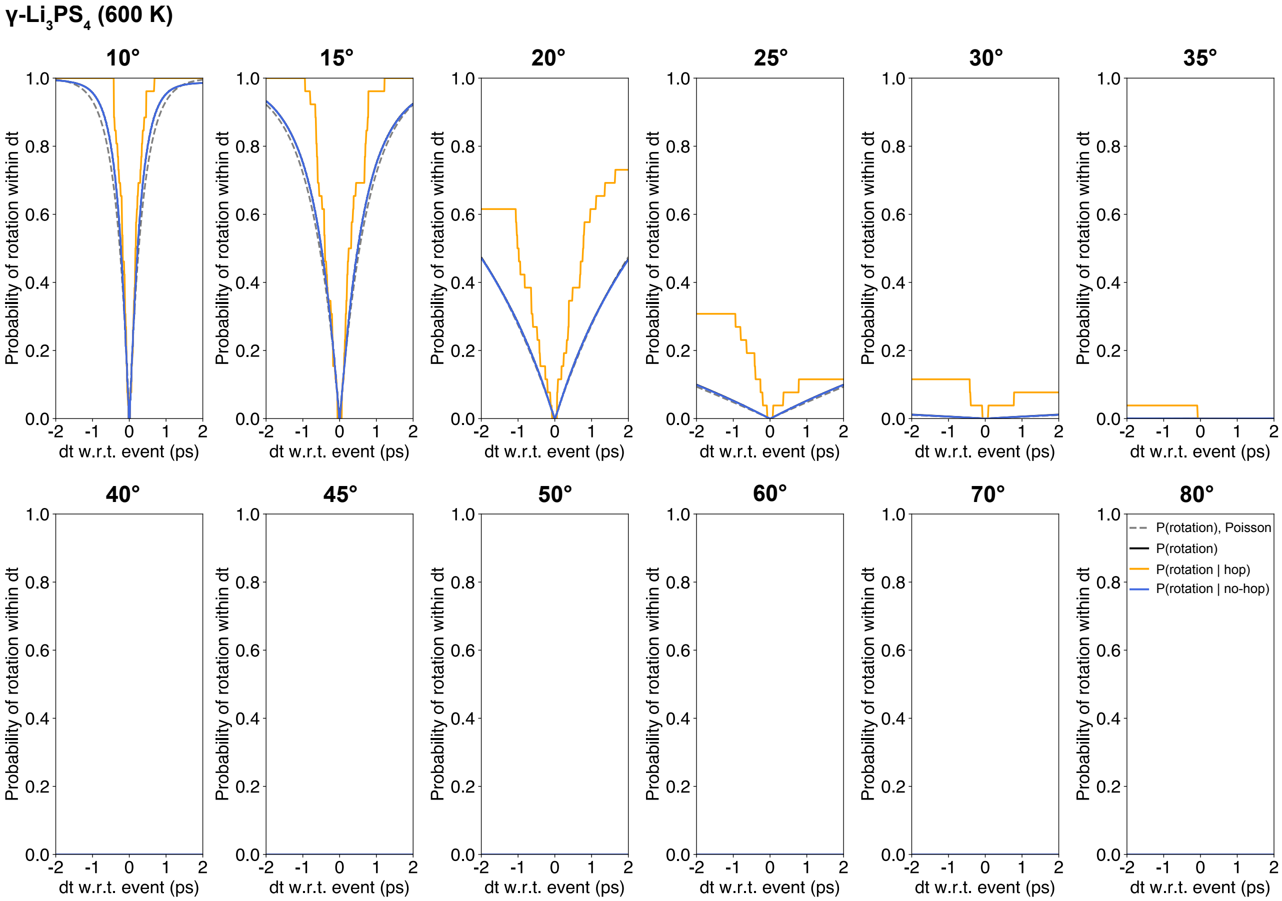

Figure S30. Full probability analysis of γ-Li_3PS_4 at 600 K for rotational motions from 10° to 80°. Orange lines plot the conditional probability of a rotation event of cutoff angle θ given that a lithium-hop occurs at dt = 0. Blue lines plot the conditional probability of a rotation event of cutoff angle θ given that no lithium-hop occurs within ± 2 ps range of dt = 0. Black lines plot the probability of a rotation event of cutoff angle θ regardless of lithium-hops. Dashed grey lines show the probability of a single PS_4 rotation event of cutoff angle θ occurring within ± dt assuming that rotation events follow a Poisson process.

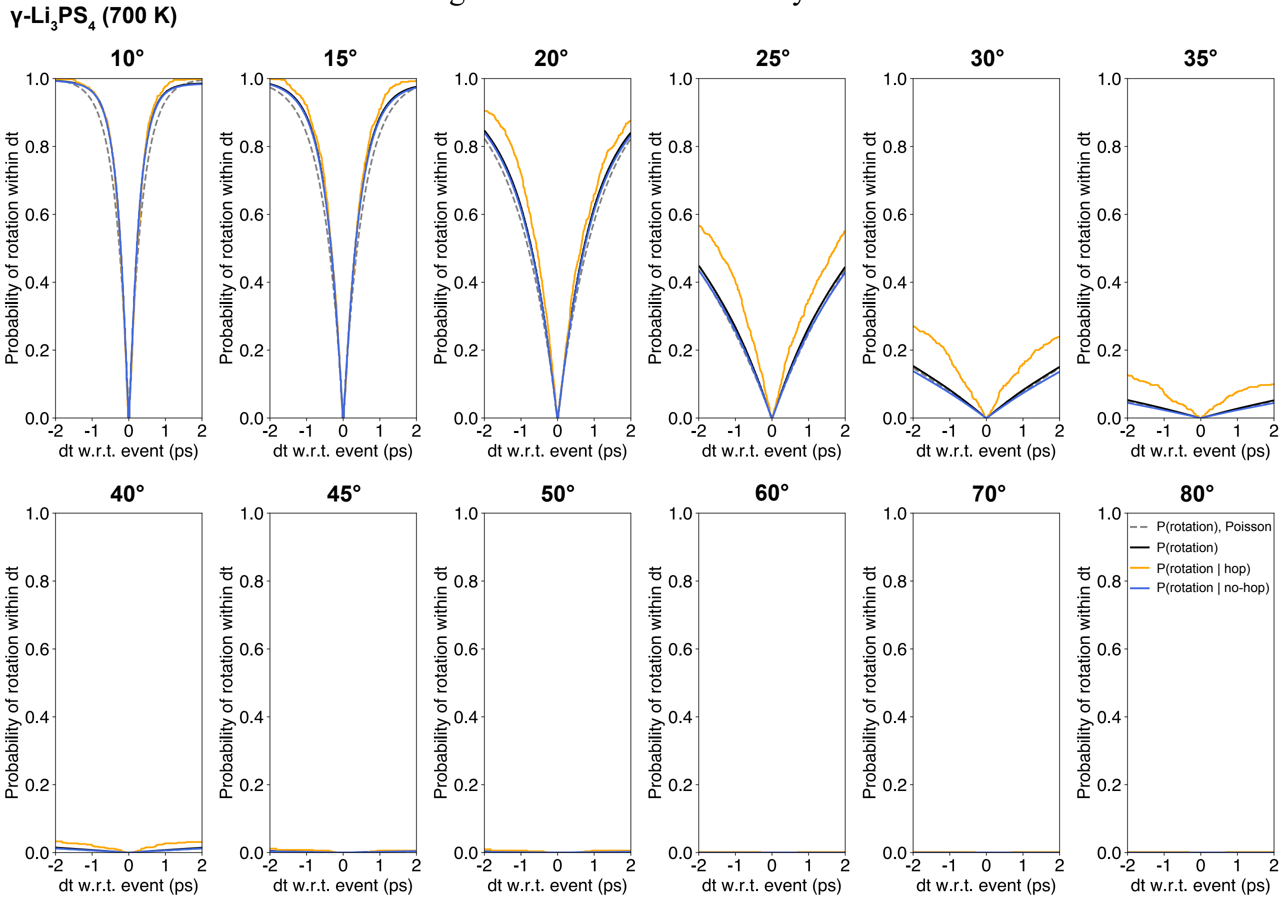

Figure S31. Full probability analysis of γ-Li_3PS_4 at 700 K for rotational motions from 10° to 80°. Orange lines plot the conditional probability of a rotation event of cutoff angle θ given that a lithium-hop occurs at dt = 0. Blue lines plot the conditional probability of a rotation event of cutoff angle θ given that no lithium-hop occurs within ± 2 ps range of dt = 0. Black lines plot the probability of a rotation event of cutoff angle θ regardless of lithium-hops. Dashed grey lines show the probability of a single PS_4 rotation event of cutoff angle θ occurring within ± dt assuming that rotation events follow a Poisson process.

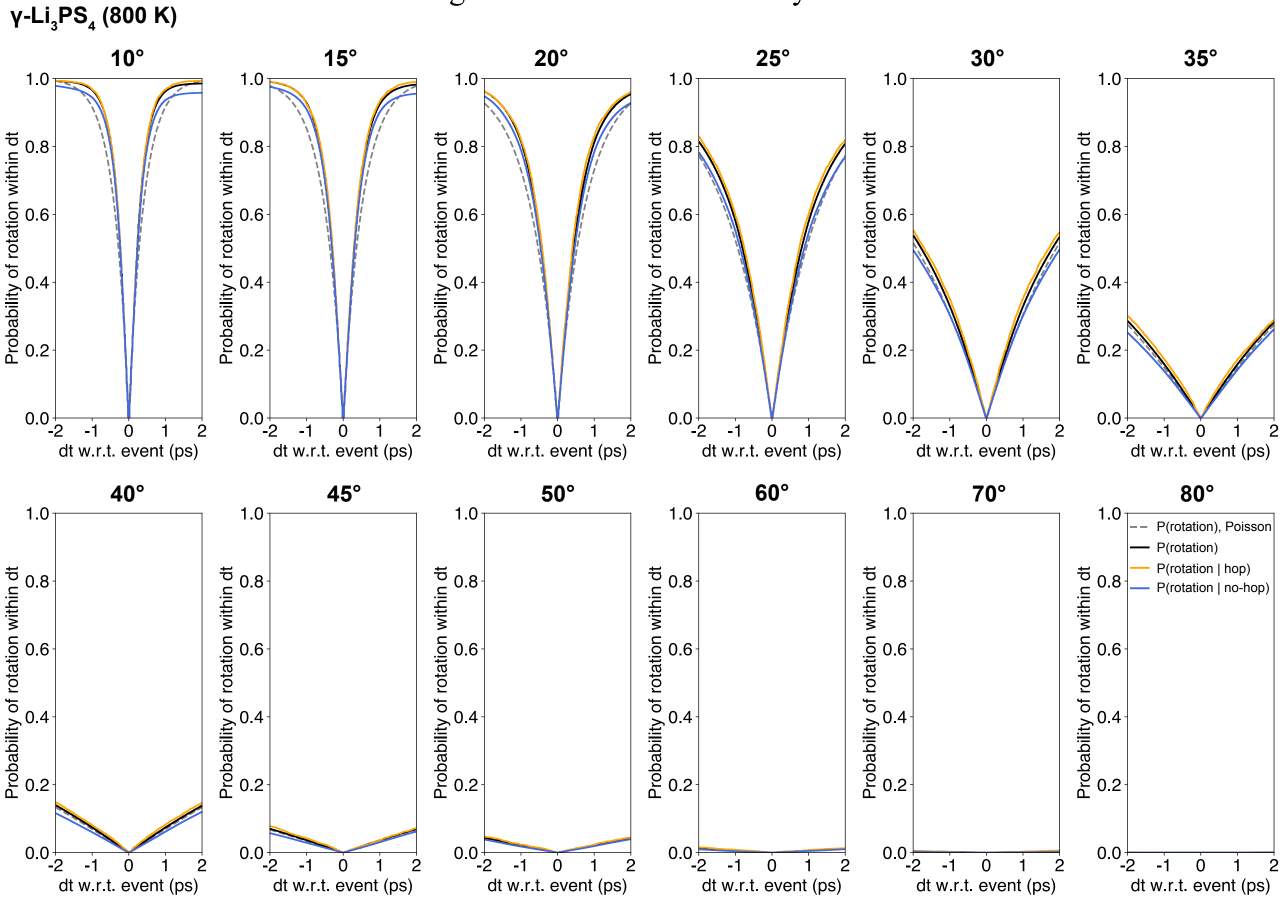

Figure S32. Full probability analysis of γ-Li_3PS_4 at 800 K for rotational motions from 10° to 80°. Orange lines plot the conditional probability of a rotation event of cutoff angle θ given that a lithium-hop occurs at dt = 0. Blue lines plot the conditional probability of a rotation event of cutoff angle θ given that no lithium-hop occurs within ± 2 ps range of dt = 0. Black lines plot the probability of a rotation event of cutoff angle θ regardless of lithium-hops. Dashed grey lines show the probability of a single PS_4 rotation event of cutoff angle θ occurring within ± dt assuming that rotation events follow a Poisson process.

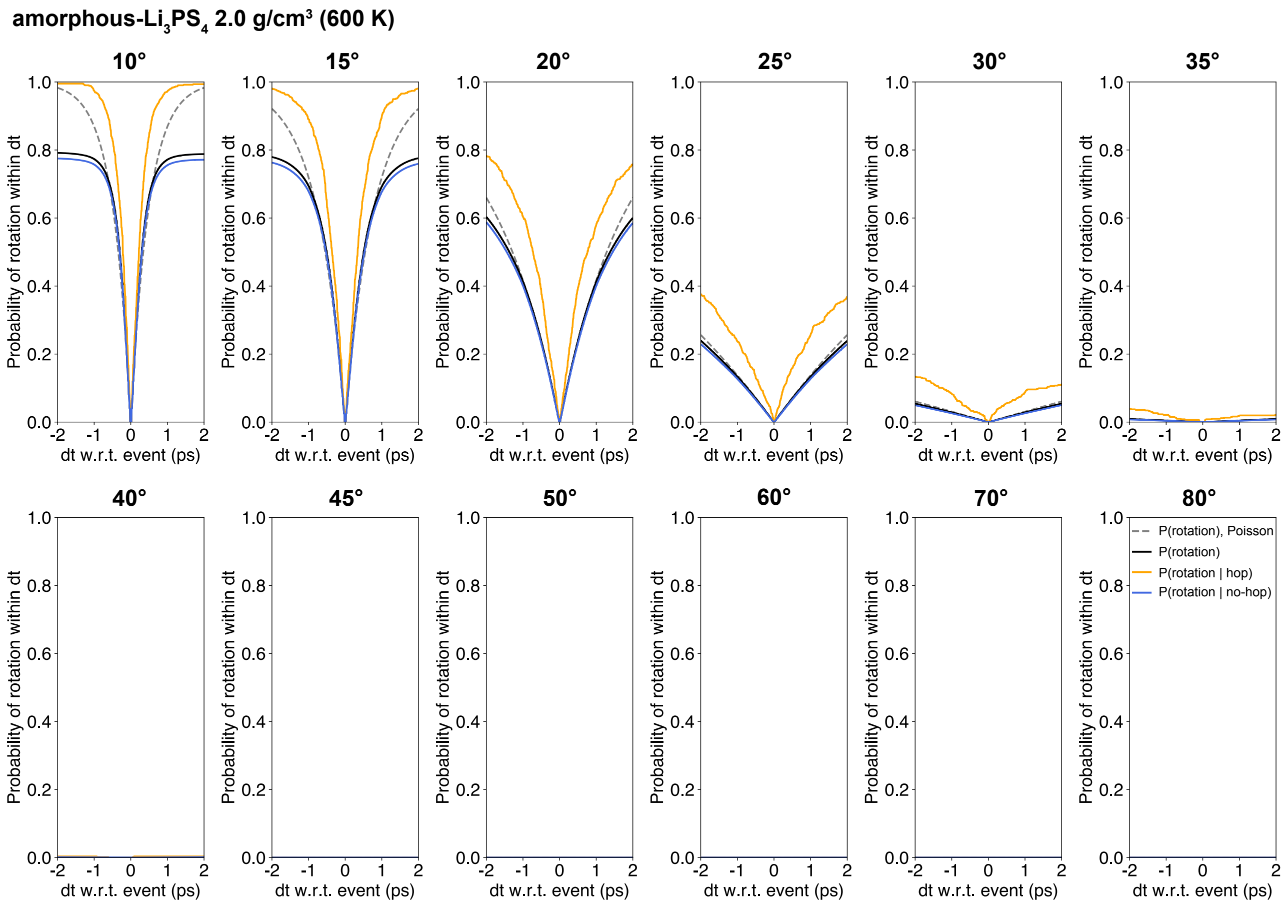

Figure S33. Full probability analysis of amorphous Li_3PS_4 (2.0 g/cm^3) at 600 K for rotational motions from 10° to 80°. Orange lines plot the conditional probability of a rotation event of cutoff angle θ given that a lithium-hop occurs at dt = 0. Blue lines plot the conditional probability of a rotation event of cutoff angle θ given that no lithium-hop occurs within ± 2 ps range of dt = 0. Black lines plot the probability of a rotation event of cutoff angle θ regardless of lithium-hops. Dashed grey lines show the probability of a single PS_4 rotation event of cutoff angle θ occurring within ± dt assuming that rotation events follow a Poisson process.

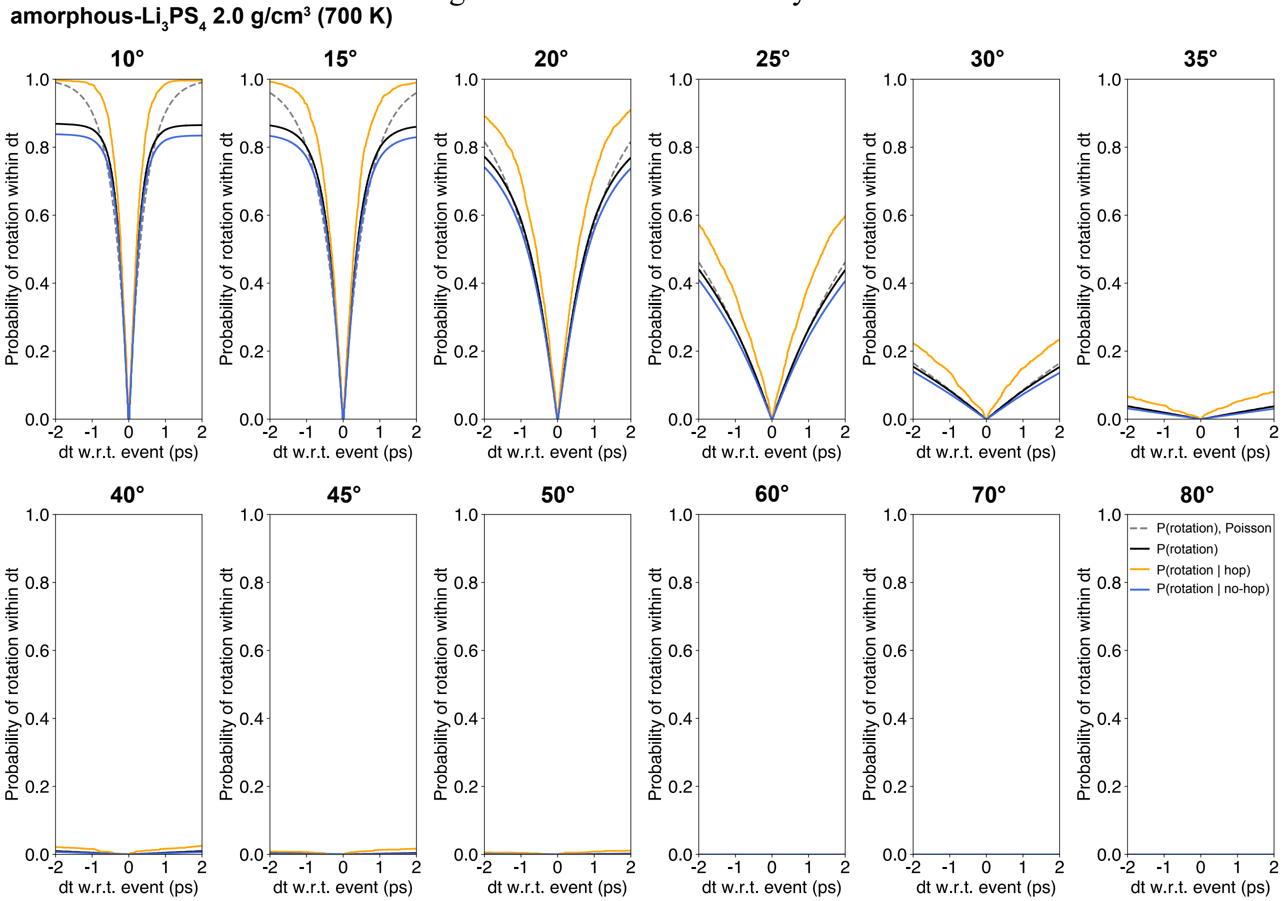

Figure S34. Full probability analysis of amorphous Li_3PS_4 (2.0 g/cm^3) at 700 K for rotational motions from 10° to 80°. Orange lines plot the conditional probability of a rotation event of cutoff angle θ given that a lithium-hop occurs at dt = 0. Blue lines plot the conditional probability of a rotation event of cutoff angle θ given that no lithium-hop occurs within ± 2 ps range of dt = 0. Black lines plot the probability of a rotation event of cutoff angle θ regardless of lithium-hops. Dashed grey lines show the probability of a single PS_4 rotation event of cutoff angle θ occurring within ± dt assuming that rotation events follow a Poisson process.

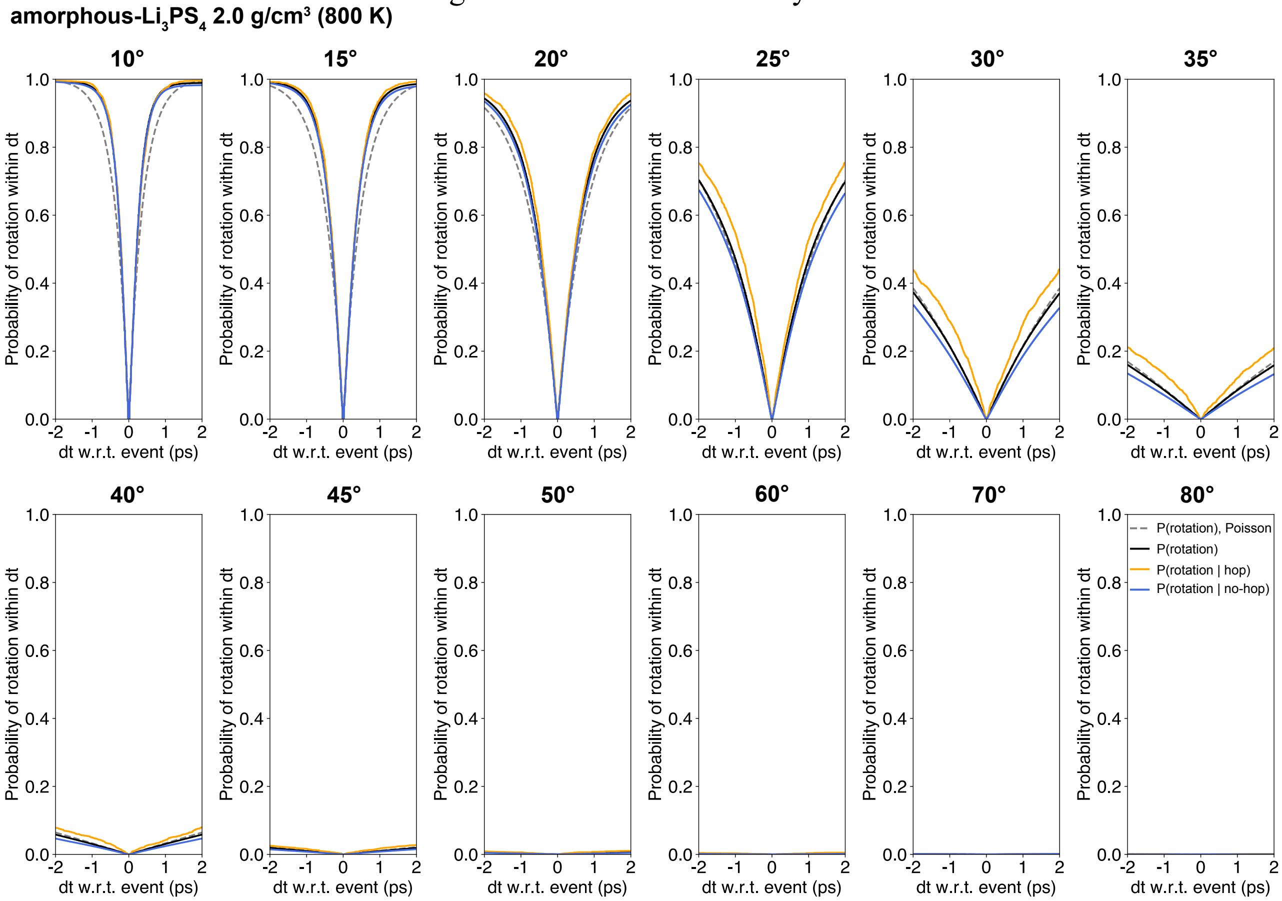

Figure S35. Full probability analysis of amorphous Li_3PS_4 (2.0 g/cm^3) at 800 K for rotational motions from 10° to 80°. Orange lines plot the conditional probability of a rotation event of cutoff angle θ given that a lithium-hop occurs at dt = 0. Blue lines plot the conditional probability of a rotation event of cutoff angle θ given that no lithium-hop occurs within ± 2 ps range of dt = 0. Black lines plot the probability of a rotation event of cutoff angle θ regardless of lithium-hops. Dashed grey lines show the probability of a single PS_4 rotation event of cutoff angle θ occurring within ± dt assuming that rotation events follow a Poisson process.

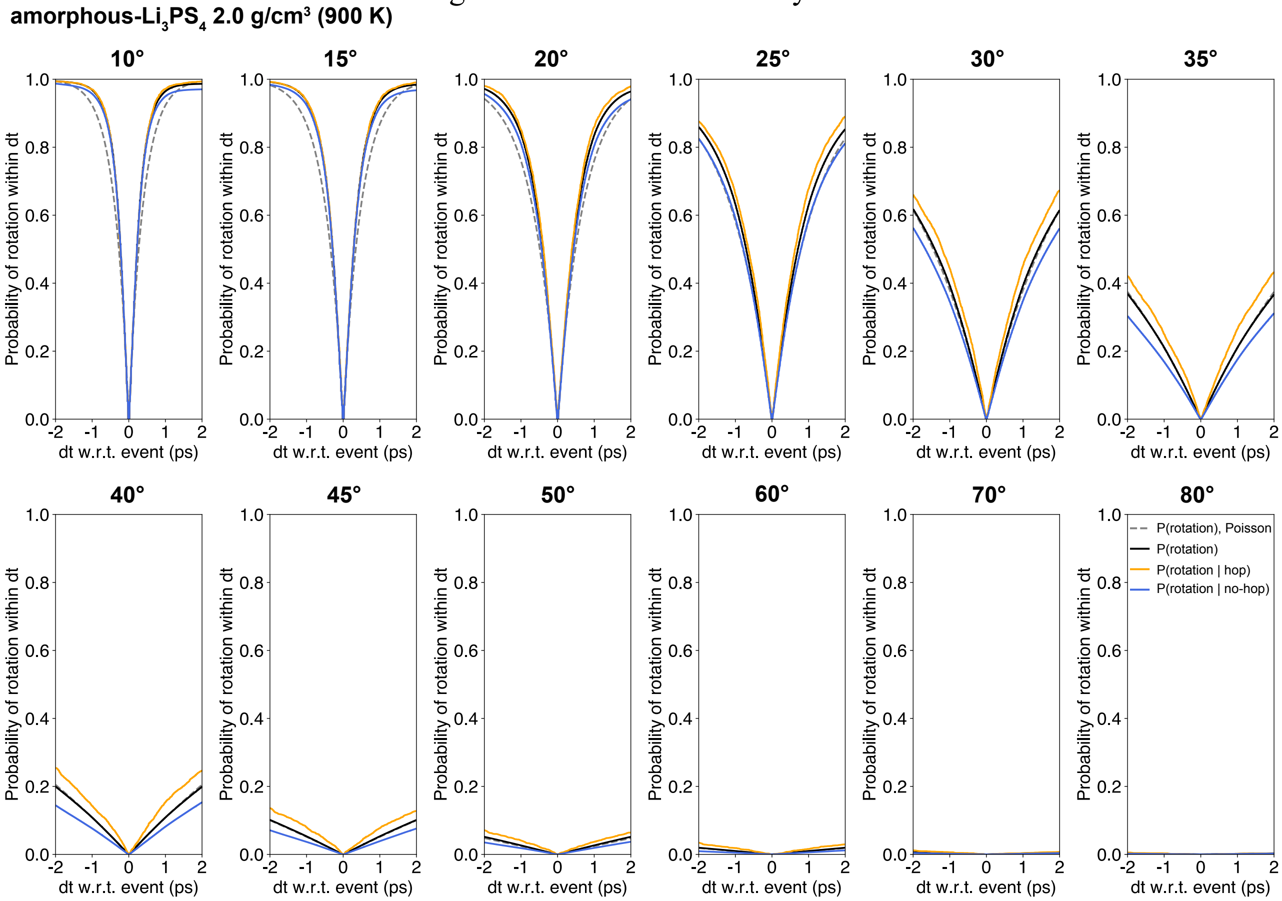

Figure S36. Full probability analysis of amorphous Li_3PS_4 (2.0 g/cm^3) at 900 K for rotational motions from 10° to 80°. Orange lines plot the conditional probability of a rotation event of cutoff angle θ given that a lithium-hop occurs at dt = 0. Blue lines plot the conditional probability of a rotation event of cutoff angle θ given that no lithium-hop occurs within ± 2 ps range of dt = 0. Black lines plot the probability of a rotation event of cutoff angle θ regardless of lithium-hops. Dashed grey lines show the probability of a single PS_4 rotation event of cutoff angle θ occurring within ± dt assuming that rotation events follow a Poisson process.

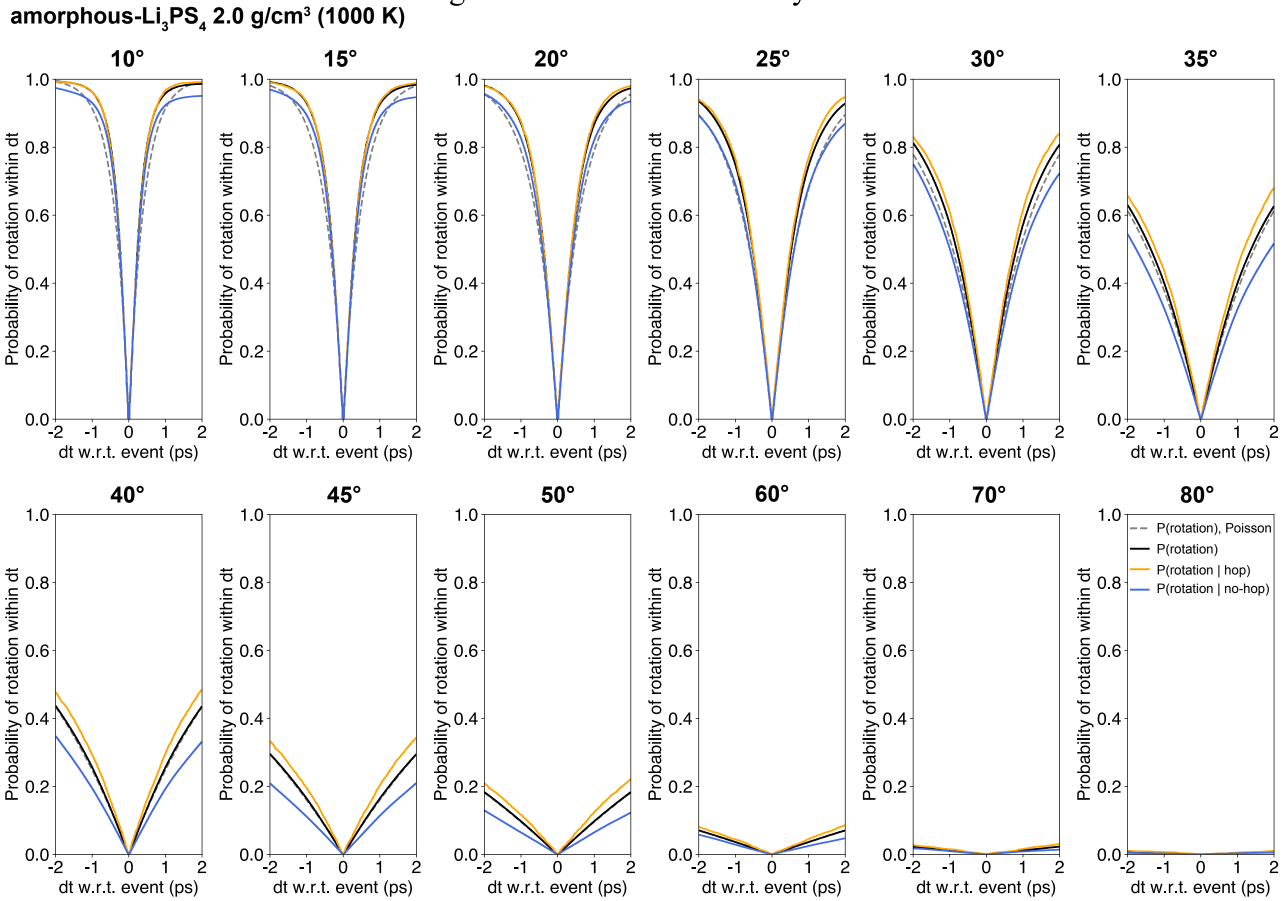

Figure S37. Full probability analysis of amorphous Li_3PS_4 (2.0 g/cm^3) at 1000 K for rotational motions from 10° to 80°. Orange lines plot the conditional probability of a rotation event of cutoff angle θ given that a lithium-hop occurs at dt = 0. Blue lines plot the conditional probability of a rotation event of cutoff angle θ given that no lithium-hop occurs within ± 2 ps range of dt = 0. Black lines plot the probability of a rotation event of cutoff angle θ regardless of lithium-hops. Dashed grey lines show the probability of a single PS_4 rotation event of cutoff angle θ occurring within ± dt assuming that rotation events follow a Poisson process.

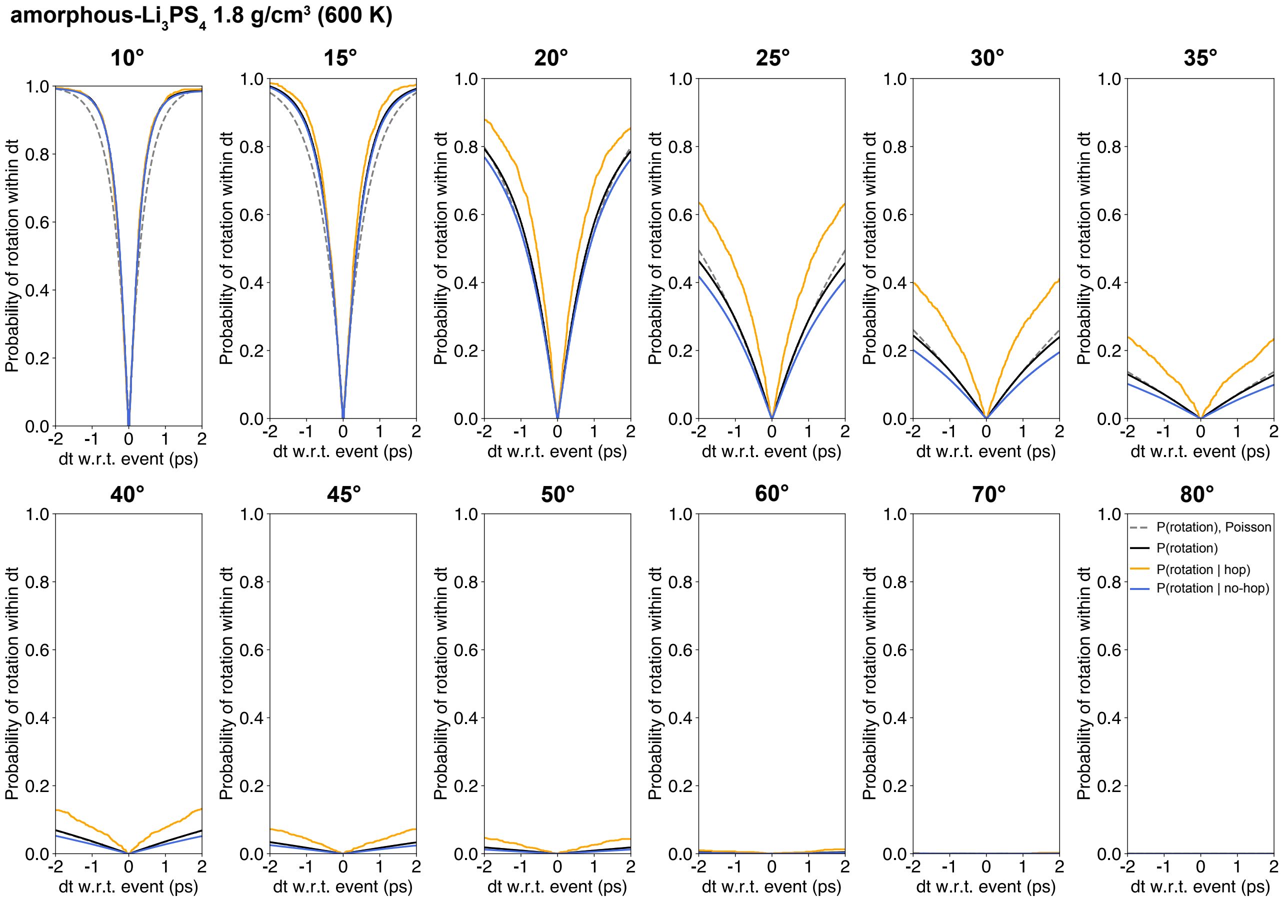

Figure S38. Full probability analysis of amorphous Li_3PS_4 (1.8 g/cm^3) at 600 K for rotational motions from 10° to 80°. Orange lines plot the conditional probability of a rotation event of cutoff angle θ given that a lithium-hop occurs at dt = 0. Blue lines plot the conditional probability of a rotation event of cutoff angle θ given that no lithium-hop occurs within ± 2 ps range of dt = 0. Black lines plot the probability of a rotation event of cutoff angle θ regardless of lithium-hops. Dashed grey lines show the probability of a single PS_4 rotation event of cutoff angle θ occurring within ± dt assuming that rotation events follow a Poisson process.

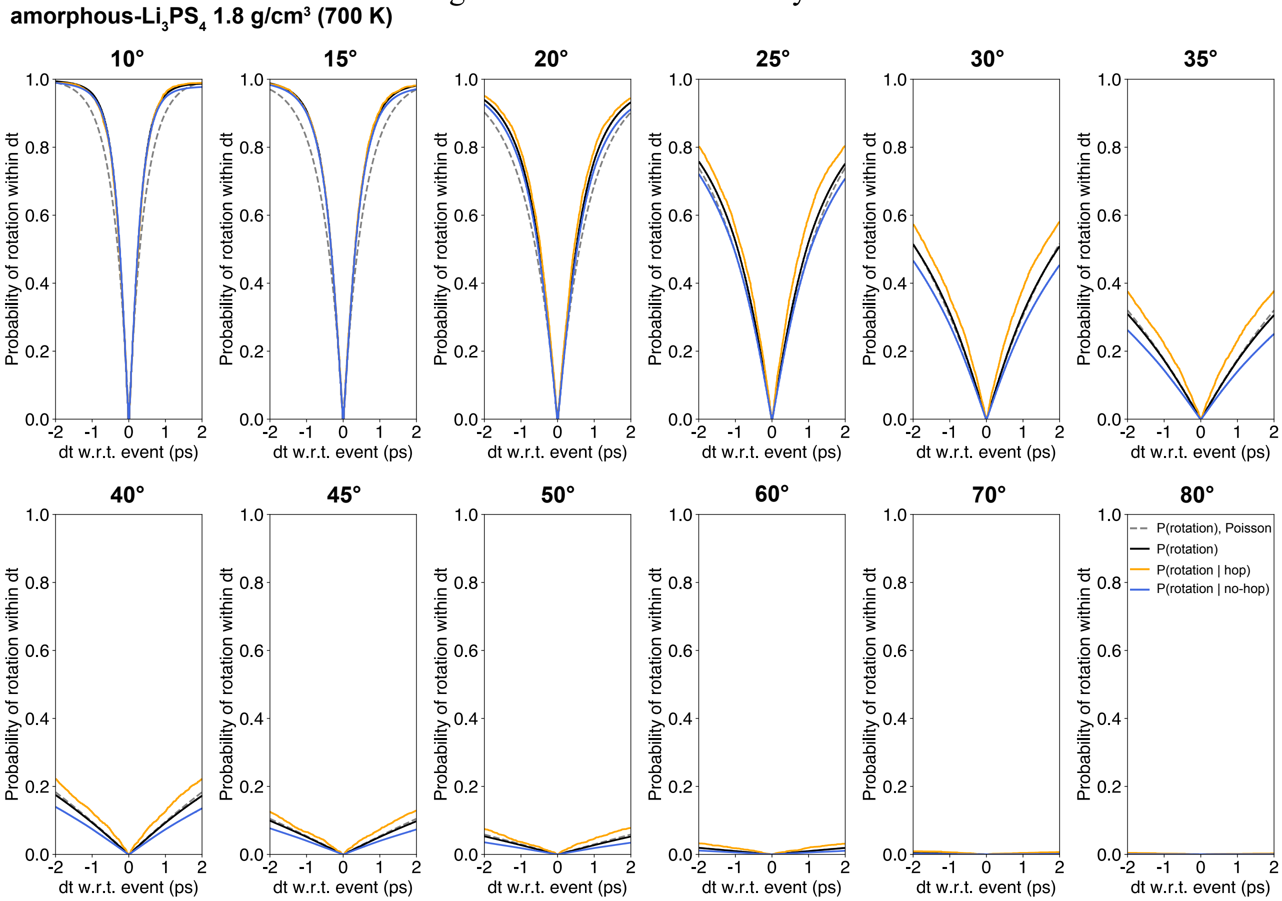

Figure S39. Full probability analysis of amorphous Li_3PS_4 (1.8 g/cm^3) at 700 K for rotational motions from 10° to 80°. Orange lines plot the conditional probability of a rotation event of cutoff angle θ given that a lithium-hop occurs at dt = 0. Blue lines plot the conditional probability of a rotation event of cutoff angle θ given that no lithium-hop occurs within ± 2 ps range of dt = 0. Black lines plot the probability of a rotation event of cutoff angle θ regardless of lithium-hops. Dashed grey lines show the probability of a single PS_4 rotation event of cutoff angle θ occurring within ± dt assuming that rotation events follow a Poisson process.

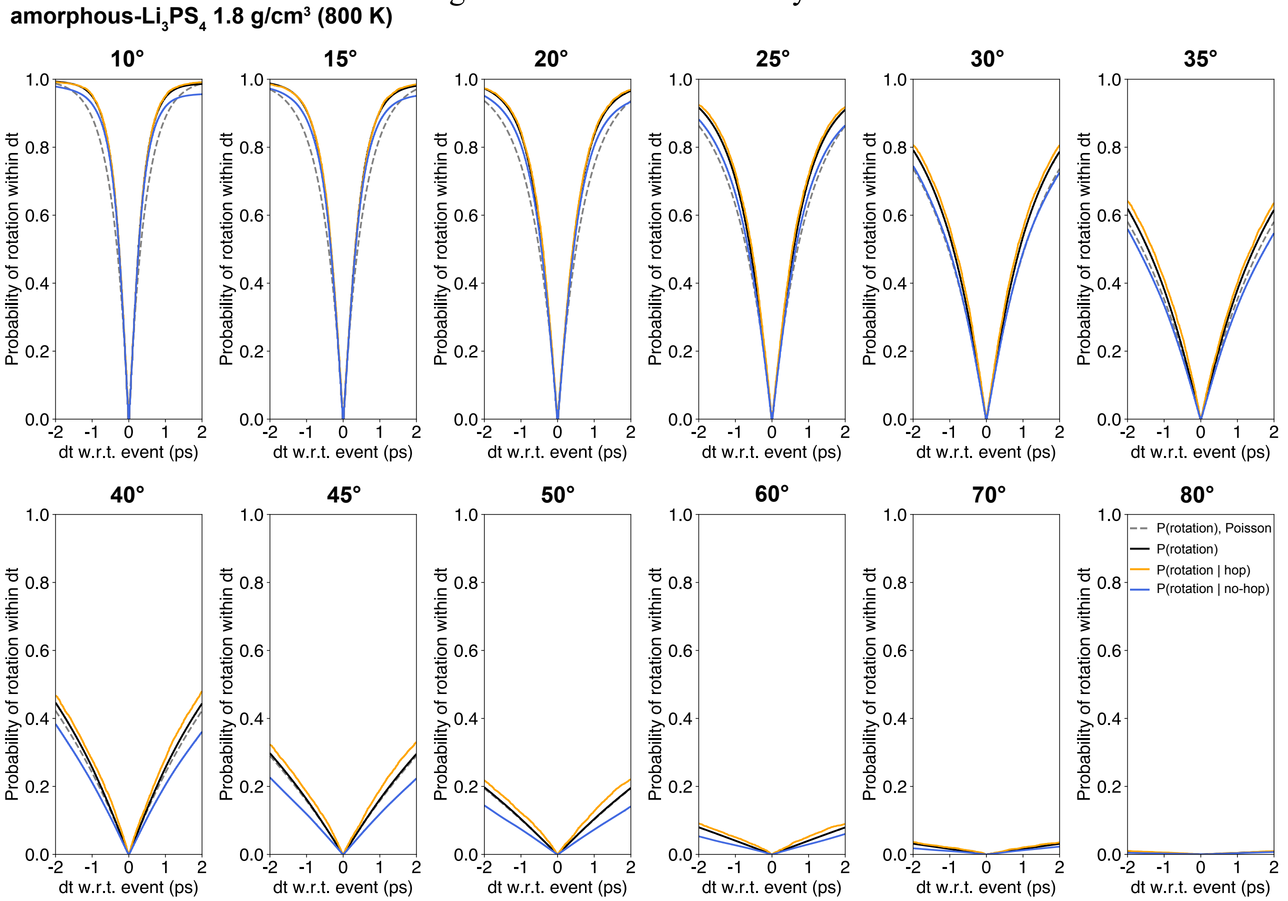

Figure S40. Full probability analysis of amorphous Li_3PS_4 (1.8 g/cm^3) at 800 K for rotational motions from 10° to 80°. Orange lines plot the conditional probability of a rotation event of cutoff angle θ given that a lithium-hop occurs at dt = 0. Blue lines plot the conditional probability of a rotation event of cutoff angle θ given that no lithium-hop occurs within ± 2 ps range of dt = 0. Black lines plot the probability of a rotation event of cutoff angle θ regardless of lithium-hops. Dashed grey lines show the probability of a single PS_4 rotation event of cutoff angle θ occurring within ± dt assuming that rotation events follow a Poisson process.

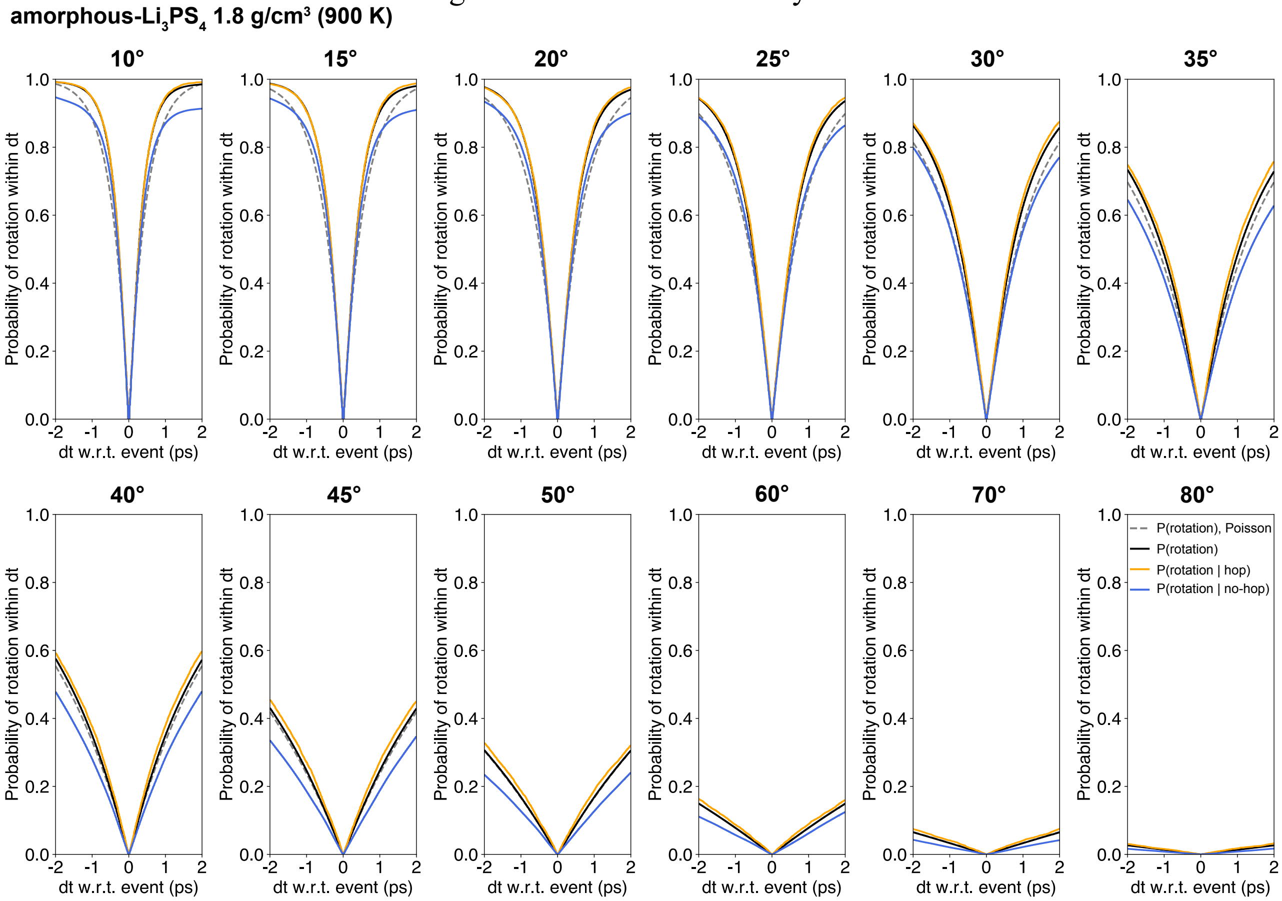

Figure S41. Full probability analysis of amorphous Li_3PS_4 (1.8 g/cm^3) at 900 K for rotational motions from 10° to 80°. Orange lines plot the conditional probability of a rotation event of cutoff angle θ given that a lithium-hop occurs at dt = 0. Blue lines plot the conditional probability of a rotation event of cutoff angle θ given that no lithium-hop occurs within ± 2 ps range of dt = 0. Black lines plot the probability of a rotation event of cutoff angle θ regardless of lithium-hops. Dashed grey lines show the probability of a single PS_4 rotation event of cutoff angle θ occurring within ± dt assuming that rotation events follow a Poisson process.

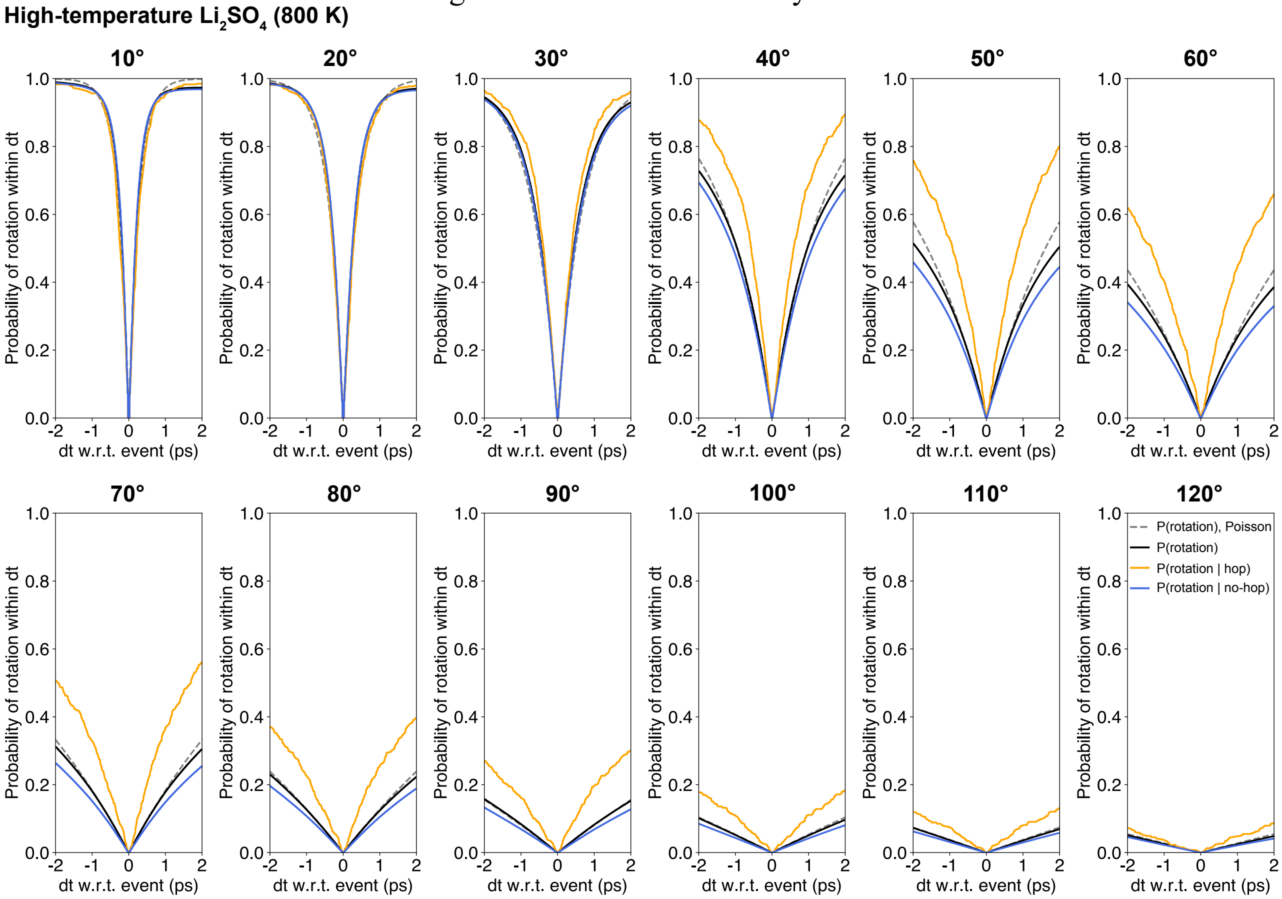

Figure S42. Full probability analysis of HT-Li_2SO_4 at 800 K for rotational motions from 10° to 80°. Orange lines plot the conditional probability of a rotation event of cutoff angle θ given that a lithium-hop occurs at dt = 0. Blue lines plot the conditional probability of a rotation event of cutoff angle θ given that no lithium-hop occurs within ± 2 ps range of dt = 0. Black lines plot the probability of a rotation event of cutoff angle θ regardless of lithium-hops. Dashed grey lines show the probability of a single PS_4 rotation event of cutoff angle θ occurring within ± dt assuming that rotation events follow a Poisson process.

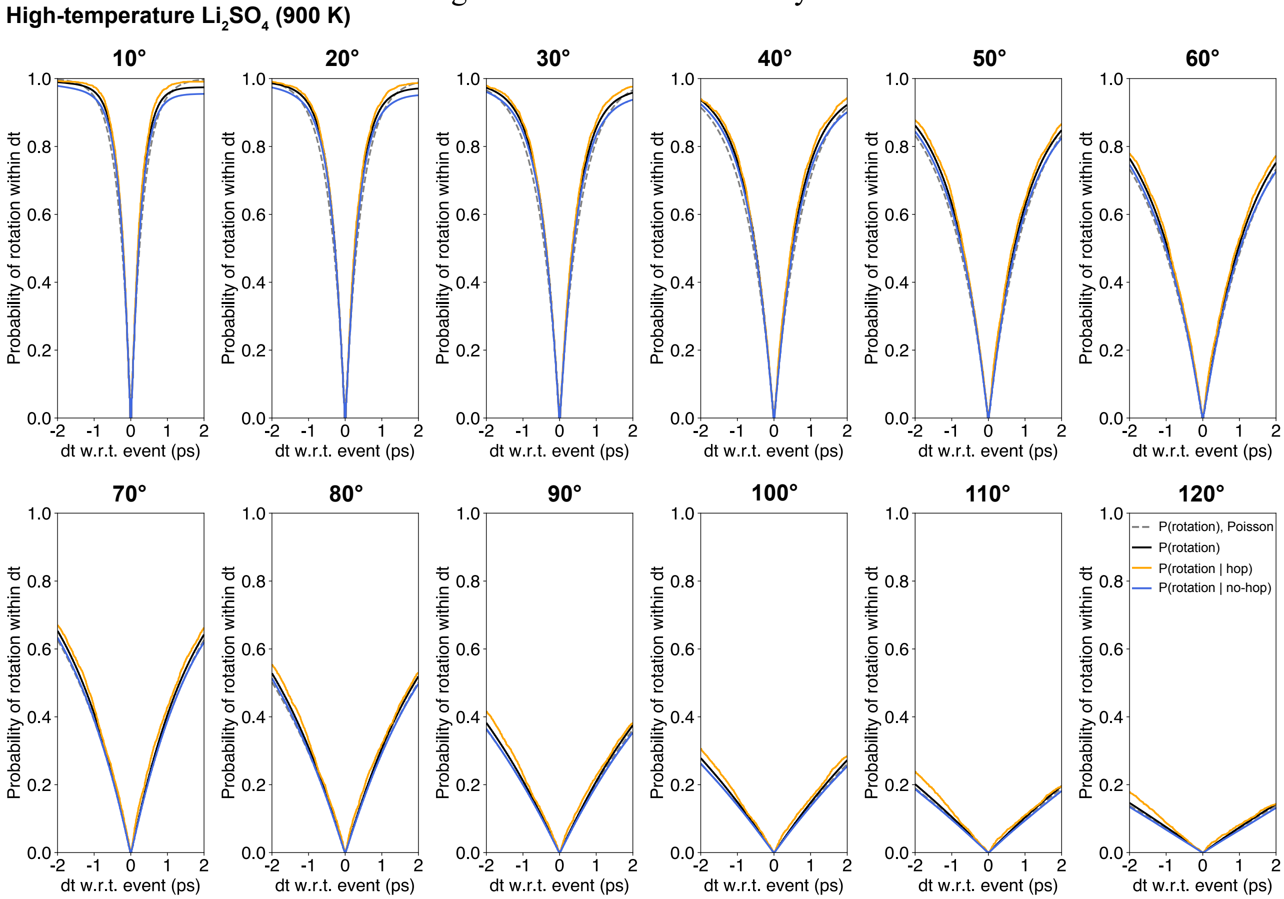

Figure S43. Full probability analysis of HT-Li_2SO_4 at 900 K for rotational motions from 10° to 80°. Orange lines plot the conditional probability of a rotation event of cutoff angle θ given that a lithium-hop occurs at dt = 0. Blue lines plot the conditional probability of a rotation event of cutoff angle θ given that no lithium-hop occurs within ± 2 ps range of dt = 0. Black lines plot the probability of a rotation event of cutoff angle θ regardless of lithium-hops. Dashed grey lines show the probability of a single PS_4 rotation event of cutoff angle θ occurring within ± dt assuming that rotation events follow a Poisson process.

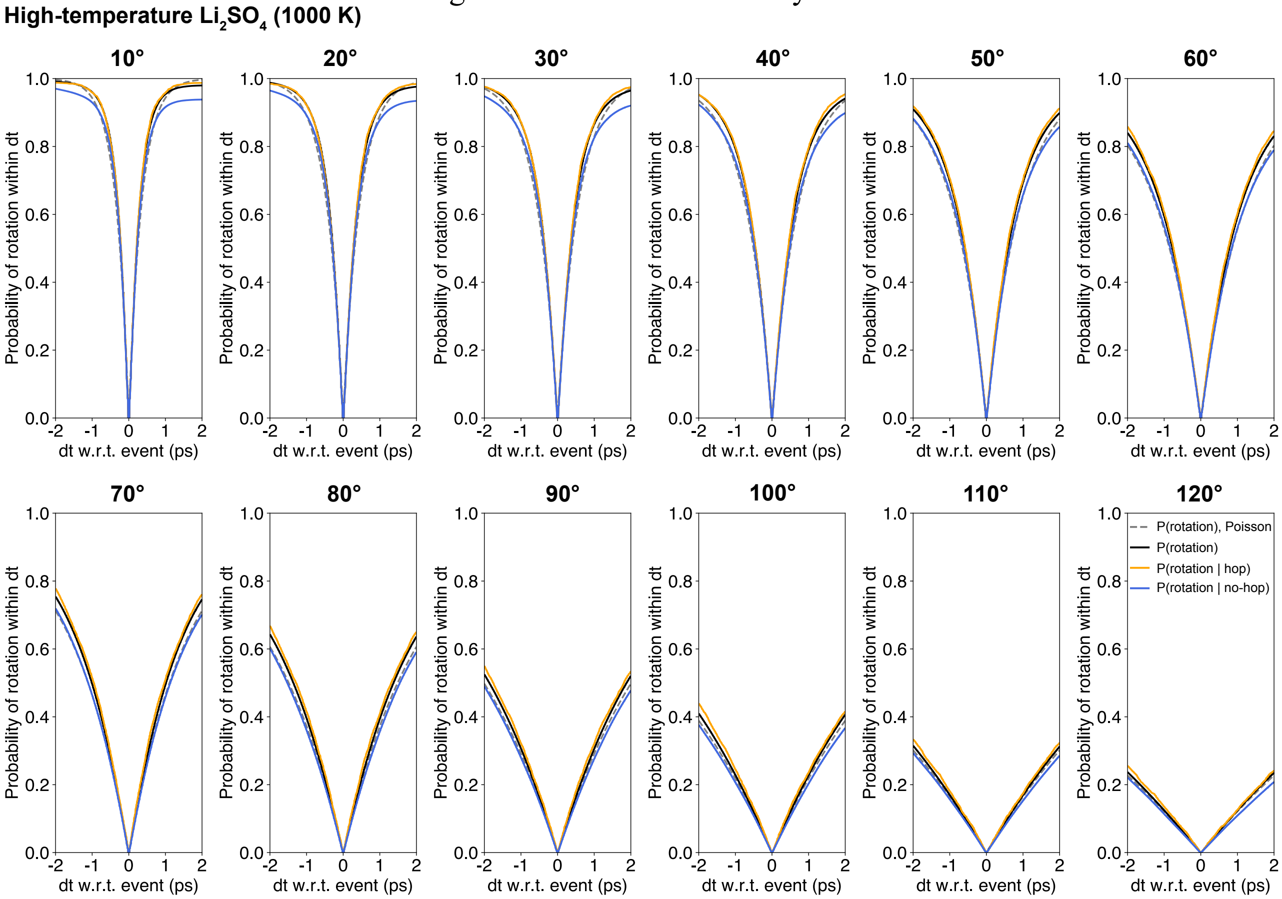

Figure S44. Full probability analysis of HT-Li_2SO_4 at 1000 K for rotational motions from 10° to 80°. Orange lines plot the conditional probability of a rotation event of cutoff angle θ given that a lithium-hop occurs at dt = 0. Blue lines plot the conditional probability of a rotation event of cutoff angle θ given that no lithium-hop occurs within ± 2 ps range of dt = 0. Black lines plot the probability of a rotation event of cutoff angle θ regardless of lithium-hops. Dashed grey lines show the probability of a single PS_4 rotation event of cutoff angle θ occurring within ± dt assuming that rotation events follow a Poisson process.

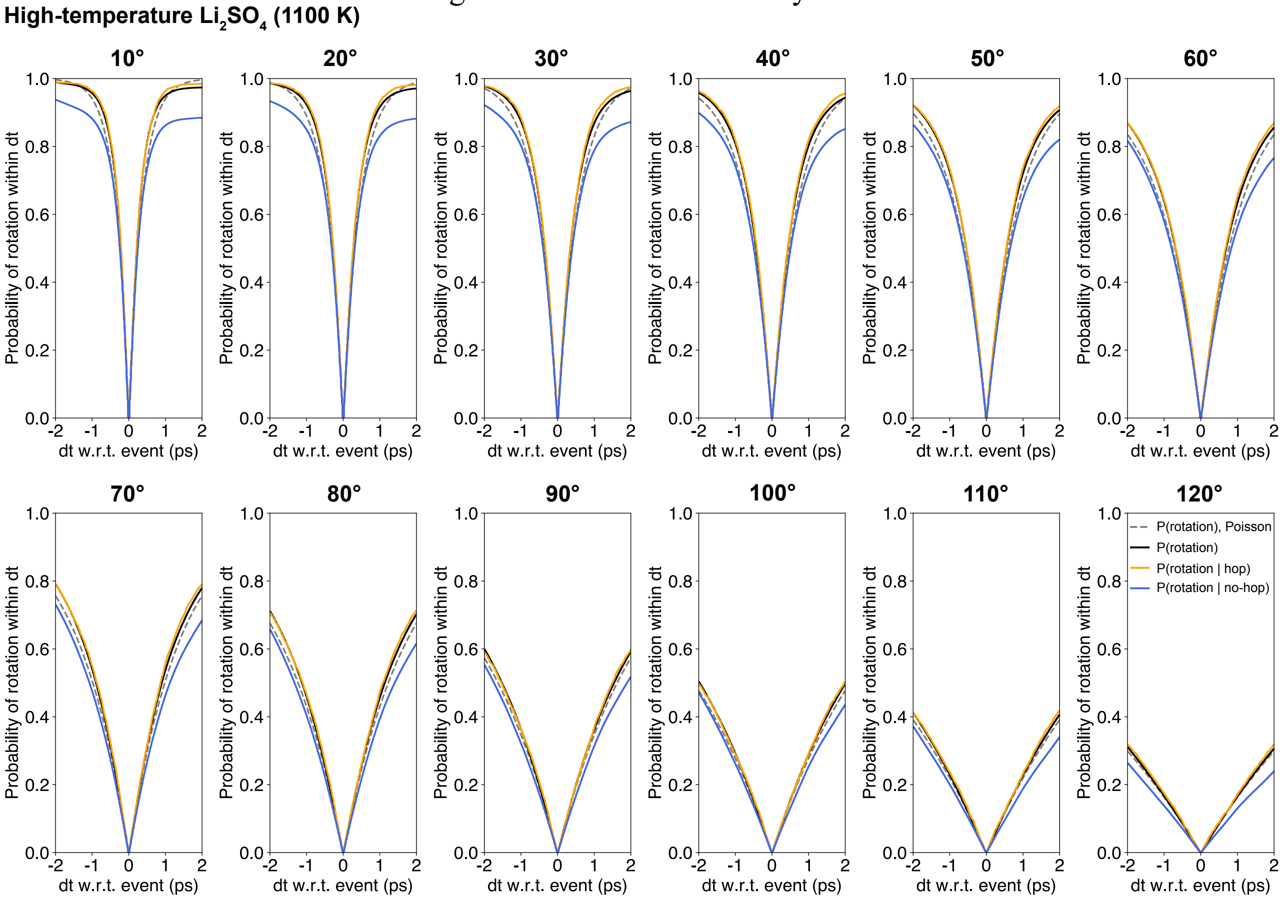

Figure S45. Full probability analysis of HT-Li_2SO_4 at 1100 K for rotational motions from 10° to 80°. Orange lines plot the conditional probability of a rotation event of cutoff angle θ given that a lithium-hop occurs at dt = 0. Blue lines plot the conditional probability of a rotation event of cutoff angle θ given that no lithium-hop occurs within ± 2 ps range of dt = 0. Black lines plot the probability of a rotation event of cutoff angle θ regardless of lithium-hops. Dashed grey lines show the probability of a single PS_4 rotation event of cutoff angle θ occurring within ± dt assuming that rotation events follow a Poisson process.

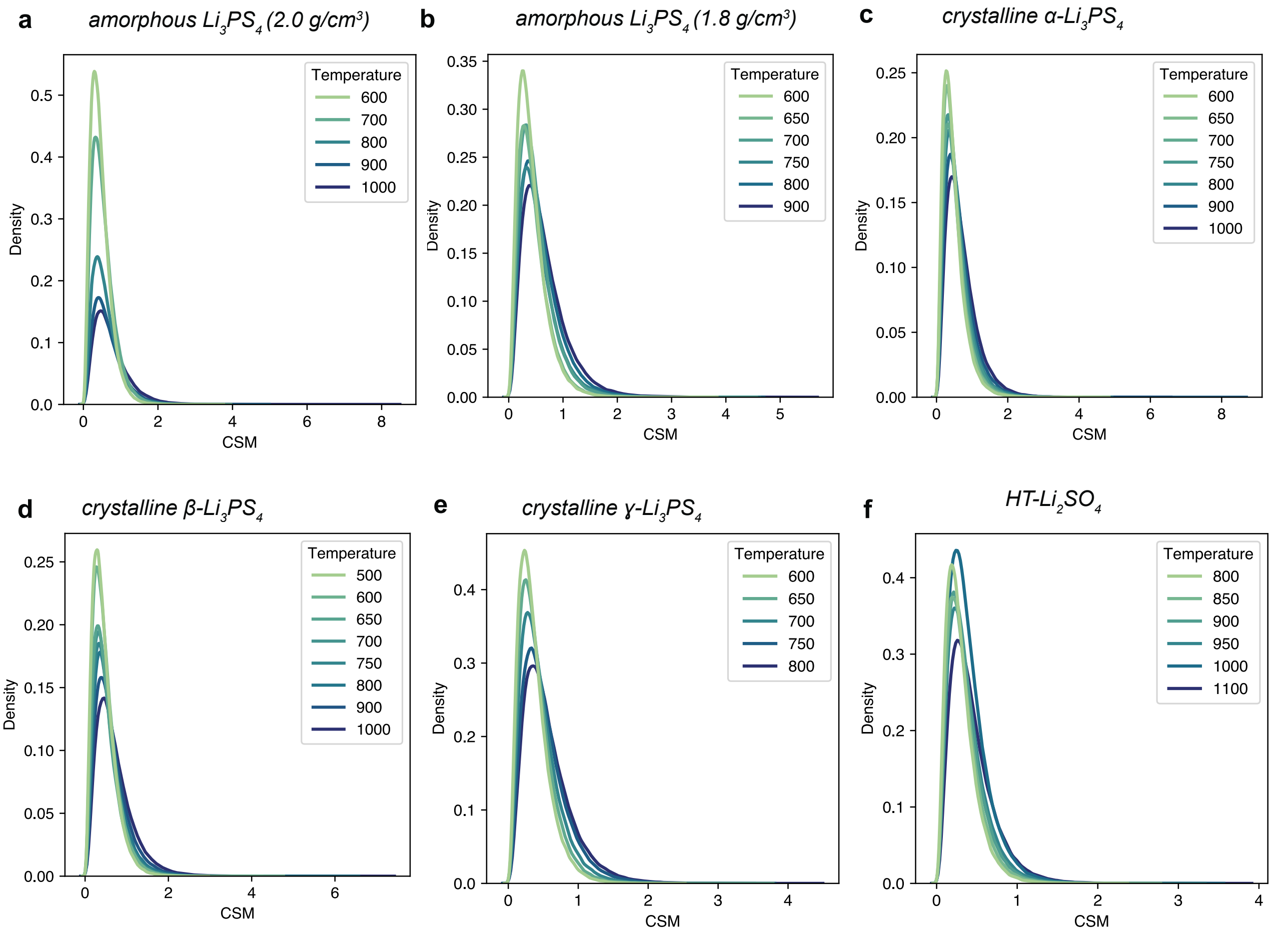

Figure S46. Distribution of continuous symmetry measure (CSM) values for PS_4 / SO_4 tetrahedral anion groups across different systems: (a) amorphous Li_3PS_4 (2.0 g/cm^3), (b) amorphous Li_3PS_4 (1.8 g/cm^3), (c) α-Li_3PS_4, (d) β-Li_3PS_4, (e) γ-Li_3PS_4, and (f) HT-Li_2SO_4. Throughout the entire trajectory, all tetrahedral anion groups exhibit a nearly perfectly symmetric shape, confirming our treatment of anion groups as rigid bodies. The CSM values were computed using all anion groups in every tenth structure of the trajectory.

Note S4. CorrelationAnalyzer Python Package

All of the analysis tools developed to detect rotational events of anion groups using quaternion representations, detect translational event of lithium-ions, analyze the correlation between rotations and hops are provided in the following Github repository: https://github.com/KyuJungJun/CorrelationAnalyzer
